# Supplementary material for: Understanding pathways to stimulant use: a mixed-methods examination of the individual, social and cultural factors shaping illicit stimulant use across Europe (ATTUNE): study protocol
Source: BMJ Open. 2019 Aug 10;9(8):e029476. doi: 10.1136/bmjopen-2019-029476 (PMC6701668; doi:10.1136/bmjopen-2019-029476)
Supplement: Supplementary data [file bmjopen-2019-029476supp001.pdf]

Use for groups 1-5:
dependent; remitted; frequent; non-dependent; formerly frequent non-dependent, non-frequent

Interview Guideline 'A' – Attune

Interviewer:

This study is about understanding pathways to stimulants use across Europe. You have agreed to participate in an interview as part of this study because you feel you would identify with one of the following statements, would you agree?

a) Are **currently** using amphetamines (Group 1, 2, 3 and 5)
b) **Stopped** using amphetamines (Group 2, 4 and 5)
c) Never used amphetamines but have had the **opportunity** to in the past (Group 6 – USE Guideline B)

Introduction

Tell me a little about yourself and your life now.

(Prompts – Age, individual situation (job, housing, income, education, health situation, emotional well-being), social life (partnership, family, friends) positive and negative impacts)

Drugs and alcohol

What is your experience of drug and alcohol use? [Interviewer use CHART tool for reporting on substances used in life until today]

(Prompts: *personal usage including: age of first use; type of drugs, frequency and quantity, motivations for use/non-use, levels and routes of use; negative impact (dependence, offending, imprisonment)*

What effect are you hoping for from use of these substances?

(Prompts: socialising, coping strategy, behaviour change, health)

Tell me more about the circumstances of your drug and alcohol use since your first use [plot significant moments in the **life events CHART**]

(Prompts: use of drugs/alcohol by others around them; any drug/alcohol offers; individual situation (work, housing, income, health), relationships (family, friends, romantic partners); critical life events (violence, separation from significant other, death, illness, chronic disease)

What is your use of drug and alcohol like now? Has this changed over time? [CHART tool for substances]

(Prompts: *type of substance and frequency of use;*
circumstances for changes (living, employment, education, income, health), lifestyle (leisure), relationships (friends, family, romantic partner, marriage, birth of children)

Stimulants – Firsts, effects, perceptions

What is your experience of Amphetamine Type Substances? E.g. amphetamine, ecstasy, MDMA, methamphetamine [REFER to ATS card and CHART for substances]

(Prompts:, *, personal usage including: age of first use; type of drugs; frequency levels and routes of use; and surrounding circumstances of use/non-use, motivations for use/non-use)*

Use for groups 1-5:
dependent; remitted; frequent; non-dependent; formerly frequent non-dependent, non-frequent

What was life like leading up to Amphetamine Type Stimulants being first used? [plot significant moments in the **life events CHART]**

(Prompt around family, friendships, relationships, education, employment, and social, offending behaviour, health and emotional well-being, housing, significant events)

What effect were you hoping for from these Amphetamine Type Stimulants? Why did you want this effect?

(Prompt: feelings, positive and negative experiences, change inhibitions, focus, control, euphoria)

What was happening in your life when you continue to use XXX (specify the different ATS the person has discussed)? [plot significant moments in the **life events CHART]**

(Prompts: *use of ATS by others around them, any ATS offers or availability* people in their social network and their use/non-use of ATS, *individual situation (work, housing, income, health), relationships (family, friends, romantic partners); critical life events (violence, separation from significant other, death, illness, chronic disease)*

What do you personally think about ATS use?

(Prompts around positive and negative views- physical and mental health, lifestyle, personal plans, offending, social relations)

Stimulants – Source, availability, funding

How would/do you usually get Amphetamine Type Stimulants?
(Prompt: buy from/share with friends, dealer-user interactions, ease of interaction/introduction to dealer)

How easy or difficult are they to get? Give examples

How do you fund your use? Does funding imply any legal problems?
(Prompt: employment, welfare, criminal activity, deal, trouble with the police, criminal justice system)

Stimulants – lifestyle and relationships

How does your ATS use impact on your relationships with others? Give examples

How has your lifestyle changed since you started/stopped using ATS? [plot significant moments in the **life events CHART]**

(**Prompt:** significant life events (individual, social, lifestyle, health and well-being), increases/decreases in substance use since first usage of ATS; protective or harmful factors (dependence, offending)

How has your use of ATS changed over time? [Plot CHART for substances]

(Prompt: *type of substance and frequency of use,* increase in levels, decrease in levels, poly-substance use, changes in routes of administration, context of use

What was happening at the time of the change in use? **[**plot significant moments in the **life events CHART]**

(Prompt:, treatment, other service use, circumstances (living, employment, education, income, health), lifestyle (leisure), relationships (friends, family, romantic partner, marriage, birth of children), significant events

What is important for your life now?

Use for groups 1-5:
dependent; remitted; frequent; non-dependent; formerly frequent non-dependent, non-frequent

(Prompt around family, marriage, children, romantic partners, friends, education, employment, social, offending behaviour, health and emotional well-being, housing, positive and negative impacts)<br>**What do you hope for/expect for your life in the next 5-10 years?**<br>(Prompt: change in substance use (including ATS), change in relationships, health, employment career, lifestyle)

Thank you for taking part in interview

(Check have consent form)

Rosenkranz M, *et al. BMJ Open* 2019; 9:e029476. doi: 10.1136/bmjopen-2019-029476

Use for group 6:
Non-stimulant user

Interview Guideline 'B' – Attune

Interviewer:

This study is about understanding pathways to stimulants use across Europe. You have agreed to participate in an interview as part of this study because you feel you would identify with one of the following statements, would you agree?

a) Are **currently** using amphetamines (Group 1, 2, 3, 5 – use Guideline A)
b) **Stopped** using amphetamines (Group 2, 4 and 5 – use Guideline A)
c) Never used amphetamines but have had the **opportunity** to in the past (Group 6 – USE Guideline B)

Introduction

Tell me a little about yourself and your life now.

(Prompts – Age, individual situation (job, housing, income, education, health situation, emotional well-being), social life (partnership, family, friends) positive and negative impacts)

What is your experience of drug and alcohol use? [Interviewer use CHART tool for reporting on substances used in life until today]

(Prompts: *personal usage including: age of first use; type of drugs, frequency and quantity motivations for use/non-use, levels and routes of use; negative impact (dependence, offending, imprisonment)*

What effect are you hoping for from use of these substances?

(Prompts: socialising, coping strategy, behaviour change, health)

Drugs and alcohol

Tell me more about the circumstances of your drug and alcohol use since your first use [plot significant moments in the **life events CHART**]

(Prompts: use of drugs/alcohol by others around them; any drug/alcohol offers; individual situation (work, housing, income, health), relationships (family, friends, romantic partners); critical life events (violence, separation from significant other, death, illness, chronic disease)

What is your use of drug and alcohol like now? Has this changed over time? [CHART tool for substances]

(Prompts: *type of substance and frequency of use;* circumstances (living, employment, education, income, health), lifestyle (leisure), relationships (friends, family, romantic partner, marriage, birth of children)

Stimulants – Firsts, protective factors, perceptions

What is your experience with the use Amphetamine Type Substances? E.g. amphetamine, ecstasy, MDMA, methamphetamine in your social network?

(Prompts: *use of ATS by others around them, any ATS offers or availability)*

Rosenkranz M, *et al. BMJ Open* 2019; 9:e029476. doi: 10.1136/bmjopen-2019-029476

Use for group 6:
Non-stimulant user

What was happening in your life when XXX (specify the different ATS the person has discussed by using the ATS card) was offered to you (ATS substances)? [plot significant moments in the **life events CHART**]

(Prompt: individual and social circumstances surrounding the non-use health and wellbeing, people in their social network and their use/non-use of ATS, specific life events)

Are others in your networks continuing to use ATS? How does this use impact on your relationship with them? Give examples

(Prompt: changes, life events, availability)

What was it like being around others who were using ATS?

(Prompt: impact on relationships, socialising, networks, and employment)

Has there ever been a situation where you thought about using ATS? [Plot [plot significant moments in the **life events CHART**] **What was happening at this time for you?**

(prompt: lifestyle, relationships, networks, employment, significant events)

How would you explain that you never used these ATS

(Prompt: costs, availability, opportunity, age, circumstances, health, fear of legal problems, fear of dependence, protective factors such as partner, employment, lifestyle, negative impact on other people)

What do you think about ATS use? Has that perception changed or stayed the same over time?

(Prompt around positive and negative views- physical and mental health, lifestyle, personal plans, offending, social relations)

Stimulants – Source, availability, funding

Would you know how to buy Amphetamine Type Stimulants?
(Prompt: buy from/share with friends, dealer-user interactions, ease of interaction/introduction to dealer)

How easy or difficult do you think they are to get? Give reasons

Lifestyle

What is important for your life now?

(Prompt around family, marriage, children, romantic partners, friends, education, employment, social, offending behaviour, health and emotional well-being, housing, positive and negative impacts)

What do you hope for/expect for your life in the next 5-10 years?

(Prompt: change in substance use (including ATS), change in relationships, health, employment career, lifestyle)

Thank you for taking part in interview

(Check have consent form)

Rosenkranz M, *et al. BMJ Open* 2019; 9:e029476. doi: 10.1136/bmjopen-2019-029476
