# Supplementary material for: Understanding pathways to stimulant use: a mixed-methods examination of the individual, social and cultural factors shaping illicit stimulant use across Europe (ATTUNE): study protocol
Source: BMJ Open. 2019 Aug 10;9(8):e029476. doi: 10.1136/bmjopen-2019-029476 (PMC6701668; doi:10.1136/bmjopen-2019-029476)
Supplement: Author's manuscript [file bmjopen-2019-029476.draft_revisions.pdf]

BMJ Open

BMJ Open is committed to open peer review. As part of this commitment we make the peer review history of every article we publish publicly available.

When an article is published we post the peer reviewers' comments and the authors' responses online. We also post the versions of the paper that were used during peer review. These are the versions that the peer review comments apply to.

The versions of the paper that follow are the versions that were submitted during the peer review process. They are not the versions of record or the final published versions. They should not be cited or distributed as the published version of this manuscript.

BMJ Open is an open access journal and the full, final, typeset and author-corrected version of record of the manuscript is available on our site with no access controls, subscription charges or pay-per-view fees (http://bmjopen.bmj.com).

If you have any questions on BMJ Open's open peer review process please email info.bmjopen@bmj.com

BMJ Open

# Understanding Pathways to Stimulant Use: a mixed-methods examination of the individual, social and cultural factors shaping illicit stimulant use across Europe (ATTUNE): study protocol

| | |
|---|---|
| Journal: | *BMJ Open* |
| Manuscript ID | bmjopen-2019-029476 |
| Article Type: | Protocol |
| Date Submitted by the Author: | 28-Jan-2019 |
| Complete List of Authors: | Rosenkranz, Moritz; University Medical Centre Hamburg-Eppendorf, Centre of Interdisciplinary Addiction Research of Hamburg University, Department of Psychiatry<br>O'Donnell, Amy; Newcastle University, Institute of Health and Society<br>Verthein, Uwe; University Medical Centre Hamburg-Eppendorf, Centre of Interdisciplinary Addiction Research of Hamburg University, Department of Psychiatry<br>Zurhold, Heike; University Medical Centre Hamburg-Eppendorf, Centre of Interdisciplinary Addiction Research of Hamburg University, Department of Psychiatry<br>Addison, Michelle; Newcastle University, IHS<br>Liebregts, Nienke; University of Amsterdam, Bonger Institute of Criminology<br>Rowicka, Magdalena; Institute of Applied Psychology, Maria Grzegorzewska Academy of Special Education<br>Barták, Miroslav; Charles University and General University Hospital in Prague, Department of Addictology, First Faculty of Medicine<br>Petruželka, Benjamin; Charles University and General University Hospital in Prague, Department of Addictology, First Faculty of Medicine<br>Kaner, Eileen; Newcastle University, Institute<br>Martens, Marcus-Sebastian; University Medical Centre Hamburg-Eppendorf, Centre of Interdisciplinary Addiction Research of Hamburg University, Department of Psychiatry |
| Keywords: | study protocol, amphetamine type stimulants, drug use trajectory, life course |
| | |

SCHOLARONE™
Manuscripts

# Understanding Pathways to Stimulant Use: a mixed-methods examination of the individual, social and cultural factors shaping illicit stimulant use across Europe (ATTUNE): study protocol

Moritz Rosenkranz[1], Amy O'Donnell[2], Uwe Verthein[1], Heike Zurhold[1], Michelle Addison[2], Nienke Liebregts[3], Magdalena Rowicka[4], Miroslav Barták[5], Benjamin Petruželka[5], Eileen Kaner[2] & Marcus-Sebastian Martens[1]

Centre of Interdisciplinary Addiction Research of Hamburg University, Department of Psychiatry, University Medical Centre Hamburg-Eppendorf, Hamburg, Germany[1]

Institute of Health and Society, Newcastle University, Newcastle upon Tyne, United Kingdom[2]

Bonger Institute of Criminology, University of Amsterdam, Amsterdam, Netherlands[3]

Institute of Applied Psychology, Maria Grzegorzewska Academy of Special Education, Warsaw, Poland[4]

Department of Addictology, First Faculty of Medicine, Charles University and General University Hospital in Prague, Prague , Czech Republic[5]

Corresponding author:
Moritz Rosenkranz
Centre of Interdisciplinary Addiction Research of Hamburg University, Department of Psychiatry, University Medical Centre Hamburg-Eppendorf, Martinistr. 52, 20246 Hamburg, Germany.
Tel.: +49 40 42838-6383
E-mail: moritz.rosenkranz@uni-hamburg.de

Word count: 3480

Key words: study protocol, amphetamine type stimulants, drug use trajectory, life course



For peer review only - http://bmjopen.bmj.com/site/about/guidelines.xhtml

## ABSTRACT

### Introduction

Amphetamine Type Stimulants (ATS) are the second most commonly used illicit drugs globally. Yet, there is little evidence on which factors are associated with the development of different patterns of ATS use over the life course. This study aims to examine which individual, social, and environmental factors shape different pathways and trajectories of ATS consumption. The study will be conducted in five European countries: Germany; the Netherlands; Poland; Czech Republic and the United Kingdom.

### Methods and analysis

We will use a sequential mixed methods study design to investigate the multiple factors (familial, social and occupational situation, critical life events, general risk behaviour, mental and physical health, satisfaction with life) that shape individual ATS use pathways. A systematic literature review will be performed to provide an overview of the current academic literature on the topic. In Module 1, qualitative semi-structured interviews (n=ATS users and non-users) will be conducted to explore individual experiences of, and perspectives on, dynamics of change in stimulant consumption patterns. In Module 2, structured questionnaires (n=2000 ATS users and non-users) will be administered via tablet computers, to validate and enhance the generalisability of the interview findings. Data integration will take place at two key points. First, during the study, where the findings from the first qualitative interviews will inform the design of the structured questionnaire. Second, at the end of the study, where mixed methods data will be brought together to generate an in-depth, contextualised understanding of the research topic.

### Ethics and dissemination

The study has been approved by the respective responsible ethics committee in each participating country. Data will be treated confidentially to ensure participants' anonymity. Findings will be disseminated in peer reviewed scientific journals, national and international conferences, and in briefings for policy and practice.


## SUMMARY

### Strengths and limitations of this study

- First study of its kind to examine different pathways of ATS use, and will thus expand an underdeveloped evidence base.
- Large qualitative and quantitative dataset collected in five European countries, which allows valuable inter-country comparisons.
- Applies a theory-based analytical framework to understand the individual, social and environmental influences shaping ATS use, which will provide important insights expedient for the development of tailored prevention and intervention measures.
- Cross-sectional design allows correlative but no causal conclusions.



For peer review only - http://bmjopen.bmj.com/site/about/guidelines.xhtml

For peer review only

# INTRODUCTION

Amphetamine Type Stimulants (ATS) are the second most commonly used illicit drugs globally[1] as well as in Europe[2]. ATS include amphetamines ("speed"), Methylenedioxymethamphetamine ("MDMA" or "ecstasy"), methamphetamine ("crystal meth")[3] and illicit use of amphetamine-type prescription drugs (e.g. "Ritalin®"). In recent years, there has been a rise in new psychoactive substances that mimic the effects of stimulants in global drug markets[4], including synthetic cathinones such as mephedrone ("bath salts"). Across Europe as a whole, 0.5% of those aged 15-64 reported using amphetamines in the past 12 months, with higher rates for MDMA use (0.8%). However, ATS use rates vary by country, with the highest consumption found in the Netherlands (amphetamine, 1.7%; MDMA, 3.6%). Data also suggest higher consumption rates for adolescents and younger adults compared to the general population, with one percent of those aged 15-34 reporting amphetamine consumption, and 1.8% MDMA consumption, over the previous year[1 2]. Worldwide, quantities of ATS seized have doubled over the past decade[5], with increased usage levels reflected in wastewater analyses conducted in several European countries[6], and in a corresponding rise in numbers of first-time entrants for stimulant treatment across Europe, from about 7000 in 2006 to more than 12000 in 2016[2].

Methamphetamine use at dependent levels is associated with multiple co-morbidities, including HIV infection, hepatitis, cardiac effects, cognitive dysfunction, and prominent psychiatric consequences such as psychosis[7 8]. Although MDMA is often viewed as a recreational drug, prolonged use is associated with neurological dysfunction and depression[9]. Additional societal costs identified with ATS abuse include premature death, crime, lost productivity, environmental damage, disruption of family life, and infectious disease[7 10 11]. However, despite the substantial harms associated with ATS use, increased prevalance in consumption rates, and rising number of treatment entries, there is remarkably little evidence regarding which factors shape different patterns of ATS use.

Some qualitative and very few quantitative studies exploring influences on ATS use have been published, mostly focussed on factors affecting initiation[12]. Important motives for the initiation of ATS consumption identified in previous research include: curiosity or propensity for experimentation[13-16]; self-management of stress, trauma, or other mental health issues[14 16-22]; and to boost of performance at work/studies[17 19 23-25] or in private settings (sexual relationships, endurance at dance events)[16 24 26-28]. Literature exploring what influences different trajectories of ATS consumption following initiation remains extremely limited however. Evidence suggests that continued and/or increased consumption is often to support specific functional needs (improvement of stress management or reduced insecurity in social situations) and to help manage withdrawal effects[13 18 20 25 27 29-32]. The experience of critical life events (separation, death of a close person, domestic violence) also appears to be associated with continued consumption[17 24 30]. Reported factors connected with a decrease (in some cases until abstinence) included an increased perception of negative health impacts[14 18 27 33 34], changing social networks, and reduced availability of ATS[26 35 36]. However,



For peer review only - http://bmjopen.bmj.com/site/about/guidelines.xhtml

few studies have been published focussed on stimulant-based NPS and there remains extremely limited evidence exploring users' perspectives on which factors enable decreased ATS use or longer term abstinence.

In the framework of the European Research Area Network on Illicit Drugs (ERANID), a consortium of five research institutions from Germany, the United Kingdom, Poland, the Netherlands and the Czech Republic, was formed to conduct a study to respond to this evidence gap. The European ATTUNE study (Understanding Pathways to Stimulant Use: a mixed-methods examination of the individual, social and cultural factors shaping illicit stimulant use across Europe) is led by Germany, as the principal investigator.

**Research objectives**

The overall aim of the project is to improve our understanding of which factors shape different pathways of ATS use in Europe. By examining interactions between individual, social and environmental influencing factors, and the overall trajectory of drug use, this study will explore individual motivations to use ATS and describe different patterns of consumption over time. In doing so, the study seeks to identify potential protective factors (e.g. personality traits, social integration) associated with the ability to control, decrease or quit ATS use, as well as risk factors (e.g. critical life events) associated with the escalation of ATS consumption patterns towards problematic use and/or dependence. Further, we aim to explore why some individuals exposed to ATS select not to use these substances, as well as examining the relationship that illicit stimulant users have with other illicit and licit substances.

## METHODS AND ANALYSIS

**Study design**

ATTUNE is a sequential, exploratory, mixed-methods study. The design, implementation and interpretation of the study is informed by the biopsychosocial model of substance use[37]. This model suggests that the change process of drug use pathways is influenced by the interaction of three core domains: individual differences, social dynamics and the environmental/cultural setting. The study comprises of three main components.

First, a systematic review of the qualitative and quantitative published literature on which individual, social and environmental influences shape different pathways of ATS use over the life course. The findings from these reviews aim to provide an insight into the existing international literature that examines ATS users' perspectives on why they start, stop, increase, and/or reduce their ATS consumption, whilst also exploring under which circumstances and conditions stimulant users change their consumption patterns[12].

Next, in Module 1 of the fieldwork, qualitative methods (semi-structured interviews) will be used to explore individual experiences of and perspectives on dynamics of change in stimulant consumption patterns. The topic guide will build in particular on the findings from



For peer review only - http://bmjopen.bmj.com/site/about/guidelines.xhtml

the review of qualitative literature conducted at the start of the project, as well as the theoretical underpinning for the research (biopsychosocial model of drug use).

Finally, in Module 2, the findings from the qualitative interviews will inform the development of a structured questionnaire in order to validate and enhance the generalisability of the results in a large sample of ATS users and non-users.

The total length of the study is 36 months (September 2016 to August 2019). The estimated duration of the recruitment as well as data collection for Module 1 is seven months, and for Module 2 it will amount to 13 months.

**Participants**

Eligibility criteria

Individuals who have either used or had the opportunity to use ATS are eligible for inclusion in both the qualitative interviews and survey questionnaire. This includes people who have either consumed ATS at least once in their life, or people who have never used ATS but have been exposed to ATS consumption (defined as having been present when family or friends took ATS but refused to consume themselves).

To ensure the inclusion of only those who have had the opportunity to experience changes in the trajectory of their ATS use, the participant´s first ATS consumption (or exposure) needs to have taken place at least five years before the interview or survey questionnaire.

Further inclusion criteria:

- No lifetime opioid dependency
- Aged 18 years or older
- Resident in one of the five national sampling regions
- Able to take part in the interview (not psychotic, no severe cognitive impairments or language barriers).

A screening website will be set up where interested persons can check their eligibility to participate in an interview or survey questionnaire. If a person is screened successfully, a message is displayed inviting the person to participate and offering different possibilities to contact the research team to arrange an interview appointment. A randomly chosen screening ID facilitates the connection of the screening data with the interview whilst maintaining anonymity.

Study Groups

Participants in both Module 1 and Module 2 will be recruited using convenience sampling (see below). To ensure a sufficient variety of ATS use patterns or ATS use trajectories, six study groups were predefined for Module 1 and five study groups for Module 2. This



For peer review only - http://bmjopen.bmj.com/site/about/guidelines.xhtml

For peer review only

approach provides a stratified sample, in which each ATS use pattern (group) serves as a stratum.

*Module 1: Qualitative semi-structured interviews*

Eligible participants meeting the above criteria will be allocated to one of six study groups depending on their ATS consumption patterns (time, frequency, dependency). Table 1 shows an overview of the operationalisation of the six groups.

**Table 1: Operationalization of Study Groups in Module 1**

| Study groups in Module 1 | Name | past 12 months prevalence | ≥ 10 consumption days within past 12 months | ≥ 10 consumption days within one year (at any time except past 12 months) | currently ATS dependent | formerly ATS dependent |
|---|---|---|---|---|---|---|
| Group 1 | Currently dependent users | yes | yes | n.a. | yes | n.a. |
| Group 2 | Formerly dependent users | n.a. | n.a. | yes | no | yes |
| Group 3 | Currently frequent, non-dependent users | yes | yes | n.a. | no | no |
| Group 4 | Formerly frequent, non-dependent users | no | n.a. | yes | no | no |
| Group 5 | Non-frequent users (currently or formerly) | no | n.a. | no | no | no |
| Group 6 | Exposed non-users | n.a. | n.a. | n.a. | n.a. | n.a. |

*Module 2: Survey questionnaire*

Eligible participants meeting the above criteria will be allocated to one of five study groups depending on their ATS consumption patterns (time, frequency, dependency). Table 2 shows an overview of the operationalisation of the five groups.

**Table 2: Operationalization of study groups in Module 2**

| Study groups in Module 1 | Name | past 12 months prevalence | past 3 months prevalence | ≥ 10 consumption days within past 12 months | ≥ 10 consumption days within one year (at any time except past 12 months) |
|---|---|---|---|---|---|
| Group A_1 | Currently frequent users | yes | yes | yes | n.a. |
| Group A_2 | Currently non-frequent users | yes | yes | no | n.a. |
| Group B_1 | Formerly frequent users | no | n.a. | n.a. | yes |
| Group B_2 | Formerly non-frequent users | no | n.a. | n.a. | no |
| Group C | Exposed non-users | n.a. | n.a. | n.a. | n.a. |

## Sample size

*Module 1: Qualitative semi-structured interviews*

45 persons per study group (n=270 for the total sample, see table 3) were considered sufficient for Module 1[38]. As we plan to recruit participants via purposeful sampling[39], we expect to generate data that are rich enough to answer our research questions and inform the questionnaire used in Module 2 of the study.



For peer review only - http://bmjopen.bmj.com/site/about/guidelines.xhtml

**Table 3: Sample sizes Module 1 by countries and study groups**

| Country | Partner institution | Data collection regions | Sample sizes | | | | | | |
|---|---|---|---|---|---|---|---|---|---|
| | | | Group 1 | Group 2 | Group 3 | Group 4 | Group 5 | Group 6 | **Total N countries** |
| | | | Currently dependent | Formerly depen-dent | Currently frequent, non-dependent | Formerly frequent, non-dependent | Non-frequent (currently or formerly) | Exposed | |
| Germany | ZIS | Border Region to Czech Republic /metropolitan region of Hamburg | 10 | 10 | 10 | 10 | 10 | 10 | **60** |
| United Kingdom | UNEW | Northern England | 10 | 10 | 10 | 10 | 10 | 10 | **60** |
| Poland | APS | Metropolitan region of Warsaw | 10 | 10 | 10 | 10 | 10 | 10 | **60** |
| Netherlands | RG | Amsterdam/the region of Eindhoven | 10 | 10 | 10 | 10 | 10 | 10 | **60** |
| Czech Republic | OGCR | Border region to Germany | 5 | 5 | 5 | 5 | 5 | 5 | **30** |
| **Total N groups** | | | **45** | **45** | **45** | **45** | **45** | **45** | **270** |

*Module 2 Survey questionnaire*

Statistical analyses within the group of current users (A_1 and A_2) as well as within the group of former users (B_1 and B_2) are planned (see table 2). To facilitate this at country level, a group size of n=100 is sufficient to detect statistically significant small to medium effects for continuous distributed variables (d≥.40, α=0.05, power=80%). The sample sizes of the groups are planned as follows. For both, groups A and B, up to 200 participants in each country will be recruited. This allows us to analyse subgroups, e.g. dependent and non-dependent users in groups A as well as B. As group C consists of non-users only, the analyses will focus on comparison with one of the user groups (A or B), reducing the required size for this group to n=100. The total sample size for Module 2 for all countries will amount to 2000 persons. Due to funding restraints, the sample size in the Netherlands and the Czech Republic is smaller, which might impede the production of statistically significant country specific intra-group comparisons.

**Table 4: Sample sizes Module 2 by countries and study groups**

| Country | Partner institution | Data collection regions | Sample sizes | | | | | |
|---|---|---|---|---|---|---|---|---|
| | | | **Current ATS user** | | **Former ATS user** | | **Non ATS user** | **Total N countries** |
| | | | A_1: Frequent | A_2: Non-frequent | B_1: Frequent | B_2: Non-frequent | C: exposed | |
| Germany | ZIS | Border Region to Czech Republic /metropolitan region of Hamburg | 100 | 100 | 100 | 100 | 100 | **500** |
| United Kingdom | UNEW | Northern England | 100 | 100 | 100 | 100 | 100 | **500** |
| Poland | APS | Metropolitan region of | 100 | 100 | 100 | 100 | 100 | **500** |



For peer review only - http://bmjopen.bmj.com/site/about/guidelines.xhtml

| | | | | | | | | |
|---|---|---|---|---|---|---|---|---|
| | | Warsaw | | | | | | |
| Netherlands | RG | Amsterdam/the region of Eindhoven | 50 | 50 | 50 | 50 | 50 | **250** |
| Czech Republic | OGCR | Border region to Germany | 50 | 50 | 50 | 50 | 50 | **250** |
| **Total N groups** | | | **400** | **400** | **400** | **400** | **400** | **2000** |

## Recruitment

### Sampling method and recruitment procedure

Participants in both Modules will be recruited by non-probability (convenience) sampling, which is an accepted means of accessing participants from 'hard-to-reach' as well as minority populations[40 41]. Pre-identified sampling areas in each participating country (see table) are designed to include participants living in urban as well as rural areas.

Multiple methods and sites will be employed to identify and recruit participants for both Modules. Leaflets and posters containing information about the study, a link to the screening website, and contact details of the research teams, will be printed and distributed in substance use help and treatment facilities, on university black boards, in head shops, bars and nightclubs. Social media and substance use web forums will be used to share and circulate the request for participants. Participants will also be recruited actively at university campuses, drug service facilities and music festivals by directly approaching potential participants. All interested interviewees will be screened for eligibility prior to the interview. If eligible to participate, each respondent will receive a screening-code, and the interview can either be conducted straight away, or an appointment can be made for a later date. At the end of each interview participants will be asked to recruit further participants from their social network. This additional snowball sampling approach will be realized by handing over study leaflets, as well as up to three numbered cards containing contact details of the research team. The numbers on the cards will help to track the snowball sampling approach.

## Procedure

### Fieldwork

*Module 1*

Face-to-face semi-structured interviews will be conducted by members of the research team of each participating country. Prior to the interview, all participants will receive an information leaflet containing details about the study, explaining what participation involves: anonymity, confidentiality, the use of data, and data protection rules. The participants will then be invited to complete a verbal consent form, should they wish to participate. The interview will be audio recorded and will last approximately 45 to 60 minutes. On completion of the interview, each participant will receive an incentive (money or vouchers, depending on country). All interviews will be transcribed in full, transferred to appropriate software for analysis, and the audio file deleted.

*Module 2*

For peer review only



For peer review only - http://bmjopen.bmj.com/site/about/guidelines.xhtml

The quantitative survey will be conducted with the CAPI method (Computer-assisted personal interviewing). The questionnaire content will first be developed in Microsoft Word. Once finalised, all questions will be programmed using survey software GessQ® to enable administration via password-protected tablet computers. The survey instrument will be translated and piloted in all partner countries and revised as necessary. The latest version of the questionnaire will be hosted on a central server operating in the IT environment of Hamburg University and can be downloaded to the tablets directly as necessary. This server is also the recipient for the data uploads from the tablets.

In each country, trained research assistants will recruit participants and conduct the interviews face-to-face or via video-telephony (Skype®). Show cards containing relevant prompts and additional information (e.g. lists of ATS, answering scales) will support the conduct of the interviews.

Monitoring and data management

The screening process and fieldwork progress will be monitored using a study-specific coordination database. This database will be populated with screening data from each participant as well as key information regarding the interviews or questionnaires, such as study group, duration of interview, gender distribution, sampling region, and contact with drug help services.

*Module 1*

The raw qualitative data from Module 1 will be managed, stored and analysed by the respective research teams in each participating country. Initial analyses will be conducted at country level using a common coding framework. Analysed data, including emerging interview themes, will be pooled, and provided to ATTUNE PI (Hamburg University).

*Module 2*

The raw quantitative data will be uploaded continuously to the central data management at the Hamburg University. The raw data will be cleaned, edited and transformed into SPSS datasets, one for each country, as well as one comprehensive dataset covering all five countries.

**Survey instruments**

*Module 1: Interview guideline and time sheet*

Two semi-structured interview guides will be used to conduct the in-depth interviews in Module 1: one for the ATS user groups plus an adapted topic guide for the non-user group. The topic guide will be based on key emergent themes from the systematic literature review as well as relevant theoretical considerations (biopsychosocial model of substance use). Participants will be asked about their experiences and their consumption patterns regarding ATS and other licit and illicit drugs. To obtain a detailed understanding of which influences have shaped these ATS use patterns, participants will be asked about drug use motives, effects/consequences, settings and occasions, and how these have changed during the



For peer review only - http://bmjopen.bmj.com/site/about/guidelines.xhtml

period(s) in life where ATS use took place. The interview will end with questions about the (social) setting of use and its impact and the integration of ATS use into the respondent´s lifestyle. Whilst the use of the interview guide will ensure that all central topics are covered, participants will have the opportunity to discuss additional issues or concerns where relevant. During the interviews, researchers will chart participants substance use over time, including age of onset, frequency and life stage, as well as positive and negative life events (e.g. family/partnership, education/work, illness, treatment, imprisonment).

*Module 2: Quantitative questionnaire*

The quantitative survey questionnaire will include questions based on the key themes emerging from the qualitative interviews, as well as a selection of standardised instruments to assess various substance use, health and psychological factors (see table 5). The questionnaire will cover:

a. Socio-demographics: These include sex, age, citizenship, migration background, relationship and children, living situation, educational and occupational situation, and social integration.
b. Drug use: Detailed assessment of all illicit drugs ever used in life (lifetime prevalence, past 12-month prevalence, past 30 days prevalence, age at first and last use), test for alcohol dependence and tobacco smoking status.
c. ATS use: Test for ATS dependence, injecting drug use and treatment experiences; usual setting of ATS use; patterns, motives and consequences of ATS use including (reasons for) changes.
d. Judicial problems: times and reasons for imprisonment
e. Physical and mental health assessment
f. Personality assessment and critical life events

For group C (non-users), some questions will be omitted (e.g. questions about ATS use), and replaced with alternative questions focussed on their motives for non-use and exposure situation.

**Table 5: Overview of standardised measurement instruments**

| Name | Acronym | Content |
|---|---|---|
| International Standard Classification of Education[42] | ISCED | Identification of highest educational level |
| Subjective social integration[43] | SSI | Subjective assessment of social integration |
| Subjective social position[43] | SSP | Subjective assessment of social position |
| CAGE questionnaire[44] | CAGE | Alcohol problems lifetime |
| Alcohol Use Disorders Identification Test[45] | AUDIT-C | Alcohol problems past year |
| The Severity of Dependence Scale[46] | SDS | ATS dependency lifetime |
| Brief Symptom Inventory-18[47] | BSI-18 | Measurement of somatization, anxiety, depression |
| Satisfaction with life scale[48] | SWLS | General life satisfaction |
| Big Five Inventory[49] | BFI-10 | Assessment of five personality traits |
| Brief Sensation Seeking Scale[50] | BSSS-4 | Measurement of sensation seeking |
| Generalized Self-Efficacy scale[51] | GSE | Measurement of self-efficacy |
| Connor-Davidson Resilience Scale[52] | CD-RISC-10 | Measurement of resilience |

## Planned analyses



For peer review only - http://bmjopen.bmj.com/site/about/guidelines.xhtml

*Module 1*

Qualitative data from the semi-structured interviews will be analysed using content analysis, and conducted with appropriate software, such as MAXQDA[53] or NVivo[54]. Partners will develop a common, unified coding system to facilitate comparable findings from the interviews across all partner countries. In addition, the data collected with the timeline and chart on substance use and life events will be merged and analysed systematically. Each partner will conduct initial country level analysis separately, in the respective national language. Once this initial analysis has been completed, each partner will produce a report detailing the country-level findings, in English and following a common template. These reports will then be compiled and synthesised to produce a comprehensive, cross-national analysis of the qualitative interviews.

*Module 2*

The quantitative data analysis will be conducted using the statistical software package SPSS 22[55]. Descriptive, univariate analyses will be used to describe the socio-demographics, health and personality assessment characteristics of the sample, alongside "consumption careers" (substance use, motives of use, and changes of use patterns). Independent sample t-tests and $\chi^2$-analyses, corrected for multiple testing, will be conducted to compare gender and age characteristics among the ATS user groups. Multivariate approaches (regressions, ANOVAs) will be used to assess relations between different ATS user groups and a wide range of possible factors that might shape the use patterns as well as the change of these patterns. If data permit, we will calculate latent class analyses to identify ATS user groups which characteristics are unknown yet. Analyses will be conducted at country level, as well as across the full European sample.

## Ethics and dissemination

Based on the regulations in each participating country, ethical approval will be obtained. Participant anonymity will be maintained in both the semi-structured interviews and the survey questionnaire. During the survey, no information will be collected that could link the data to the participant concerns. All participants will be explicitly asked to provide informed consent to taking part in the study and made aware of the data protection rules. Consent will be documented within the interview.

Research findings of ATTUNE will be disseminated in peer reviewed, open access journals as well as at national and international conferences. Each partner will also deliver reports to their funding institutions under the specific terms of the respective country.

## Strengths and limitations

By using multiple methods (systematic literature reviews, qualitative interviews and survey questionnaires), this study will generate in-depth, contextualised evidence in an under-explored field of research. The use of stratified sampling will ensure a sufficient variety of



For peer review only - http://bmjopen.bmj.com/site/about/guidelines.xhtml

For peer review only

types of ATS users and non-users are included, reflecting different use patterns, current and ex-users, dependent and non-dependent users as well as exposed non-users. In particular, the views and experiences of non-users are rarely reflected in the evidence base, and could help generate novel insights into which factors shape decisions not to consume ATS. The application of standardised interview guides and questionnaires will result in a large comprehensive sample that will allow us to compare ATS use in multiple and varied socio-cultural, political and legal environments across Europe.

A key limitation of the study is the cross-sectional design, which makes it difficult to trace pathways and trajectories of ATS use over time. We address this by only including persons whose first contact with ATS consumption occurred at least five years ago, meaning they have had the chance to develop different ATS use patterns. However, it is important to stress that a cross-sectional design allows correlative but no causal conclusions. One further issue concerns the convenience sampling approach employed in this study in predefined sampling regions. At the same time, compared to representative general population surveys, our method should ensure increased levels of inclusion of different ATS user types, allowing for more detailed and in-depth insights into different use trajectories. Finally, by defining multiple inclusion criteria as well as precise group strata, we aim to mitigate potential bias that could emerge from this sampling approach.

## Implications for interventions and future policy

The findings from this research will enable policy makers and practitioners to improve existing ATS prevention and intervention programmes, and support the development of new approaches in the future. By examining different types of stimulant users (including ex-users and non-users), information will be generated which will be important for universal prevention (targeting general populations), selective prevention (focussing on vulnerable groups), and indicated prevention (aiming at groups that show early signs of problematic substance use). Furthermore, given the limited long-term efficacy of ATS treatment[56], the findings of this study could support the development of ATS treatment programmes that are more effectively tailored to the needs of specific ATS populations and individual users.



For peer review only - http://bmjopen.bmj.com/site/about/guidelines.xhtml

*Authors' contribution:* MR, MSM, HZ, UV, AOD and MA designed the study. All other authors contributed to the design of the study. MR wrote the study protocol with substantial critical input from all co-authors. All authors read and approved the final version of the manuscript.

*Funding:* The superordinate research framework ERANID is funded by the European Union under the 7th Framework Programme. Each project partner receives funding from its national funding bodies (ministry of health).

*Competing interests:* Uwe Verthein received a speaker's honoraria and travelling expenses from Mundipharma GmbH. Amy O'Donnell was funded by an NIHR School for Primary Care Research Fellowship between October 2015 and September 2017. All other authors declared not to have competing interests.

*Ethics approval:* In GER, UK, PL, CZ, the study has been reviewed and approved by the respective responsible ethics committee, in NL no ethical approval was required. The respective reference numbers are as follows: GER: WF-03/17; UK: 17/NE/0283; PL: 160_2017/2018; CZ: 180326_EK-NMS.

For peer review only



For peer review only - http://bmjopen.bmj.com/site/about/guidelines.xhtml

## REFERENCES

1. UNODC. United Nations Office On Drugs And Crime. World Drug Report 2018. Vienna: United Nations, 2018.
2. EMCDDA. European Monitoring Centre for Drugs and Drug Addiction. European Drug Report 2018: Trends and Developments. Publications Office of the European Union. Luxembourg: European Monitoring Centre for Drugs and Drug Addiction, 2018.
3. EMCDDA. EMCDDA –Europol joint publications: Amphetamine. Lisbon: European Monitoring Centre for Drugs and Drug Addiction 2011.
4. EMCDDA. European Monitoring Centre for Drugs and Drug Addiction and Eurojust. New psychoactive substances in Europe: Legislation and prosecution. Lisbon: EMCDDA & Eurojust, 2017.
5. UNODC. United Nations Office On Drugs And Crime. World Drug Report 2017. Vienna: United Nations, 2017.
6. EMCDDA. European Monitoring Centre for Drugs and Drug Addiction: Wastewater analysis and drugs: a European multi-city study. Perspectives on Drugs. Lisbon, 2018.
7. Gonzales R, Mooney L, Rawson R. The Methamphetamine Problem in the United States. *Annual Review of Public Health* 2010;31(1):385-98. doi: 10.1146/annurev.publhealth.012809.103600
8. Degenhardt L, Degenhardt L, Roxburgh A, et al. The epidemiology of methamphetamine use and harm in Australia. *Drug and Alcohol Review* 2008;27(3):243-52. doi: 10.1080/09595230801950572
9. Kelly BC. Mediating MDMA-Related Harm: Preloading and Post-loading Among Ecstasy-Using Youth. *Journal of Psychoactive Drugs* 2009;41(1):19-26. doi: 10.1080/02791072.2009.10400671
10. Home Office. Psychoactive substances ban 6 months on: almost 500 arrests and first convictions. . Retrieved from https://www.gov.uk/government/news/psychoactive-substances-ban-6-months-on-almost-500-arrests-and-first-convictions, 2016.
11. Addison M, Stockdale K, McGovern R, et al. Exploring the intersections between novel psychoactive substances (NPS) and other substance use in a police custody suite setting in the north east of England. *Drugs: Education, Prevention and Policy* 2017:1-7. doi: 10.1080/09687637.2017.1378620
12. O'Donnell A, Addison M, Spencer L, et al. Which individual, social and environmental influences shape key phases in the amphetamine type stimulant use trajectory? A systematic narrative review and thematic synthesis of the qualitative literature. *Addiction (Abingdon, England)* 2018 doi: 10.1111/add.14434 [published Online First: 2018/09/04]



For peer review only - http://bmjopen.bmj.com/site/about/guidelines.xhtml

13. Duff C, Moore D. Evading and embracing normality: estrangement and ambivalence in the accounts of methamphetamine consumers. *Critical Public Health* 2015;25(4):488-503. doi: 10.1080/09581596.2014.913785
14. Levy KB, O'Grady KE, Wish ED, et al. An in-depth qualitative examination of the ecstasy experience: results of a focus group with ecstasy-using college students. *Substance use & misuse* 2005;40(9-10):1427-41. doi: 10.1081/ja-200066810 [published Online First: 2005/07/29]
15. Van Hout MC, Brennan R. Plant food for thought: A qualitative study of mephedrone use in Ireland. *Drugs: Education, Prevention and Policy* 2011;18(5):371-81. doi: 10.3109/09687637.2010.537713
16. Ding Y, He N, Detels R. Circumstances of initiation into new-type drug use among adults in Shanghai: are there differences by types of first new-type drug used? *Drug and alcohol dependence* 2013;131(3):278-83. doi: 10.1016/j.drugalcdep.2012.12.019 [published Online First: 2013/01/15]
17. Boeri MW, Harbry L, Gibson D. A Qualitative Exploration of Trajectories Among Suburban Users of Methamphetamine. *Journal of ethnographic and qualitative research* 2009;3(3):139-51. [published Online First: 2009/01/01]
18. Fast D, Small W, Wood E, et al. Coming 'down here': young people's reflections on becoming entrenched in a local drug scene. *Social science & medicine (1982)* 2009;69(8):1204-10. doi: 10.1016/j.socscimed.2009.07.024 [published Online First: 2009/08/25]
19. Sherman SG, German D, Sirirojn B, et al. Initiation of methamphetamine use among young Thai drug users: a qualitative study. *The Journal of adolescent health : official publication of the Society for Adolescent Medicine* 2008;42(1):36-42. doi: 10.1016/j.jadohealth.2007.07.002 [published Online First: 2007/12/25]
20. Cheney AM, Newkirk CN, Nekhavhambe VM, et al. Effects of social and spatial contexts on young latinas' methamphetamine use initiation. *Journal of ethnicity in substance abuse* 2018;17(1):32-49. doi: 10.1080/15332640.2017.1362721 [published Online First: 2017/10/17]
21. Smirnov A, Najman JM, Hayatbakhsh R, et al. Young adults' recreational social environment as a predictor of ecstasy use initiation: findings of a population-based prospective study. *Addiction (Abingdon, England)* 2013;108(10):1809-17. doi: 10.1111/add.12239 [published Online First: 2013/05/15]
22. Cheng WS, Garfein RS, Semple SJ, et al. Binge use and sex and drug use behaviors among HIV(-), heterosexual methamphetamine users in San Diego. *Substance use & misuse* 2010;45(1-2):116-33. doi: 10.3109/10826080902869620 [published Online First: 2009/12/23]



For peer review only - http://bmjopen.bmj.com/site/about/guidelines.xhtml

23. Boeri MW, Sterk CE, Elifson KW. Baby Boomer Drug Users: Career Phases, Social Control, and Social Learning Theory*. *Sociological Inquiry* 2006;76(2):264-91. doi: doi:10.1111/j.1475-682X.2006.00154.x
24. Ho HT, Le GM, Dinh TT. Female sex workers who use amphetamine-type stimulants (ATS) in three cities of Vietnam: use and sexual risks related to HIV/AIDS. *Global public health* 2013;8(5):552-69. doi: 10.1080/17441692.2013.790459 [published Online First: 2013/05/18]
25. von Mayrhauser C, Brecht ML, Anglin MD. Use ecology and drug use motivations of methamphetamine users admitted to substance abuse treatment facilities in Los Angeles: an emerging profile. *Journal of addictive diseases* 2002;21(1):45-60. [published Online First: 2002/02/08]
26. Bourne A, Reid D, Hickson F, et al. "Chemsex" and harm reduction need among gay men in South London. *The International journal on drug policy* 2015;26(12):1171-6. doi: 10.1016/j.drugpo.2015.07.013 [published Online First: 2015/08/25]
27. Eiserman JM, Diamond S, Schensul JJ. "Rollin' on E": a qualitative analysis of ecstasy use among inner city adolescents and young adults. *Journal of ethnicity in substance abuse* 2005;4(2):9-38. doi: 10.1300/J233v04n02_02 [published Online First: 2005/11/09]
28. Parsons JT, Kelly BC, Weiser JD. Initiation into methamphetamine use for young gay and bisexual men. *Drug and alcohol dependence* 2007;90(2-3):135-44. doi: 10.1016/j.drugalcdep.2007.02.017 [published Online First: 2007/04/03]
29. Carbone-Lopez K, Owens JG, Miller J. Women's "Storylines" of Methamphetamine Initiation in the Midwest. *Journal of Drug Issues* 2012;42(3):226-46. doi: 10.1177/0022042612456013
30. Kerley KR, Copes H, Griffin OH. Middle-Class Motives for Non-Medical Prescription Stimulant Use among College Students. *Deviant Behavior* 2015;36(7):589-603. doi: 10.1080/01639625.2014.951573
31. Bavarian N, Flay BR, Ketcham PL, et al. Illicit use of prescription stimulants in a college student sample: a theory-guided analysis. *Drug and alcohol dependence* 2013;132(3):665-73. doi: 10.1016/j.drugalcdep.2013.04.024 [published Online First: 2013/05/21]
32. Sterk CE, Theall KP, Elifson KW. Getting into ecstasy: comparing moderate and heavy young adult users. *Journal of psychoactive drugs* 2007;39(2):103-13. doi: 10.1080/02791072.2007.10399869 [published Online First: 2007/08/21]
33. Bungay V, Malchy L, Buxton JA, et al. Life with jib: A snapshot of street youth's use of crystal methamphetamine. *Addiction Research & Theory* 2006;14(3):235-51. doi: 10.1080/16066350500270901

For peer review only

34. German D, Sherman SG, Sirirojn B, et al. Motivations for methamphetamine cessation among young people in northern Thailand. *Addiction (Abingdon, England)* 2006;101(8):1143-52. doi: 10.1111/j.1360-0443.2006.01490.x [published Online First: 2006/07/28]
35. O'Brien AM, Brecht M-L, Casey C. Narratives of Methamphetamine Abuse: A Qualitative Exploration of Social, Psychological, and Emotional Experiences. *Journal of Social Work Practice in the Addictions* 2008;8(3):343-66. doi: 10.1080/15332560802224469
36. Sexton RL, Carlson RG, Leukefeld CG, et al. Trajectories of Methamphetamine Use in the Rural South: A Longitudinal Qualitative Study. *Human Organization* 2008;67(2):181-93.
37. Engel GL. The need for a new medical model: a challenge for biomedicine. *Science (New York, NY)* 1977;196(4286):129-36. [published Online First: 1977/04/08]
38. Malterud K, Siersma VD, Guassora AD. Sample Size in Qualitative Interview Studies: Guided by Information Power. *Qualitative health research* 2015 doi: 10.1177/1049732315617444 [published Online First: 2015/11/29]
39. Palinkas LA, Horwitz SM, Green CA, et al. Purposeful sampling for qualitative data collection and analysis in mixed method implementation research. *Administration and policy in mental health* 2015;42(5):533-44. doi: 10.1007/s10488-013-0528-y
40. Etikan I, Musa Sa, Alkassim RS. Comparison of Convenience Sampling and Purposive Sampling. *American Journal of Theoretical and Applied Statistics* 2016;5(1):1-4. doi: 10.11648/j.ajtas.20160501.11
41. Shaghaghi A, Bhopal RS, Sheikh A. Approaches to Recruiting 'Hard-To-Reach' Populations into Research: A Review of the Literature. *Health promotion perspectives* 2011;1(2):86-94. doi: 10.5681/hpp.2011.009
42. UNESCO. International Standard Classification of Education (ISCED) 2011 [Available from: http://uis.unesco.org/en/topic/international-standard-classification-education-isced accessed 05.12.2018.
43. Beste J, Bethmann A, Gundert S. Social Structure and Living Conditions: Material and Social Situation of Unemployment Benefit Recipients. In: Research IfE, ed. IAB Summary Reports. Nuremberg, 2014:8.
44. Ewing JA. Detecting alcoholism. The CAGE questionnaire. *Jama* 1984;252(14):1905-7. [published Online First: 1984/10/12]
45. Bush K, Kivlahan DR, McDonell MB, et al. The AUDIT alcohol consumption questions (AUDIT-C): an effective brief screening test for problem drinking. Ambulatory Care Quality Improvement Project (ACQUIP). Alcohol Use Disorders Identification Test. *Archives of internal medicine* 1998;158(16):1789-95. [published Online First: 1998/09/17]



For peer review only - http://bmjopen.bmj.com/site/about/guidelines.xhtml

46. Gossop M, Darke S, Griffiths P, et al. The Severity of Dependence Scale (SDS): psychometric properties of the SDS in English and Australian samples of heroin, cocaine and amphetamine users. *Addiction (Abingdon, England)* 1995;90(5):607-14. [published Online First: 1995/05/01]
47. Derogatis LR. BSI 18, Brief Symptom Inventory 18 : administration, scoring and procedures manual. Minneapolis, MN: NCS Pearson, Inc. 2001.
48. Diener E, Emmons RA, Larsen RJ, et al. The Satisfaction With Life Scale. *Journal of personality assessment* 1985;49(1):71-5. doi: 10.1207/s15327752jpa4901_13 [published Online First: 1985/02/01]
49. Rammstedt B, John OP. Measuring personality in one minute or less: A 10-item short version of the Big Five Inventory in English and German. *Journal of Research in Personality* 2007;41(1):203-12. doi: https://doi.org/10.1016/j.jrp.2006.02.001
50. Stephenson MT, Hoyle RH, Palmgreen P, et al. Brief measures of sensation seeking for screening and large-scale surveys. *Drug and alcohol dependence* 2003;72(3):279-86. [published Online First: 2003/12/04]
51. Schwarzer R, Jerusalem M. Generalized Self-Efficacy scale. In: Weinman J, Wright S, Johnston M, eds. Measures in health psychology: A user's portfolio Causal and control beliefs. Windsor, England: NFER-NELSON. 1995:35-37.
52. Connor KM, Davidson JR. Development of a new resilience scale: the Connor-Davidson Resilience Scale (CD-RISC). *Depression and anxiety* 2003;18(2):76-82. doi: 10.1002/da.10113 [published Online First: 2003/09/10]
53. MAXQDA [program]. 18.1.1 version. Berlin: VERBI Software GmbH, 2018.
54. NVivo [program]. Victoria, Australia: QSR International 2017.
55. SPSS 22 [program]. New York: IBM, 2013.
56. Minozzi S, Saulle R, De Crescenzo F, et al. Psychosocial interventions for psychostimulant misuse. *The Cochrane database of systematic reviews* 2016;9:Cd011866. doi: 10.1002/14651858.CD011866.pub2 [published Online First: 2016/09/30]

For peer review only



For peer review only - http://bmjopen.bmj.com/site/about/guidelines.xhtml

BMJ Open

# Understanding Pathways to Stimulant Use: a mixed-methods examination of the individual, social and cultural factors shaping illicit stimulant use across Europe (ATTUNE): study protocol

| | |
|---|---|
| Journal: | *BMJ Open* |
| Manuscript ID | bmjopen-2019-029476.R1 |
| Article Type: | Protocol |
| Date Submitted by the Author: | 01-May-2019 |
| Complete List of Authors: | Rosenkranz, Moritz; University Medical Centre Hamburg-Eppendorf, Centre of Interdisciplinary Addiction Research of Hamburg University, Department of Psychiatry<br>O'Donnell, Amy; Newcastle University, Institute of Health and Society<br>Verthein, Uwe; University Medical Centre Hamburg-Eppendorf, Centre of Interdisciplinary Addiction Research of Hamburg University, Department of Psychiatry<br>Zurhold, Heike; University Medical Centre Hamburg-Eppendorf, Centre of Interdisciplinary Addiction Research of Hamburg University, Department of Psychiatry<br>Addison, Michelle; Northumbria University, Department of Social Sciences<br>Liebregts, Nienke; University of Amsterdam, Bonger Institute of Criminology<br>Rowicka, Magdalena; Institute of Applied Psychology, Maria Grzegorzewska Academy of Special Education<br>Barták, Miroslav; Charles University and General University Hospital in Prague, Department of Addictology, First Faculty of Medicine<br>Petruželka, Benjamin; Charles University and General University Hospital in Prague, Department of Addictology, First Faculty of Medicine<br>Kaner, Eileen; Newcastle University, Institute<br>Martens, Marcus-Sebastian; University Medical Centre Hamburg-Eppendorf, Centre of Interdisciplinary Addiction Research of Hamburg University, Department of Psychiatry |
| <b>Primary Subject Heading</b>: | Addiction |
| Secondary Subject Heading: | Epidemiology, Public health, Sociology, Qualitative research, Mental health |
| Keywords: | study protocol, amphetamine type stimulants, drug use trajectory, life course |
| | |

SCHOLARONE™
Manuscripts

# Understanding Pathways to Stimulant Use: a mixed-methods examination of the individual, social and cultural factors shaping illicit stimulant use across Europe (ATTUNE): study protocol

Moritz Rosenkranz[1], Amy O'Donnell[2], Uwe Verthein[1], Heike Zurhold[1], Michelle Addison[6], Nienke Liebregts[3], Magdalena Rowicka[4], Miroslav Barták[5], Benjamin Petruželka[5], Eileen Kaner[2] & Marcus-Sebastian Martens[1]

Centre of Interdisciplinary Addiction Research of Hamburg University, Department of Psychiatry, University Medical Centre Hamburg-Eppendorf, Hamburg, Germany[1]

Institute of Health and Society, Newcastle University, Newcastle upon Tyne, United Kingdom[2]

Bonger Institute of Criminology, University of Amsterdam, Amsterdam, Netherlands[3]

Institute of Applied Psychology, Maria Grzegorzewska Academy of Special Education, Warsaw, Poland[4]

Department of Addictology, First Faculty of Medicine, Charles University and General University Hospital in Prague, Prague , Czech Republic[5]

Department of Social Sciences, Faculty of Arts, Design and Social Sciences, Northumbria University, Newcastle upon Tyne, United Kingdom[6]

Corresponding author:
Moritz Rosenkranz
Centre of Interdisciplinary Addiction Research of Hamburg University, Department of Psychiatry, University Medical Centre Hamburg-Eppendorf, Martinistr. 52, 20246 Hamburg, Germany.
Tel.: +49 40 42838-6383
E-mail: moritz.rosenkranz@uni-hamburg.de

Word count: 4041

Key words: study protocol, amphetamine type stimulants, drug use trajectory, life course



For peer review only - http://bmjopen.bmj.com/site/about/guidelines.xhtml

## ABSTRACT

### Introduction

Amphetamine Type Stimulants (ATS) including amphetamine, MDMA/”ecstasy”, methamphetamine, synthetic cathinones and “Ritalin” are the second most commonly used illicit drugs globally. Yet, there is little evidence on which factors are associated with the development of different patterns of ATS use over the life course. This study aims to examine which individual, social, and environmental factors shape different pathways and trajectories of ATS consumption. The study will be conducted in five European countries: Germany; the Netherlands; Poland; Czech Republic and the United Kingdom.

### Methods and analysis

We will use a sequential mixed methods study design to investigate the multiple factors (familial, social and occupational situation, critical life events, general risk behaviour, mental and physical health, satisfaction with life) that shape individual ATS use pathways. A systematic literature review will be performed to provide an overview of the current academic literature on the topic. In Module 1, qualitative semi-structured interviews (n=ATS users and non-users) will be conducted to explore individual experiences of, and perspectives on, dynamics of change in stimulant consumption patterns. In Module 2, structured questionnaires (n=2000 ATS users and non-users) will be administered via tablet computers, to validate and enhance the generalisability of the interview findings. Data integration will take place at two key points. First, during the study, where the findings from the first qualitative interviews will inform the design of the structured questionnaire. Second, at the end of the study, where mixed methods data will be brought together to generate an in-depth, contextualised understanding of the research topic.

### Ethics and dissemination

The study has been approved by the respective responsible ethics committee in each participating country. Data will be treated confidentially to ensure participants’ anonymity. Findings will be disseminated in peer reviewed scientific journals, national and international conferences, and in briefings for policy and practice.


For peer review only

## SUMMARY

### Strengths and limitations of this study

- First study of its kind to examine different pathways of ATS use, and will thus expand an underdeveloped evidence base.
- Large qualitative and quantitative dataset collected in five European countries, which allows valuable inter-country comparisons.
- Applies a theory-based analytical framework to understand the individual, social and environmental influences shaping ATS use, which will provide important insights expedient for the development of tailored prevention and intervention measures.



For peer review only - http://bmjopen.bmj.com/site/about/guidelines.xhtml

- Cross-sectional design allows correlative but no causal conclusions.

For peer review only



For peer review only - http://bmjopen.bmj.com/site/about/guidelines.xhtml

## INTRODUCTION

Amphetamine Type Stimulants (ATS) are the second most commonly used illicit drugs globally[1] as well as in Europe[2]. ATS include amphetamines ("speed"), Methylenedioxymethamphetamine ("MDMA" or "ecstasy"), methamphetamine ("crystal meth")[3] and illicit use of amphetamine-type prescription drugs (e.g. "Ritalin®"). In recent years, there has also been a rise in new psychoactive substances that mimic the effects of stimulants in global drug markets[4], including synthetic cathinones such as mephedrone ("bath salts"). Across Europe as a whole, 0.5% of those aged 15-64 reported using amphetamines in the past 12 months, with higher rates for MDMA use (0.8%). However, ATS use rates vary by country, with the highest consumption found in the Netherlands (amphetamine, 1.7%; MDMA, 3.6%). Data also suggest higher consumption rates for adolescents and younger adults compared to the general population, with one percent of those aged 15-34 reporting amphetamine consumption, and 1.8% MDMA consumption, over the previous year[1 2]. Worldwide, quantities of ATS seized have doubled over the past decade[5], with increased usage levels reflected in wastewater analyses conducted in several European countries[6], and in a corresponding rise in numbers of first-time entrants for stimulant treatment across Europe, from about 7000 in 2006 to more than 12000 in 2016[2]. Long term use of ATS can lead to a substance use disorder, including (psychological) dependency[7]. The global prevalence of substance use disorders related to amphetamines was estimated at nearly five million people in 2016. In Europe around 260.000 people are affected by amphetamine use disorder, with prevalence twice as high in Eastern Europe as in the central region.[8]

Methamphetamine use at dependent levels is associated with multiple co-morbidities, including HIV infection, hepatitis, cardiac effects, cognitive dysfunction, and prominent psychiatric consequences such as psychosis[9 10]. Although MDMA is often viewed as a recreational drug, prolonged use is associated with neurological dysfunction and depression[11]. Additional societal costs identified with ATS abuse include premature death, crime, lost productivity, environmental damage, disruption of family life, and infectious disease[9 12 13]. However, despite the substantial harms associated with ATS use, increased prevalence in consumption rates, and rising number of treatment entries, there is little evidence regarding which factors shape different patterns of ATS use.

Some qualitative and very few quantitative studies exploring influences on ATS use have been published, primarily focussed on factors affecting initiation[14]. Important motives for the initiation of ATS consumption identified in previous research include: curiosity or propensity for experimentation[15-18]; self-management of stress, trauma, or other mental health issues[16 18-24]; and to boost of performance at work/studies[19 21 25-27] or in private settings (sexual relationships, endurance at dance events)[18 26 28-30]. There is some evidence to suggest that continued and/or increased ATS consumption is often to support specific functional needs (improvement of stress management or reduced insecurity in social situations) and to help manage withdrawal effects[15 20 22 27 29 31-34]. Experiencing critical life events (separation, death of a close friend or family member, domestic violence) also



For peer review only - http://bmjopen.bmj.com/site/about/guidelines.xhtml

appears to be associated with sustained use[19 26 32]. Reported factors connected with a decrease (and in some cases, until abstinence) included an increased perception of negative health impacts[16 20 29 35 36], changing social networks, and reduced availability of ATS[28 37 38]. However, there remains limited understanding of what influences different trajectories of consumption over time, and whether this varies by type of ATS substance or user characteristics, such as gender, age or socio-economic status.

In the framework of the European Research Area Network on Illicit Drugs (ERANID), a consortium of five research institutions from Germany, the United Kingdom, Poland, the Netherlands and the Czech Republic, was formed to conduct a study to respond to this evidence gap. The European ATTUNE study (Understanding P**at**hways to S**t**im**u**la**n**t Us**e**: a mixed-methods examination of the individual, social and cultural factors shaping illicit stimulant use across Europe) is led by Germany, as the principal investigator.

**Research objectives**

The overall aim of the project is to improve our understanding of which factors shape different pathways of ATS use in Europe. By examining interactions between individual, social and environmental influencing factors, and the overall trajectory of drug use, this study will explore individual motivations to use ATS and describe different patterns of consumption over time. In doing so, the study seeks to identify potential protective factors (e.g. personality traits, social integration) associated with the ability to control, decrease or quit ATS use, as well as risk factors (e.g. critical life events) associated with the escalation of ATS consumption patterns towards problematic use and/or dependence. Further, we aim to explore why some individuals exposed to ATS select not to use these substances, as well as examining the relationship that illicit stimulant users have with other illicit and licit substances. Targeted recruitment of different ATS user groups with regard to frequency, dependency, former or current use will ensure a sample that varies by type and level of ATS use. Details about the different user groups targeted by this study are provided below.

## METHODS AND ANALYSIS

**Study design**

ATTUNE is a sequential, exploratory, mixed-methods study. The design, implementation and interpretation of the study is informed by the biopsychosocial model of substance use[39]. This model suggests that the change process of drug use pathways is influenced by the interaction of three core domains: individual differences, social dynamics and the environmental/cultural setting. The study comprises of three main components.

First, a systematic review of the qualitative and quantitative published literature on which individual, social and environmental influences shape different pathways of ATS use over the life course. The qualitative literature review is completed and published[14]. We searched four databases for peer-reviewed qualitative studies which explored the views of ATS users on which factors have shaped their drug use careers. The search strategy was conducted in



For peer review only - http://bmjopen.bmj.com/site/about/guidelines.xhtml

accordance with the SPIDER tool [40]. Further details can be found in the corresponding publication. The findings from these reviews aim to provide an insight into the existing international literature that examines ATS users' perspectives on why they start, stop, increase, and/or reduce their ATS consumption, whilst also exploring under which circumstances and conditions stimulant users change their consumption patterns.

Next, in Module 1 of the fieldwork, qualitative methods (semi-structured interviews) will be used to explore individual experiences of and perspectives on dynamics of change in stimulant consumption patterns. The topic guide will build in particular on the findings from the review of qualitative literature conducted at the start of the project, as well as the theoretical underpinning for the research (biopsychosocial model of drug use).

Finally, in Module 2, the findings from the qualitative interviews will inform the development of a structured questionnaire in order to validate and enhance the generalisability of the results in a large sample of ATS users and non-users.

The total length of the study is 36 months (September 2016 to August 2019). The estimated duration of the recruitment as well as data collection for Module 1 is seven months, and for Module 2 it will amount to 13 months.

**Participants**

Eligibility criteria

Individuals who have either used or had the opportunity to use ATS are eligible for inclusion in both the qualitative interviews and survey questionnaire. This includes people who have either consumed ATS at least once in their life, or people who have never used ATS but have been exposed to ATS consumption (defined as having been present when family or friends took ATS but refused to consume themselves). To ensure the inclusion of only those who have had the opportunity to experience changes in the trajectory of their ATS use, the participant´s first ATS consumption (or exposure) needs to have taken place at least five years before the interview or survey questionnaire. We also excluded people previously diagnosed with opioid dependence (self-reported) to avoid overlap between pathways to opioid use and pathways to ATS use. Excluding these participants also ensures that our sample is not dominated by former or current opioid users who consume stimulants primarily to complement their opioid use (e.g. in order to get a "kick" while in opioid substitution therapy).

Further inclusion criteria:

- Aged 18 years or older
- Resident in one of the five national sampling regions
- Able to take part in the interview (not psychotic, no severe cognitive impairments or language barriers).



For peer review only - http://bmjopen.bmj.com/site/about/guidelines.xhtml

A screening website will be set up where interested persons can check their eligibility to participate in an interview or survey questionnaire. If a person is screened successfully, a message is displayed inviting the person to participate and offering different possibilities to contact the research team to arrange an interview appointment. A randomly chosen screening ID facilitates the connection of the screening data with the interview whilst maintaining anonymity.

## Study Groups

Participants in both Module 1 and Module 2 will be recruited using convenience sampling (see below). To ensure a sufficient variety of ATS use patterns or ATS use trajectories, six study groups were predefined for Module 1 and five study groups for Module 2. This approach provides a stratified sample, in which each ATS use pattern (group) serves as a stratum.

### *Module 1: Qualitative semi-structured interviews*

Eligible participants meeting the above criteria will be allocated to one of six study groups depending on their ATS consumption patterns (time, frequency, dependency). Table 1 shows an overview of the operationalisation of the six groups.

We defined current use as ATS consumption within the previous 12 month period. Frequent use was defined as those reporting ATS consumption on ten or more occasions (consumption days) during the previous 12 months (Groups 1 and 3) or any twelve month period prior to the past year (Groups 2 and 4). ATS dependency was assessed using the Severity of Dependence Scale (SDS)[41]. We chose a cut-off of 4 points or more to identify ATS dependency[42].

**Table 1: Operationalization of Study Groups in Module 1**

| Study groups in Module 1 | Name | past 12 months prevalence | ≥ 10 consumption days within past 12 months | ≥ 10 consumption days within one year (at any time except past 12 months) | currently ATS dependent | formerly ATS dependent |
|---|---|---|---|---|---|---|
| Group 1 | Currently dependent users | yes | yes | n.a. | yes | n.a. |
| Group 2 | Formerly dependent users | n.a. | n.a. | yes | no | yes |
| Group 3 | Currently frequent, non-dependent users | yes | yes | n.a. | no | no |
| Group 4 | Formerly frequent, non-dependent users | no | n.a. | yes | no | no |
| Group 5 | Non-frequent users (currently or formerly) | no | n.a. | no | no | no |
| Group 6 | Exposed non-users | n.a. | n.a. | n.a. | n.a. | n.a. |

### *Module 2: Survey questionnaire*

Eligible participants meeting the above criteria will be allocated to one of five study groups depending on their ATS consumption patterns (time, frequency, dependency). Table 2 shows an overview of the operationalisation of the five groups. In order to allow us to distinguish



For peer review only - http://bmjopen.bmj.com/site/about/guidelines.xhtml

clearly between current and previous use, we defined current use as ATS consumption in the past three months, and former use as no consumption in the past 12 months. This means that participants reporting ATS use over three but less than 12 months previously are excluded.

**Table 2: Operationalization of study groups in Module 2**

| Study groups in Module 1 | Name | past 12 months prevalence | past 3 months prevalence | ≥ 10 consumption days within past 12 months | ≥ 10 consumption days within one year (at any time except past 12 months) |
|---|---|---|---|---|---|
| Group A_1 | Currently frequent users | yes | yes | yes | n.a. |
| Group A_2 | Currently non-frequent users | yes | yes | no | n.a. |
| Group B_1 | Formerly frequent users | no | n.a. | n.a. | yes |
| Group B_2 | Formerly non-frequent users | no | n.a. | n.a. | no |
| Group C | Exposed non-users | n.a. | n.a. | n.a. | n.a. |

## Sample size

*Module 1: Qualitative semi-structured interviews*

45 persons per study group (n=270 for the total sample, see table 3) were considered sufficient for Module 1[43]. As we plan to recruit participants via purposeful sampling[44], we expect to generate data that are rich enough to answer our research questions and inform the questionnaire used in Module 2 of the study.

**Table 3: Sample sizes Module 1 by countries and study groups**

| Country | Partner institution | Data collection regions | Sample sizes | | | | | | |
|---|---|---|---|---|---|---|---|---|---|
| | | | Group 1 | Group 2 | Group 3 | Group 4 | Group 5 | Group 6 | **Total N countries** |
| | | | Currently dependent | Formerly depen-dent | Currently frequent, non-dependent | Formerly frequent, non-dependent | Non-frequent (currently or formerly) | Exposed | |
| Germany | ZIS | Border Region to Czech Republic /metropolitan region of Hamburg | 10 | 10 | 10 | 10 | 10 | 10 | **60** |
| United Kingdom | UNEW | Northern England | 10 | 10 | 10 | 10 | 10 | 10 | **60** |
| Poland | APS | Metropolitan region of Warsaw | 10 | 10 | 10 | 10 | 10 | 10 | **60** |
| Netherlands | RG | Amsterdam/the region of Eindhoven | 10 | 10 | 10 | 10 | 10 | 10 | **60** |
| Czech Republic | OGCR | Border region to Germany | 5 | 5 | 5 | 5 | 5 | 5 | **30** |
| **Total N groups** | | | **45** | **45** | **45** | **45** | **45** | **45** | **270** |

*Module 2 Survey questionnaire*

Statistical analyses within the group of current users (A_1 and A_2) as well as within the group of former users (B_1 and B_2) are planned (see table 2). To facilitate this at country



For peer review only - http://bmjopen.bmj.com/site/about/guidelines.xhtml

level, a group size of n=100 is sufficient to detect statistically significant small to medium effects for continuous distributed variables (d≥.40, α=0.05, power=80%)[45]. The sample sizes of the groups are planned as follows. For both, groups A and B, up to 200 participants in each country will be recruited. This allows us to analyse subgroups, e.g. dependent and non-dependent users in groups A as well as B. As group C consists of non-users only, the analyses will focus on comparison with one of the user groups (A or B), reducing the required size for this group to n=100. The total sample size for Module 2 for all countries will amount to 2000 persons (see table 4). Due to funding restraints, the sample size in the Netherlands and the Czech Republic is smaller, which might impede the production of statistically significant country specific intra-group comparisons.

For peer review only

**Table 4: Sample sizes Module 2 by countries and study groups**

| Country | Partner institution | Data collection regions | Sample sizes | | | | | |
|---|---|---|---|---|---|---|---|---|
| | | | Current ATS user | | Former ATS user | | Non ATS user | Total N countries |
| | | | A_1: Frequent | A_2: Non-frequent | B_1: Frequent | B_2: Non-frequent | C: exposed | |
| Germany | ZIS | Border Region to Czech Republic /metropolitan region of Hamburg | 100 | 100 | 100 | 100 | 100 | **500** |
| United Kingdom | UNEW | Northern England | 100 | 100 | 100 | 100 | 100 | **500** |
| Poland | APS | Metropolitan region of Warsaw | 100 | 100 | 100 | 100 | 100 | **500** |
| Netherlands | RG | Amsterdam/the region of Eindhoven | 50 | 50 | 50 | 50 | 50 | **250** |
| Czech Republic | OGCR | Border region to Germany | 50 | 50 | 50 | 50 | 50 | **250** |
| **Total N groups** | | | **400** | **400** | **400** | **400** | **400** | **2000** |

## Recruitment

Sampling method and recruitment procedure

Participants in both Modules will be recruited by non-probability (convenience) sampling, which is an accepted means of accessing participants from ‘hard-to-reach’ as well as minority populations[46 47]. Pre-identified sampling areas in each participating country (see table) are designed to include participants living in urban as well as rural areas.

Multiple methods and sites will be employed to identify and recruit participants for both Modules. Leaflets and posters containing information about the study, a link to the screening website, and contact details of the research teams, will be printed and distributed in substance use help and treatment facilities, on university black boards, in head shops, bars and nightclubs. Social media and substance use web forums will be used to share and circulate the request for participants. Participants will also be recruited actively at university campuses, drug service facilities and music festivals by directly approaching potential participants. All interested interviewees will be screened for eligibility prior to the interview. If eligible to participate, each respondent will receive a screening-code, and the interview



For peer review only - http://bmjopen.bmj.com/site/about/guidelines.xhtml

can either be conducted straight away, or an appointment can be made for a later date. At the end of each interview participants will be asked to recruit further participants from their social network. This additional snowball sampling approach will be realized by handing over study leaflets, as well as up to three numbered cards containing contact details of the research team. The numbers on the cards will help to track the snowball sampling approach.

## Procedure

### Fieldwork

*Module 1*

Face-to-face semi-structured interviews will be conducted by members of the research team of each participating country. Prior to the interview, all participants will receive an information leaflet containing details about the study, explaining what participation involves: anonymity, confidentiality, the use of data, and data protection rules. The participants will then be invited to complete a verbal consent form, should they wish to participate. The interview will be audio recorded and will last approximately 45 to 60 minutes. On completion of the interview, each participant will receive an incentive (money or vouchers, depending on country). All interviews will be transcribed in full, transferred to appropriate software for analysis, and the audio file deleted.

*Module 2*

The quantitative survey will be conducted with the CAPI method (Computer-assisted personal interviewing). The questionnaire content will first be developed in Microsoft Word. Once finalised, all questions will be programmed using survey software GessQ® to enable administration via password-protected tablet computers. The survey instrument will be translated and piloted in all partner countries and revised as necessary. The latest version of the questionnaire will be hosted on a central server operating in the IT environment of Hamburg University and can be downloaded to the tablets directly as necessary. This server is also the recipient for the data uploads from the tablets.

In each country, trained research assistants will recruit participants and conduct the interviews face-to-face or via video-telephony (Skype®). Show cards containing relevant prompts and additional information (e.g. lists of ATS, answering scales) will support the conduct of the interviews.

### Monitoring and data management

The screening process and fieldwork progress will be monitored using a study-specific coordination database. This database will be populated with screening data from each participant as well as key information regarding the interviews or questionnaires, such as study group, duration of interview, gender distribution, sampling region, and contact with drug help services.



For peer review only - http://bmjopen.bmj.com/site/about/guidelines.xhtml

*Module 1*

The raw qualitative data from Module 1 will be managed, stored and analysed by the respective research teams in each participating country. Initial analyses will be conducted at country level using a common coding framework. Analysed data, including emerging interview themes, will be pooled, and provided to ATTUNE PI (Hamburg University).

*Module 2*

The raw quantitative data will be uploaded continuously to the central data management at the Hamburg University. The raw data will be cleaned, edited and transformed into SPSS datasets, one for each country, as well as one comprehensive dataset covering all five countries.

For peer review only

## Survey instruments

*Module 1: Interview guideline and time sheet*

Two semi-structured interview guides will be used to conduct the in-depth interviews in Module 1: one for the ATS user groups plus an adapted topic guide for the non-user group. The topic guide will be based on key emergent themes from the systematic literature review as well as relevant theoretical considerations (biopsychosocial model of substance use). Participants will be asked about their experiences and their consumption patterns regarding ATS and other licit and illicit drugs. To obtain a detailed understanding of which influences have shaped these ATS use patterns, participants will be asked about drug use motives, effects/consequences, settings and occasions, and how these have changed during the period(s) in life where ATS use took place. The interview will end with questions about the (social) setting of use and its impact and the integration of ATS use into the respondent´s lifestyle. The interview guidelines are provided in the supplementary material. Whilst the use of the interview guide will ensure that all central topics are covered, participants will have the opportunity to discuss additional issues or concerns where relevant. During the interviews, researchers will chart participants substance use over time, including age of onset, frequency and life stage, as well as positive and negative life events (e.g. family/partnership, education/work, illness, treatment, imprisonment).

*Module 2: Quantitative questionnaire*

The quantitative survey questionnaire will include questions based on the key themes emerging from the qualitative interviews, as well as a selection of standardised instruments to assess various substance use, health and psychological factors (see table 5).

**Table 5: Overview of standardised measurement instruments**

| Name | Acronym | Content | Reliability: Cronbach´s α | Validity: sensitivity/specificity (cut-off) |
|---|---|---|---|---|
| International Standard Classification of Education[48] | ISCED | Identification of highest educational level | - | - |
| Subjective social integration[49] | SSI | Subjective assessment of social integration | - | - |
| Subjective social position[49] | SSP | Subjective assessment of social position | - | - |
| CAGE questionnaire[50] | CAGE | Alcohol problems lifetime | 0.8-0.98 | 0.71/0.90 (2) |



For peer review only - http://bmjopen.bmj.com/site/about/guidelines.xhtml

| | | | | |
|---|---|---|---|---|
| Alcohol Use Disorders Identification Test[51] | AUDIT-C | Alcohol problems past year | 0.91 | 0.93/0.66 (4) |
| The Severity of Dependence Scale[41] | SDS | ATS dependency lifetime | 0.81–0.89 | 71.3/77.1 (4) |
| Brief Symptom Inventory-18[52] | BSI-18 | Measurement of somatization, anxiety, depression | 0.87-0.94 | 91.2/92.6 (63) |
| Satisfaction with life scale[53] | SWLS | General life satisfaction | - | - |
| Big Five Inventory[54] | BFI-10 | Assessment of five personality traits | 0.58-0.84 | - |
| Brief Sensation Seeking Scale[55] | BSSS-4 | Measurement of sensation seeking | 0.66 | - |
| Generalized Self-Efficacy scale[56] | GSE | Measurement of self-efficacy | 0.92 | - |
| Connor-Davidson Resilience Scale[57] | CD-RISC-10 | Measurement of resilience | 0.89 | - |

The questionnaire will cover:

a. Socio-demographics: These include sex, age, citizenship, migration background, relationship and children, living situation, educational (ISCED) and occupational situation, and social integration (SSI).
b. Drug use: Detailed assessment of all illicit drugs ever used in life (lifetime prevalence, past 12-month prevalence, past 30 days prevalence, age at first and last use), test for alcohol dependence (CAGE, AUDIT-C) and tobacco smoking status.
c. ATS use: Test for ATS dependence (SDS), injecting drug use and treatment experiences; usual setting of ATS use; patterns, motives and consequences of ATS use including (reasons for) changes.
d. Judicial problems: times and reasons for imprisonment
e. Physical and mental health assessment (BSI-18)
f. Personality assessment (BFI-10, BSSS-4, GSE, CD-RISC-10) and critical life events

For group C (non-users), some questions will be omitted (e.g. questions about ATS use), and replaced with alternative questions focussed on their motives for non-use and exposure situation.

## Planned analyses

*Module 1*

Qualitative data from the semi-structured interviews will be analysed using content analysis, and conducted with appropriate software, such as MAXQDA[58] or NVivo[59]. Partners will develop a common, unified coding system to facilitate comparable findings from the interviews across all partner countries. In addition, the data collected with the timeline and chart on substance use and life events will be merged and analysed systematically. Each partner will conduct initial country level analysis separately, in the respective national language. Once this initial analysis has been completed, each partner will produce a report detailing the country-level findings, in English and following a common template. These reports will then be compiled and synthesised to produce a comprehensive, cross-national analysis of the qualitative interviews.

*Module 2*



For peer review only - http://bmjopen.bmj.com/site/about/guidelines.xhtml

The quantitative data analysis will be conducted using the statistical software package SPSS 22[60]. Descriptive, univariate analyses will be used to describe the socio-demographics, health and personality assessment characteristics of the sample, alongside "consumption careers" (substance use, motives of use, and changes of use patterns). Independent sample t-tests and $\chi^2$-analyses, corrected for multiple testing, will be conducted to compare gender and age characteristics among the ATS user groups. Multivariate approaches (regressions, ANOVAs) will be used to assess relations between different ATS user groups and a wide range of possible factors that might shape the use patterns as well as the change of these patterns. If data permit, we will calculate latent class analyses to identify ATS user groups which characteristics are unknown yet. Analyses will be conducted at country level, as well as across the full European sample.

**Patient and Public Involvement**

Patients and members of the public will be involved in ATTUNE at various stages of the study. Policymakers, European non-governmental organisations and service users helped shape the design and focus of the study prior to obtaining funding. We will hold information/discussion sessions about the study with statutory and non-statutory service providers to acquire their insights into how the findings could potentially impact and shape the everyday lives of their service users. Recruited participants enrolled into the study will be invited to act as 'seeds' for the snowball sampling of additional survey interview subjects. This inclusion of patients/public in this way not only helps with enhanced recruitment, but also enables these participants to share their experiences of taking part with others and to underline the importance of the study to people like themselves. ATS user representatives and public representatives will be actively involved in disseminating the results of the research.

## Ethics and dissemination

Based on the regulations in each participating country, ethical approval was obtained. Participant anonymity will be maintained in both the semi-structured interviews and the survey questionnaire. During the survey, no information will be collected that could link the data to the participant concerns. All participants will be explicitly asked to provide informed consent to taking part in the study and made aware of the data protection rules. Consent will be documented within the interview.

Research findings of ATTUNE will be disseminated in peer reviewed, open access journals as well as at national and international conferences and workshops. Each partner will also deliver reports to their funding institutions under the specific terms of the respective country. Additionally, an accessible report will be drafted and distributed to organisations who express an interest in the study. A specific report that is accessible to substance users will be developed for individuals. We intend to disseminate these findings through social media in order to maximise impact and expand networks of interest.



For peer review only - http://bmjopen.bmj.com/site/about/guidelines.xhtml

## Strengths and limitations

By using multiple methods (systematic literature reviews, qualitative interviews and survey questionnaires), this study will generate in-depth, contextualised evidence in an under-explored field of research. The use of stratified sampling will ensure a sufficient variety of types of ATS users and non-users are included, reflecting different use patterns, current and ex-users, dependent and non-dependent users as well as exposed non-users. In particular, the views and experiences of non-users are rarely reflected in the evidence base, and could help generate novel insights into which factors shape decisions not to consume ATS. The application of standardised interview guides and questionnaires will result in a large comprehensive sample that will allow us to compare ATS use in multiple and varied socio-cultural, political and legal environments across Europe.

A key limitation of the study is the cross-sectional design, which makes it difficult to trace pathways and trajectories of ATS use over time. We address this by only including persons, whose first contact with ATS consumption occurred at least five years ago, meaning they have had the chance to develop different ATS use patterns. However, it is important to stress that a cross-sectional design allows correlative but no causal conclusions. One further issue concerns the convenience sampling approach employed in this study in predefined sampling regions. At the same time, compared to representative general population surveys, our method should ensure increased levels of inclusion of different ATS user types, allowing for more detailed and in-depth insights into different use trajectories. Finally, by defining multiple inclusion criteria as well as precise group strata, we aim to mitigate potential bias that could emerge from this sampling approach.

## Implications for interventions and future policy

The findings from this research will enable policy makers and practitioners to improve existing ATS prevention and intervention programmes, and support the development of new approaches in the future. By examining different types of stimulant users (including ex-users and non-users), information will be generated which will be important for universal prevention (targeting general populations), selective prevention (focussing on vulnerable groups), and indicated prevention (aiming at groups that show early signs of problematic substance use). Furthermore, given the limited long-term efficacy of ATS treatment[61], the findings of this study could support the development of ATS treatment programmes that are more effectively tailored to the needs of specific ATS populations and individual users.



For peer review only - http://bmjopen.bmj.com/site/about/guidelines.xhtml

*Authors' contribution:* MR, MSM, HZ, UV, AOD and MA planned and designed the study. All other authors contributed to the design of the study. UV is the principal investigator; MSM and MR are the overall project managers and coordinators of the study. Country specific study coordination in the United Kingdom is performed by AOD, MA and EK, in the Netherlands by NL, in Czech Republic by MB and BP, in Poland by MRow, and in Germany by MR, MSM, HZ and UV. MR wrote the first draft of the study protocol and led the revisions of the manuscript with substantial critical input from all co-authors. In addition to crucial input as regards content, AOD provided significant help to improve the article´s language. All authors read and approved the final version of the manuscript.

*Acknowledgements:* We thank all policymakers, European non-governmental organisations and service users that helped shape the design and focus of the study.

*Funding:* The superordinate research framework ERANID is funded by the European Union under the 7th Framework Programme. Each project partner receives funding from its national funding bodies:
United Kingdom: National Institute for Health Research (NIHR) Policy Research Programme (project ref. PR-ST-0416-10001); Germany: Bundesministerium für Gesundheit (project ref. ZMVI1-2516DSM222); Czech Republic: Government Council for Drug Policy Coordination; Poland: the National Bureau for Drug Prevention; the Netherlands: ZonMw.

*Competing interests:* Uwe Verthein received a speaker's honoraria and travelling expenses from Mundipharma GmbH. Amy O'Donnell was funded by an NIHR School for Primary Care Research Fellowship between October 2015 and September 2017. All other authors declared not to have competing interests.

*Ethics approval:* In GER, UK, PL, CZ, the study has been reviewed and approved by the respective responsible ethics committee, in NL no ethical approval was required. The respective names of all ethics committees as well as reference numbers are as follows: GER: Ethics Committee of the Hamburg Medical Chamber WF-03/17; UK: National Health Service Health Research Authority North East - Newcastle and North Tyneside 2 Research Ethics Committee 17/NE/0283; PL: Academy of Special Education Ethical Committee 168-2018/2019; CZ: Ethical Committee of the National Monitoring Centre for Drugs and Addiction 180326_EK-NMS.

For peer review only



For peer review only - http://bmjopen.bmj.com/site/about/guidelines.xhtml

## REFERENCES


1. UNODC. United Nations Office On Drugs And Crime. World Drug Report 2018. Vienna: United Nations, 2018.
2. EMCDDA. European Monitoring Centre for Drugs and Drug Addiction. European Drug Report 2018: Trends and Developments. Publications Office of the European Union. Luxembourg: European Monitoring Centre for Drugs and Drug Addiction, 2018.
3. EMCDDA. EMCDDA –Europol joint publications: Amphetamine. Lisbon: European Monitoring Centre for Drugs and Drug Addiction 2011.
4. EMCDDA. European Monitoring Centre for Drugs and Drug Addiction and Eurojust. New psychoactive substances in Europe: Legislation and prosecution. Lisbon: EMCDDA & Eurojust, 2017.
5. UNODC. United Nations Office On Drugs And Crime. World Drug Report 2017. Vienna: United Nations, 2017.
6. EMCDDA. European Monitoring Centre for Drugs and Drug Addiction: Wastewater analysis and drugs: a European multi-city study. Perspectives on Drugs. Lisbon, 2018.
7. Best D, Gross S, Vingoe L, et al. Dangerousness of Drugs: A Guide to the Risks and Harms Associated with Substance Use2003.
8. Degenhardt L, Charlson F, Ferrari A, et al. The global burden of disease attributable to alcohol and drug use in 195 countries and territories, 1990–2016: a systematic analysis for the Global Burden of Disease Study 2016. *The Lancet Psychiatry* 2018;5(12):987-1012. doi: 10.1016/S2215-0366(18)30337-7
9. Gonzales R, Mooney L, Rawson R. The Methamphetamine Problem in the United States. *Annual Review of Public Health* 2010;31(1):385-98. doi: 10.1146/annurev.publhealth.012809.103600
10. Degenhardt L, Degenhardt L, Roxburgh A, et al. The epidemiology of methamphetamine use and harm in Australia. *Drug and Alcohol Review* 2008;27(3):243-52. doi: 10.1080/09595230801950572
11. Kelly BC. Mediating MDMA-Related Harm: Preloading and Post-loading Among Ecstasy-Using Youth. *Journal of Psychoactive Drugs* 2009;41(1):19-26. doi: 10.1080/02791072.2009.10400671
12. Home Office. Psychoactive substances ban 6 months on: almost 500 arrests and first convictions. . Retrieved from https://www.gov.uk/government/news/psychoactive-substances-ban-6-months-on-almost-500-arrests-and-first-convictions, 2016.
13. Addison M, Stockdale K, McGovern R, et al. Exploring the intersections between novel psychoactive substances (NPS) and other substance use in a police custody suite setting in the north east of England. *Drugs: Education, Prevention and Policy* 2017:1-7. doi: 10.1080/09687637.2017.1378620




For peer review only - http://bmjopen.bmj.com/site/about/guidelines.xhtml

14. O'Donnell A, Addison M, Spencer L, et al. Which individual, social and environmental influences shape key phases in the amphetamine type stimulant use trajectory? A systematic narrative review and thematic synthesis of the qualitative literature. *Addiction (Abingdon, England)* 2018 doi: 10.1111/add.14434 [published Online First: 2018/09/04]
15. Duff C, Moore D. Evading and embracing normality: estrangement and ambivalence in the accounts of methamphetamine consumers. *Critical Public Health* 2015;25(4):488-503. doi: 10.1080/09581596.2014.913785
16. Levy KB, O'Grady KE, Wish ED, et al. An in-depth qualitative examination of the ecstasy experience: results of a focus group with ecstasy-using college students. *Substance use & misuse* 2005;40(9-10):1427-41. doi: 10.1081/ja-200066810 [published Online First: 2005/07/29]
17. Van Hout MC, Brennan R. Plant food for thought: A qualitative study of mephedrone use in Ireland. *Drugs: Education, Prevention and Policy* 2011;18(5):371-81. doi: 10.3109/09687637.2010.537713
18. Ding Y, He N, Detels R. Circumstances of initiation into new-type drug use among adults in Shanghai: are there differences by types of first new-type drug used? *Drug and alcohol dependence* 2013;131(3):278-83. doi: 10.1016/j.drugalcdep.2012.12.019 [published Online First: 2013/01/15]
19. Boeri MW, Harbry L, Gibson D. A Qualitative Exploration of Trajectories Among Suburban Users of Methamphetamine. *Journal of ethnographic and qualitative research* 2009;3(3):139-51. [published Online First: 2009/01/01]
20. Fast D, Small W, Wood E, et al. Coming 'down here': young people's reflections on becoming entrenched in a local drug scene. *Social science & medicine (1982)* 2009;69(8):1204-10. doi: 10.1016/j.socscimed.2009.07.024 [published Online First: 2009/08/25]
21. Sherman SG, German D, Sirirojn B, et al. Initiation of methamphetamine use among young Thai drug users: a qualitative study. *The Journal of adolescent health : official publication of the Society for Adolescent Medicine* 2008;42(1):36-42. doi: 10.1016/j.jadohealth.2007.07.002 [published Online First: 2007/12/25]
22. Cheney AM, Newkirk CN, Nekhavhambe VM, et al. Effects of social and spatial contexts on young latinas' methamphetamine use initiation. *Journal of ethnicity in substance abuse* 2018;17(1):32-49. doi: 10.1080/15332640.2017.1362721 [published Online First: 2017/10/17]
23. Smirnov A, Najman JM, Hayatbakhsh R, et al. Young adults' recreational social environment as a predictor of ecstasy use initiation: findings of



For peer review only - http://bmjopen.bmj.com/site/about/guidelines.xhtml

a population-based prospective study. *Addiction (Abingdon, England)* 2013;108(10):1809-17. doi: 10.1111/add.12239 [published Online First: 2013/05/15]

24. Cheng WS, Garfein RS, Semple SJ, et al. Binge use and sex and drug use behaviors among HIV(-), heterosexual methamphetamine users in San Diego. *Substance use & misuse* 2010;45(1-2):116-33. doi: 10.3109/10826080902869620 [published Online First: 2009/12/23]
25. Boeri MW, Sterk CE, Elifson KW. Baby Boomer Drug Users: Career Phases, Social Control, and Social Learning Theory. *Sociological Inquiry* 2006;76(2):264-91. doi: doi:10.1111/j.1475-682X.2006.00154.x
26. Ho HT, Le GM, Dinh TT. Female sex workers who use amphetamine-type stimulants (ATS) in three cities of Vietnam: use and sexual risks related to HIV/AIDS. *Global public health* 2013;8(5):552-69. doi: 10.1080/17441692.2013.790459 [published Online First: 2013/05/18]
27. von Mayrhauser C, Brecht ML, Anglin MD. Use ecology and drug use motivations of methamphetamine users admitted to substance abuse treatment facilities in Los Angeles: an emerging profile. *Journal of addictive diseases* 2002;21(1):45-60. [published Online First: 2002/02/08]
28. Bourne A, Reid D, Hickson F, et al. "Chemsex" and harm reduction need among gay men in South London. *The International journal on drug policy* 2015;26(12):1171-6. doi: 10.1016/j.drugpo.2015.07.013 [published Online First: 2015/08/25]
29. Eiserman JM, Diamond S, Schensul JJ. "Rollin' on E": a qualitative analysis of ecstasy use among inner city adolescents and young adults. *Journal of ethnicity in substance abuse* 2005;4(2):9-38. doi: 10.1300/J233v04n02_02 [published Online First: 2005/11/09]
30. Parsons JT, Kelly BC, Weiser JD. Initiation into methamphetamine use for young gay and bisexual men. *Drug and alcohol dependence* 2007;90(2-3):135-44. doi: 10.1016/j.drugalcdep.2007.02.017 [published Online First: 2007/04/03]
31. Carbone-Lopez K, Owens JG, Miller J. Women's "Storylines" of Methamphetamine Initiation in the Midwest. *Journal of Drug Issues* 2012;42(3):226-46. doi: 10.1177/0022042612456013
32. Kerley KR, Copes H, Griffin OH. Middle-Class Motives for Non-Medical Prescription Stimulant Use among College Students. *Deviant Behavior* 2015;36(7):589-603. doi: 10.1080/01639625.2014.951573
33. Bavarian N, Flay BR, Ketcham PL, et al. Illicit use of prescription stimulants in a college student sample: a theory-guided analysis. *Drug and alcohol dependence* 2013;132(3):665-73. doi:

For peer review only

10.1016/j.drugalcdep.2013.04.024 [published Online First: 2013/05/21]
34. Sterk CE, Theall KP, Elifson KW. Getting into ecstasy: comparing moderate and heavy young adult users. *Journal of psychoactive drugs* 2007;39(2):103-13. doi: 10.1080/02791072.2007.10399869 [published Online First: 2007/08/21]
35. Bungay V, Malchy L, Buxton JA, et al. Life with jib: A snapshot of street youth's use of crystal methamphetamine. *Addiction Research & Theory* 2006;14(3):235-51. doi: 10.1080/16066350500270901
36. German D, Sherman SG, Sirirojn B, et al. Motivations for methamphetamine cessation among young people in northern Thailand. *Addiction (Abingdon, England)* 2006;101(8):1143-52. doi: 10.1111/j.1360-0443.2006.01490.x [published Online First: 2006/07/28]
37. O'Brien AM, Brecht M-L, Casey C. Narratives of Methamphetamine Abuse: A Qualitative Exploration of Social, Psychological, and Emotional Experiences. *Journal of Social Work Practice in the Addictions* 2008;8(3):343-66. doi: 10.1080/15332560802224469
38. Sexton RL, Carlson RG, Leukefeld CG, et al. Trajectories of Methamphetamine Use in the Rural South: A Longitudinal Qualitative Study. *Human Organization* 2008;67(2):181-93.
39. Engel GL. The need for a new medical model: a challenge for biomedicine. *Science (New York, NY)* 1977;196(4286):129-36. [published Online First: 1977/04/08]
40. Cooke A, Smith D, Booth A. Beyond PICO: the SPIDER tool for qualitative evidence synthesis. *Qualitative health research* 2012;22(10):1435-43. doi: 10.1177/1049732312452938 [published Online First: 2012/07/26]
41. Gossop M, Darke S, Griffiths P, et al. The Severity of Dependence Scale (SDS): psychometric properties of the SDS in English and Australian samples of heroin, cocaine and amphetamine users. *Addiction (Abingdon, England)* 1995;90(5):607-14. [published Online First: 1995/05/01]
42. Bruno R, Matthews AJ, Topp L, et al. Can the severity of dependence scale be usefully applied to 'ecstasy'? *Neuropsychobiology* 2009;60(3-4):137-47. doi: 10.1159/000253550 [published Online First: 2009/11/07]
43. Malterud K, Siersma VD, Guassora AD. Sample Size in Qualitative Interview Studies: Guided by Information Power. *Qualitative health research* 2015 doi: 10.1177/1049732315617444 [published Online First: 2015/11/29]
44. Palinkas LA, Horwitz SM, Green CA, et al. Purposeful sampling for qualitative data collection and analysis in mixed method



For peer review only - http://bmjopen.bmj.com/site/about/guidelines.xhtml

implementation research. *Administration and policy in mental health* 2015;42(5):533-44. doi: 10.1007/s10488-013-0528-y
45. Cohen J. Statistical power analysis for the behavioral sciences. 2 ed. Hillsdale, NJ, England: Lawrence Erlbaum Accociates 1988.
46. Etikan I, Musa Sa, Alkassim RS. Comparison of Convenience Sampling and Purposive Sampling. *American Journal of Theoretical and Applied Statistics* 2016;5(1):1-4. doi: 10.11648/j.ajtas.20160501.11
47. Shaghaghi A, Bhopal RS, Sheikh A. Approaches to Recruiting 'Hard-To-Reach' Populations into Research: A Review of the Literature. *Health promotion perspectives* 2011;1(2):86-94. doi: 10.5681/hpp.2011.009
48. UNESCO. International Standard Classification of Education (ISCED) 2011 [Available from: http://uis.unesco.org/en/topic/international-standard-classification-education-isced accessed 05.12.2018.
49. Beste J, Bethmann A, Gundert S. Social Structure and Living Conditions: Material and Social Situation of Unemployment Benefit Recipients. In: Research IfE, ed. IAB Summary Reports. Nuremberg, 2014:8.
50. Ewing JA. Detecting alcoholism. The CAGE questionnaire. *Jama* 1984;252(14):1905-7. [published Online First: 1984/10/12]
51. Bush K, Kivlahan DR, McDonell MB, et al. The AUDIT alcohol consumption questions (AUDIT-C): an effective brief screening test for problem drinking. Ambulatory Care Quality Improvement Project (ACQUIP). Alcohol Use Disorders Identification Test. *Archives of internal medicine* 1998;158(16):1789-95. [published Online First: 1998/09/17]
52. Derogatis LR. BSI 18, Brief Symptom Inventory 18 : administration, scoring and procedures manual. Minneapolis, MN: NCS Pearson, Inc. 2001.
53. Diener E, Emmons RA, Larsen RJ, et al. The Satisfaction With Life Scale. *Journal of personality assessment* 1985;49(1):71-5. doi: 10.1207/s15327752jpa4901_13 [published Online First: 1985/02/01]
54. Rammstedt B, John OP. Measuring personality in one minute or less: A 10-item short version of the Big Five Inventory in English and German. *Journal of Research in Personality* 2007;41(1):203-12. doi: https://doi.org/10.1016/j.jrp.2006.02.001
55. Stephenson MT, Hoyle RH, Palmgreen P, et al. Brief measures of sensation seeking for screening and large-scale surveys. *Drug and alcohol dependence* 2003;72(3):279-86. [published Online First: 2003/12/04]
56. Schwarzer R, Jerusalem M. Generalized Self-Efficacy scale. In: Weinman J, Wright S, Johnston M, eds. Measures in health psychology: A user's portfolio Causal and control beliefs. Windsor, England: NFER-NELSON. 1995:35-37.



For peer review only - http://bmjopen.bmj.com/site/about/guidelines.xhtml

57. Connor KM, Davidson JR. Development of a new resilience scale: the Connor-Davidson Resilience Scale (CD-RISC). *Depression and anxiety* 2003;18(2):76-82. doi: 10.1002/da.10113 [published Online First: 2003/09/10]
58. MAXQDA [program]. 18.1.1 version. Berlin: VERBI Software GmbH, 2018.
59. NVivo [program]. Victoria, Australia: QSR International 2017.
60. SPSS 22 [program]. New York: IBM, 2013.
61. Minozzi S, Saulle R, De Crescenzo F, et al. Psychosocial interventions for psychostimulant misuse. *The Cochrane database of systematic reviews* 2016;9:Cd011866. doi: 10.1002/14651858.CD011866.pub2 [published Online First: 2016/09/30]

For peer review only



For peer review only - http://bmjopen.bmj.com/site/about/guidelines.xhtml

**Use for groups 1-5:**
dependent; remitted; frequent; non-dependent; formerly frequent non-dependent, non-frequent

# Interview Guideline 'A' – Attune

**Interviewer:**

This study is about understanding pathways to stimulants use across Europe. You have agreed to participate in an interview as part of this study because you feel you would identify with one of the following statements, would you agree?

a) Are **currently** using amphetamines (Group 1, 2, 3 and 5)
b) **Stopped** using amphetamines (Group 2, 4 and 5)
c) Never used amphetamines but have had the **opportunity** to in the past (Group 6 – USE Guideline B)

## Introduction

**Tell me a little about yourself and your life now.**

(Prompts – Age, individual situation (job, housing, income, education, health situation, emotional well-being), social life (partnership, family, friends) positive and negative impacts)

## Drugs and alcohol

**What is your experience of drug and alcohol use? [Interviewer use CHART tool** for reporting on substances used in life until today]

(Prompts: *personal usage including: age of first use; type of drugs, frequency and quantity, motivations for use/non-use, levels and routes of use; negative impact (dependence, offending, imprisonment)*

**What effect are you hoping for from use of these substances?**

(Prompts: socialising, coping strategy, behaviour change, health)

**Tell me more about the circumstances of your drug and alcohol use since your first use [**plot significant moments in the **life events CHART**]

*(Prompts: use of drugs/alcohol by others around them; any drug/alcohol offers; individual situation (work, housing, income, health), relationships (family, friends, romantic partners); critical life events (violence, separation from significant other, death, illness, chronic disease)*

**What is your use of drug and alcohol like now? Has this changed over time? [CHART tool for substances]**

(Prompts: *type of substance and frequency of use;*
circumstances for changes (living, employment, education, income, health), lifestyle (leisure), relationships (friends, family, romantic partner, marriage, birth of children)

## Stimulants – Firsts, effects, perceptions

**What is your experience of Amphetamine Type Substances? E.g. amphetamine, ecstasy, MDMA, methamphetamine [**REFER to ATS card and CHART for substances]

(Prompts:, , *personal usage including: age of first use; type of drugs; frequency levels and routes of use; and surrounding circumstances of use/non-use, motivations for use/non-use)*

For peer review only

**Use for groups 1-5:**
dependent; remitted; frequent; non-dependent; formerly frequent non-dependent, non-frequent

**What was life like leading up to Amphetamine Type Stimulants being first used? [**plot significant moments in the **life events CHART]**

(Prompt around family, friendships, relationships, education, employment, and social, offending behaviour, health and emotional well-being, housing, significant events)

**What effect were you hoping for from these Amphetamine Type Stimulants? Why did you want this effect?**

(Prompt: feelings, positive and negative experiences, change inhibitions, focus, control, euphoria)

**What was happening in your life when you continue to use XXX (specify the different ATS the person has discussed)? [**plot significant moments in the **life events CHART]**

(Prompts: *use of ATS by others around them, any ATS offers or availability* people in their social network and their use/non-use of ATS, *individual situation (work, housing, income, health), relationships (family, friends, romantic partners); critical life events (violence, separation from significant other, death, illness, chronic disease)*

**What do you personally think about ATS use?**

(Prompts around positive and negative views- physical and mental health, lifestyle, personal plans, offending, social relations)

## Stimulants – Source, availability, funding

**How would/do you usually get Amphetamine Type Stimulants?**
(Prompt: buy from/share with friends, dealer-user interactions, ease of interaction/introduction to dealer)

**How easy or difficult are they to get? Give examples**

**How do you fund your use? Does funding imply any legal problems?**
(Prompt: employment, welfare, criminal activity, deal, trouble with the police, criminal justice system)

## Stimulants – lifestyle and relationships

**How does your ATS use impact on your relationships with others? Give examples**

**How has your lifestyle changed since you started/stopped using ATS? [**plot significant moments in the **life events CHART]**

(**Prompt:** significant life events (individual, social, lifestyle, health and well-being), increases/decreases in substance use since first usage of ATS; protective or harmful factors (dependence, offending)

**How has your use of ATS changed over time? [Plot CHART for substances]**

(Prompt: *type of substance and frequency of use,* increase in levels, decrease in levels, poly-substance use, changes in routes of administration, context of use

**What was happening at the time of the change in use**? [plot significant moments in the **life events CHART]**

(Prompt:, treatment, other service use, circumstances (living, employment, education, income, health), lifestyle (leisure), relationships (friends, family, romantic partner, marriage, birth of children), significant events

**What is important for your life now?**

For peer review only

**Use for groups 1-5:**
dependent; remitted; frequent; non-dependent; formerly frequent non-dependent, non-frequent

(Prompt around family, marriage, children, romantic partners, friends, education, employment, social, offending behaviour, health and emotional well-being, housing, positive and negative impacts)

**What do you hope for/expect for your life in the next 5-10 years?**

(Prompt: change in substance use (including ATS), change in relationships, health, employment career, lifestyle)

*Thank you for taking part in interview*

(Check have consent form)

For peer review only

**Use for group 6:**
Non-stimulant user

## Interview Guideline 'B' – Attune

**Interviewer:**

This study is about understanding pathways to stimulants use across Europe. You have agreed to participate in an interview as part of this study because you feel you would identify with one of the following statements, would you agree?

a) Are **currently** using amphetamines (Group 1, 2, 3, 5 – use Guideline A)
b) **Stopped** using amphetamines (Group 2, 4 and 5 – use Guideline A)
c) Never used amphetamines but have had the **opportunity** to in the past (Group 6 – USE Guideline B)

### Introduction

**Tell me a little about yourself and your life now.**

(Prompts – Age, individual situation (job, housing, income, education, health situation, emotional well-being), social life (partnership, family, friends) positive and negative impacts)

**What is your experience of drug and alcohol use? [Interviewer use CHART tool** for reporting on substances used in life until today]

(Prompts: *personal usage including: age of first use; type of drugs, frequency and quantity motivations for use/non-use, levels and routes of use; negative impact (dependence, offending, imprisonment)*

**What effect are you hoping for from use of these substances?**

(Prompts: socialising, coping strategy, behaviour change, health)

### Drugs and alcohol

**Tell me more about the circumstances of your drug and alcohol use since your first use [**plot significant moments in the **life events CHART**]

*(Prompts: use of drugs/alcohol by others around them; any drug/alcohol offers; individual situation (work, housing, income, health), relationships (family, friends, romantic partners); critical life events (violence, separation from significant other, death, illness, chronic disease)*

**What is your use of drug and alcohol like now? Has this changed over time? [CHART tool for substances**]

(Prompts: *type of substance and frequency of use;* circumstances (living, employment, education, income, health), lifestyle (leisure), relationships (friends, family, romantic partner, marriage, birth of children)

### Stimulants – Firsts, protective factors, perceptions

**What is your experience with the use Amphetamine Type Substances? E.g. amphetamine, ecstasy, MDMA, methamphetamine in your social network?**

(Prompts: *use of ATS by others around them, any ATS offers or availability)*

For peer review only - http://bmjopen.bmj.com/site/about/guidelines.xhtml

**Use for group 6:**
Non-stimulant user

**What was happening in your life when XXX (specify the different ATS the person has discussed by using the ATS card) was offered to you (ATS substances)? [**plot significant moments in the **life events CHART**]

(Prompt: individual and social circumstances surrounding the non-use health and wellbeing, people in their social network and their use/non-use of ATS, specific life events)

**Are others in your networks continuing to use ATS? How does this use impact on your relationship with them? Give examples**

**(**Prompt: changes, life events, availability)

**What was it like being around others who were using ATS?**

(Prompt: impact on relationships, socialising, networks, and employment)

**Has there ever been a situation where you thought about using ATS? [Plot [**plot significant moments in the **life events CHART**] **What was happening at this time for you?**

(prompt: lifestyle, relationships, networks, employment, significant events)

**How would you explain that you never used these ATS**

(Prompt: costs, availability, opportunity, age, circumstances, health, fear of legal problems, fear of dependence, protective factors such as partner, employment, lifestyle, negative impact on other people)

**What do you think about ATS use? Has that perception changed or stayed the same over time?**

(Prompt around positive and negative views- physical and mental health, lifestyle, personal plans, offending, social relations)

## Stimulants – Source, availability, funding

**Would you know how to buy Amphetamine Type Stimulants?**
(Prompt: buy from/share with friends, dealer-user interactions, ease of interaction/introduction to dealer)

**How easy or difficult do you think they are to get? Give reasons**

## Lifestyle

**What is important for your life now?**

(Prompt around family, marriage, children, romantic partners, friends, education, employment, social, offending behaviour, health and emotional well-being, housing, positive and negative impacts)

**What do you hope for/expect for your life in the next 5-10 years?**

(Prompt: change in substance use (including ATS), change in relationships, health, employment career, lifestyle)

*Thank you for taking part in interview*

(Check have consent form)

For peer review only - http://bmjopen.bmj.com/site/about/guidelines.xhtml

# Standards for Reporting Qualitative Research (SRQR)

O'Brien B.C., Harris, I.B., Beckman, T.J., Reed, D.A., & Cook, D.A. (2014). Standards for reporting qualitative research: a synthesis of recommendations. *Academic Medicine, 89(9)*, 1245-1251.

| No. Topic | Item |
|---|---|
| **Title and abstract** | |
| S1 Title | Concise description of the nature and topic of the study identifying the study as qualitative or indicating the approach (e.g., ethnography, grounded theory) or data collection methods (e.g., interview, focus group) is recommended |
| S2 Abstract | Summary of key elements of the study using the abstract format of the intended publication; typically includes objective, methods, results, and conclusions |
| **Introduction** | |
| S3 Problem formulation | Description and significance of the problem/phenomenon studied; review of relevant theory and empirical work; problem statement |
| S4 Purpose or research question | Purpose of the study and specific objectives or questions |
| **Methods** | |
| S5 Qualitative approach and research paradigm | Qualitative approach (e.g., ethnography, grounded theory, case study, phenomenology, narrative research) and guiding theory if appropriate; identifying the research paradigm (e.g., positivist, constructivist/interpretivist) is also recommended |
| S6 Researcher characteristics and reflexivity | Researchers' characteristics that may influence the research, including personal attributes, qualifications/experience, relationship with participants, assumptions, or presuppositions; potential or actual interaction between researchers' characteristics and the research questions, approach, methods, results, or transferability |
| S7 Context | Setting/site and salient contextual factors; rationale[a] |
| S8 Sampling strategy | How and why research participants, documents, or events were selected; criteria for deciding when no further sampling was necessary (e.g., sampling saturation); rationale[a] |
| S9 Ethical issues pertaining to human subjects | Documentation of approval by an appropriate ethics review board and participant consent, or explanation for lack thereof; other confidentiality and data security issues |
| S10 Data collection methods | Types of data collected; details of data collection procedures including (as appropriate) start and stop dates of data collection and analysis, iterative process, triangulation of sources/methods, and modification of procedures in response to evolving study findings; rationale[a] |
| S11 Data collection instruments and technologies | Description of instruments (e.g., interview guides, questionnaires) and devices (e.g., audio recorders) used for data collection; if/how the instrument(s) changed over the course of the study |

For peer review only

| | |
|---|---|
| S12 Units of study | Number and relevant characteristics of participants, documents, or events included in the study; level of participation (could be reported in results) |
| S13 Data processing | Methods for processing data prior to and during analysis, including transcription, data entry, data management and security, verification of data integrity, data coding, and anonymization/deidentification of excerpts |
| S14 Data analysis | Process by which inferences, themes, etc., were identified and developed, including researchers involved in data analysis; usually references a specific paradigm or approach; rationale[a] |
| S15 Techniques to enhance trustworthiness | Techniques to enhance trustworthiness and credibility of data analysis (e.g., member checking, audit trail, triangulation); rationale[a] |
| **Results/Findings** | |
| S16 Synthesis and interpretation | Main findings (e.g., interpretations, inferences, and themes); might include development of a theory or model, or integration with prior research or theory |
| S17 Links to empirical data | Evidence (e.g., quotes, field notes, text excerpts, photographs) to substantiate analytic findings |
| **Discussion** | |
| S18 Integration with prior work, implications, transferability, and contribution(s) to the field | Short summary of main findings; explanation of how findings and conclusions connect to, support, elaborate on, or challenge conclusions of earlier scholarship; discussion of scope of application/generalizability; identification of unique contribution(s) to scholarship in a discipline or field |
| S19 Limitations | Trustworthiness and limitations of findings |
| **Other** | |
| S20 Conflicts of interest | Potential sources of influence or perceived influence on study conduct and conclusions; how these were managed |
| S21 Funding | Sources of funding and other support; role of funders in data collection, interpretation, and reporting |

[a]The rationale should briefly discuss the justification for choosing that theory, approach, method, or technique rather than other options available, the assumptions and limitations implicit in those choices, and how those choices influence study conclusions and transferability. As appropriate, the rationale for several items might be discussed together.

For peer review only

STROBE Statement—Checklist of items that should be included in reports of ***cross-sectional studies***

| | Item No | Recommendation |
|---|---|---|
| **Title and abstract** | 1 | (*a*) Indicate the study's design with a commonly used term in the title or the abstract |
| | | (*b*) Provide in the abstract an informative and balanced summary of what was done and what was found |
| **Introduction** | | |
| Background/rationale | 2 | Explain the scientific background and rationale for the investigation being reported |
| Objectives | 3 | State specific objectives, including any prespecified hypotheses |
| **Methods** | | |
| Study design | 4 | Present key elements of study design early in the paper |
| Setting | 5 | Describe the setting, locations, and relevant dates, including periods of recruitment, exposure, follow-up, and data collection |
| Participants | 6 | (*a*) Give the eligibility criteria, and the sources and methods of selection of participants |
| Variables | 7 | Clearly define all outcomes, exposures, predictors, potential confounders, and effect modifiers. Give diagnostic criteria, if applicable |
| Data sources/ measurement | 8* | For each variable of interest, give sources of data and details of methods of assessment (measurement). Describe comparability of assessment methods if there is more than one group |
| Bias | 9 | Describe any efforts to address potential sources of bias |
| Study size | 10 | Explain how the study size was arrived at |
| Quantitative variables | 11 | Explain how quantitative variables were handled in the analyses. If applicable, describe which groupings were chosen and why |
| Statistical methods | 12 | (*a*) Describe all statistical methods, including those used to control for confounding |
| | | (*b*) Describe any methods used to examine subgroups and interactions |
| | | (*c*) Explain how missing data were addressed |
| | | (*d*) If applicable, describe analytical methods taking account of sampling strategy |
| | | (*e*) Describe any sensitivity analyses |
| **Results** | | |
| Participants | 13* | (a) Report numbers of individuals at each stage of study—eg numbers potentially eligible, examined for eligibility, confirmed eligible, included in the study, completing follow-up, and analysed |
| | | (b) Give reasons for non-participation at each stage |
| | | (c) Consider use of a flow diagram |
| Descriptive data | 14* | (a) Give characteristics of study participants (eg demographic, clinical, social) and information on exposures and potential confounders |
| | | (b) Indicate number of participants with missing data for each variable of interest |
| Outcome data | 15* | Report numbers of outcome events or summary measures |
| Main results | 16 | (*a*) Give unadjusted estimates and, if applicable, confounder-adjusted estimates and their precision (eg, 95% confidence interval). Make clear which confounders were adjusted for and why they were included |
| | | (*b*) Report category boundaries when continuous variables were categorized |
| | | (*c*) If relevant, consider translating estimates of relative risk into absolute risk for a meaningful time period |
| Other analyses | 17 | Report other analyses done—eg analyses of subgroups and interactions, and sensitivity analyses |

For peer review only

| | | |
|---|---|---|
| **Discussion** | | |
| Key results | 18 | Summarise key results with reference to study objectives |
| Limitations | 19 | Discuss limitations of the study, taking into account sources of potential bias or imprecision. Discuss both direction and magnitude of any potential bias |
| Interpretation | 20 | Give a cautious overall interpretation of results considering objectives, limitations, multiplicity of analyses, results from similar studies, and other relevant evidence |
| Generalisability | 21 | Discuss the generalisability (external validity) of the study results |
| **Other information** | | |
| Funding | 22 | Give the source of funding and the role of the funders for the present study and, if applicable, for the original study on which the present article is based |

*Give information separately for exposed and unexposed groups.

**Note:** An Explanation and Elaboration article discusses each checklist item and gives methodological background and published examples of transparent reporting. The STROBE checklist is best used in conjunction with this article (freely available on the Web sites of PLoS Medicine at http://www.plosmedicine.org/, Annals of Internal Medicine at http://www.annals.org/, and Epidemiology at http://www.epidem.com/). Information on the STROBE Initiative is available at www.strobe-statement.org.

For peer review only

BMJ Open

# Understanding Pathways to Stimulant Use: a mixed-methods examination of the individual, social and cultural factors shaping illicit stimulant use across Europe (ATTUNE): study protocol



| | |
|---|---|
| Journal: | *BMJ Open* |
| Manuscript ID | bmjopen-2019-029476.R2 |
| Article Type: | Protocol |
| Date Submitted by the Author: | 14-Jun-2019 |





| | |
|---|---|
| Complete List of Authors: | Rosenkranz, Moritz; University Medical Centre Hamburg-Eppendorf, Centre of Interdisciplinary Addiction Research of Hamburg University, Department of Psychiatry<br>O'Donnell, Amy; Newcastle University, Institute of Health and Society<br>Verthein, Uwe; University Medical Centre Hamburg-Eppendorf, Centre of Interdisciplinary Addiction Research of Hamburg University, Department of Psychiatry<br>Zurhold, Heike; University Medical Centre Hamburg-Eppendorf, Centre of Interdisciplinary Addiction Research of Hamburg University, Department of Psychiatry<br>Addison, Michelle; Northumbria University, Department of Social Sciences<br>Liebregts, Nienke; University of Amsterdam, Bonger Institute of Criminology<br>Rowicka, Magdalena; Institute of Applied Psychology, Maria Grzegorzewska Academy of Special Education<br>Barták, Miroslav; Charles University and General University Hospital in Prague, Department of Addictology, First Faculty of Medicine<br>Petruželka, Benjamin; Charles University and General University Hospital in Prague, Department of Addictology, First Faculty of Medicine<br>Kaner, Eileen; Newcastle University, Institute<br>Martens, Marcus-Sebastian; University Medical Centre Hamburg-Eppendorf, Centre of Interdisciplinary Addiction Research of Hamburg University, Department of Psychiatry |





| | |
|---|---|
| <b>Primary Subject Heading</b>: | Addiction |
| Secondary Subject Heading: | Epidemiology, Public health, Sociology, Qualitative research, Mental health |
| Keywords: | study protocol, amphetamine type stimulants, drug use trajectory, life course |
| | |

# Understanding Pathways to Stimulant Use: a mixed-methods examination of the individual, social and cultural factors shaping illicit stimulant use across Europe (ATTUNE): study protocol

Moritz Rosenkranz[1], Amy O'Donnell[2], Uwe Verthein[1], Heike Zurhold[1], Michelle Addison[6], Nienke Liebregts[3], Magdalena Rowicka[4], Miroslav Barták[5], Benjamin Petruželka[5], Eileen Kaner[2] & Marcus-Sebastian Martens[1]

Centre of Interdisciplinary Addiction Research of Hamburg University, Department of Psychiatry, University Medical Centre Hamburg-Eppendorf, Hamburg, Germany[1]

Institute of Health and Society, Newcastle University, Newcastle upon Tyne, United Kingdom[2]

Bonger Institute of Criminology, University of Amsterdam, Amsterdam, Netherlands[3]

Institute of Applied Psychology, Maria Grzegorzewska Academy of Special Education, Warsaw, Poland[4]

Department of Addictology, First Faculty of Medicine, Charles University and General University Hospital in Prague, Prague , Czech Republic[5]

Department of Social Sciences, Faculty of Arts, Design and Social Sciences, Northumbria University, Newcastle upon Tyne, United Kingdom[6]

Corresponding author:
Moritz Rosenkranz
Centre of Interdisciplinary Addiction Research of Hamburg University, Department of Psychiatry, University Medical Centre Hamburg-Eppendorf, Martinistr. 52, 20246 Hamburg, Germany.
Tel.: +49 40 42838-6383
E-mail: moritz.rosenkranz@uni-hamburg.de

Word count: 4248

Key words: study protocol, amphetamine type stimulants, drug use trajectory, life course



For peer review only - http://bmjopen.bmj.com/site/about/guidelines.xhtml

# ABSTRACT

## Introduction

Amphetamine Type Stimulants (ATS) including amphetamine, MDMA/"ecstasy", methamphetamine, synthetic cathinones and "Ritalin" are the second most commonly used illicit drugs globally. Yet, there is little evidence on which factors are associated with the development of different patterns of ATS use over the life course. This study aims to examine which individual, social, and environmental factors shape different pathways and trajectories of ATS consumption. The study will be conducted in five European countries: Germany; the Netherlands; Poland; Czech Republic and the United Kingdom.

## Methods and analysis

We will use a sequential mixed methods study design to investigate the multiple factors (familial, social and occupational situation, critical life events, general risk behaviour, mental and physical health, satisfaction with life) that shape individual ATS use pathways. A systematic literature review will be performed to provide an overview of the current academic literature on the topic. In Module 1, qualitative semi-structured interviews (n=ATS users and non-users) will be conducted to explore individual experiences of, and perspectives on, dynamics of change in stimulant consumption patterns. In Module 2, structured questionnaires (n=2000 ATS users and non-users) will be administered via tablet computers, to validate and enhance the generalisability of the interview findings. Data integration will take place at two key points. First, during the study, where the findings from the first qualitative interviews will inform the design of the structured questionnaire. Second, at the end of the study, where mixed methods data will be brought together to generate an in-depth, contextualised understanding of the research topic.

## Ethics and dissemination

The study has been approved by the respective responsible ethics committee in each participating country. Data will be treated confidentially to ensure participants' anonymity. Findings will be disseminated in peer reviewed scientific journals, national and international conferences, and in briefings for policy and practice.


# SUMMARY

## Strengths and limitations of this study

- First study of its kind to examine different pathways of ATS use, and will thus expand an underdeveloped evidence base.
- Large qualitative and quantitative dataset collected in five European countries, which allows valuable inter-country comparisons.
- Applies a theory-based analytical framework to understand the individual, social and environmental influences shaping ATS use, which will provide important insights expedient for the development of tailored prevention and intervention measures.



For peer review only - http://bmjopen.bmj.com/site/about/guidelines.xhtml

For peer review only

- Cross-sectional design allows correlative but no causal conclusions.

For peer review only



For peer review only - http://bmjopen.bmj.com/site/about/guidelines.xhtml

# INTRODUCTION

Amphetamine Type Stimulants (ATS) are the second most commonly used illicit drugs globally[1] as well as in Europe[2]. ATS include amphetamines ("speed"), Methylenedioxymethamphetamine ("MDMA" or "ecstasy"), methamphetamine ("crystal meth")[3] and illicit use of amphetamine-type prescription drugs (e.g. "Ritalin®"). In recent years, there has also been a rise in new psychoactive substances that mimic the effects of stimulants in global drug markets[4], including synthetic cathinones such as mephedrone ("bath salts"). Across Europe as a whole, 0.5% of those aged 15-64 reported using amphetamines in the past 12 months, with higher rates for MDMA use (0.8%). However, ATS use rates vary by country, with the highest consumption found in the Netherlands (amphetamine, 1.7%; MDMA, 3.6%). Data also suggest higher consumption rates for adolescents and younger adults compared to the general population, with one percent of those aged 15-34 reporting amphetamine consumption, and 1.8% MDMA consumption, over the previous year[1 2]. Worldwide, quantities of ATS seized have doubled over the past decade[5], with increased usage levels reflected in wastewater analyses conducted in several European countries[6], and in a corresponding rise in numbers of first-time entrants for stimulant treatment across Europe, from about 7000 in 2006 to more than 12000 in 2016[2]. Long term use of ATS can lead to a substance use disorder, including (psychological) dependency[7]. The global prevalence of substance use disorders related to amphetamines was estimated at nearly five million people in 2016. In Europe around 260.000 people are affected by amphetamine use disorder, with prevalence twice as high in Eastern Europe as in the central region.[8]

Methamphetamine use at dependent levels is associated with multiple co-morbidities, including HIV infection, hepatitis, cardiac effects, cognitive dysfunction, and prominent psychiatric consequences such as psychosis[9 10]. Although MDMA is often viewed as a recreational drug, prolonged use is associated with neurological dysfunction and depression[11]. Additional societal costs identified with ATS abuse include premature death, crime, lost productivity, environmental damage, disruption of family life, and infectious disease[9 12 13]. However, despite the substantial harms associated with ATS use, increased prevalence in consumption rates, and rising number of treatment entries, there is little evidence regarding which factors shape different patterns of ATS use.

Some qualitative and very few quantitative studies exploring influences on ATS use have been published, primarily focussed on factors affecting initiation[14]. Important motives for the initiation of ATS consumption identified in previous research include: curiosity or propensity for experimentation[15-18]; self-management of stress, trauma, or other mental health issues[16 18-24]; and to boost of performance at work/studies[19 21 25-27] or in private settings (sexual relationships, endurance at dance events)[18 26 28-30]. There is some evidence to suggest that continued and/or increased ATS consumption is often to support specific functional needs (improvement of stress management or reduced insecurity in social situations) and to help manage withdrawal effects[15 20 22 27 29 31-34]. Experiencing critical life events (separation, death of a close friend or family member, domestic violence) also



For peer review only - http://bmjopen.bmj.com/site/about/guidelines.xhtml

appears to be associated with sustained use[19 26 32]. Reported factors connected with a decrease (and in some cases, until abstinence) included an increased perception of negative health impacts[16 20 29 35 36], changing social networks, and reduced availability of ATS[28 37 38]. However, there remains limited understanding of what influences different trajectories of consumption over time, and whether this varies by type of ATS substance or user characteristics, such as gender, age or socio-economic status.

In the framework of the European Research Area Network on Illicit Drugs (ERANID), a consortium of five research institutions from Germany, the United Kingdom, Poland, the Netherlands and the Czech Republic, was formed to conduct a study to respond to this evidence gap. The European ATTUNE study (Understanding P**at**hways to S**t**im**u**la**n**t Us**e**: a mixed-methods examination of the individual, social and cultural factors shaping illicit stimulant use across Europe) is led by Germany, as the principal investigator.

**Research objectives**

The overall aim of the project is to improve our understanding of which factors shape different pathways of ATS use in Europe. By examining interactions between individual, social and environmental influencing factors, and the overall trajectory of drug use, this study will explore individual motivations to use ATS and describe different patterns of consumption over time. In doing so, the study seeks to identify potential protective factors (e.g. personality traits, social integration) associated with the ability to control, decrease or quit ATS use, as well as risk factors (e.g. critical life events) associated with the escalation of ATS consumption patterns towards problematic use and/or dependence. Further, we aim to explore why some individuals exposed to ATS select not to use these substances, as well as examining the relationship that illicit stimulant users have with other illicit and licit substances. Targeted recruitment of different ATS user groups with regard to frequency, dependency, former or current use will ensure a sample that varies by type and level of ATS use. Details about the different user groups targeted by this study are provided below.

## METHODS AND ANALYSIS

**Study design**

ATTUNE is a sequential, exploratory, mixed-methods study. The design, implementation and interpretation of the study is informed by the biopsychosocial model of substance use[39]. This model suggests that the change process of drug use pathways is influenced by the interaction of three core domains: individual differences, social dynamics and the environmental/cultural setting. The study comprises of three main components.

First, a systematic review of the qualitative and quantitative published literature on which individual, social and environmental influences shape different pathways of ATS use over the life course. The qualitative literature review is completed and published[14]. We searched four databases for peer-reviewed qualitative studies which explored the views of ATS users on which factors have shaped their drug use careers. The search strategy was conducted in



For peer review only - http://bmjopen.bmj.com/site/about/guidelines.xhtml

accordance with the SPIDER tool [40]. Further details can be found in the corresponding publication. The ongoing systematic review of the quantitative literature (studies on ATS use in adults and adolescents) aims to appraise the evidence on risk, protection, resilience, and desistance. Search strategy as well as data extraction are analogous to the qualitative review. The findings from these reviews will provide an insight into the existing international literature that examines ATS users' perspectives on why they start, stop, increase, and/or reduce their ATS consumption, whilst also exploring under which circumstances and conditions stimulant users change their consumption patterns.

Next, in Module 1 of the fieldwork, qualitative methods (semi-structured interviews) will be used to explore individual experiences of and perspectives on dynamics of change in stimulant consumption patterns. The topic guide will build in particular on the findings from the review of qualitative literature conducted at the start of the project, as well as the theoretical underpinning for the research (biopsychosocial model of drug use).

Finally, in Module 2, the findings from the qualitative interviews will inform the development of a structured questionnaire in order to validate and enhance the generalisability of the results in a large sample of ATS users and non-users.

The total length of the study is 36 months (September 2016 to August 2019). The estimated duration of the recruitment as well as data collection for Module 1 is seven months, and for Module 2 it will amount to 13 months.

**Participants**

Eligibility criteria

Individuals who have either used or had the opportunity to use ATS are eligible for inclusion in both the qualitative interviews and survey questionnaire. This includes people who have either consumed ATS at least once in their life, or people who have never used ATS but have been exposed to ATS consumption (defined as having been present when family or friends took ATS but refused to consume themselves). To ensure the inclusion of only those who have had the opportunity to experience changes in the trajectory of their ATS use, the participant´s first ATS consumption (or exposure) needs to have taken place at least five years before the interview or survey questionnaire. We also excluded people previously diagnosed with opioid dependence (self-reported) to avoid overlap between pathways to opioid use and pathways to ATS use. Excluding these participants also ensures that our sample is not dominated by former or current opioid users who consume stimulants primarily to complement their opioid use (e.g. in order to get a "kick" while in opioid substitution therapy).

Further inclusion criteria:

- Aged 18 years or older
- Resident in one of the five national sampling regions



For peer review only - http://bmjopen.bmj.com/site/about/guidelines.xhtml

- Able to take part in the interview (not psychotic, no severe cognitive impairments or language barriers).

A screening website will be set up where interested persons can check their eligibility to participate in an interview or survey questionnaire. If a person is screened successfully, a message is displayed inviting the person to participate and offering different possibilities to contact the research team to arrange an interview appointment. A randomly chosen screening ID facilitates the connection of the screening data with the interview whilst maintaining anonymity.

Study Groups

Participants in both Module 1 and Module 2 will be recruited using convenience sampling (see below). To ensure a sufficient variety of ATS use patterns or ATS use trajectories, six study groups were predefined for Module 1 and five study groups for Module 2. This approach provides a stratified sample, in which each ATS use pattern (group) serves as a stratum.

*Module 1: Qualitative semi-structured interviews*

Eligible participants meeting the above criteria will be allocated to one of six study groups depending on their ATS consumption patterns (time, frequency, dependency). Table 1 shows an overview of the operationalisation of the six groups.

We defined current use as ATS consumption within the previous 12 month period. Frequent use was defined as those reporting ATS consumption on ten or more occasions (consumption days) during the previous 12 months (Groups 1 and 3) or any twelve month period prior to the past year (Groups 2 and 4). ATS dependency was assessed using the Severity of Dependence Scale (SDS)[41]. We chose a cut-off of 4 points or more to identify ATS dependency[42].

**Table 1: Operationalization of Study Groups in Module 1**

| Study groups in Module 1 | Name | past 12 months prevalence | ≥ 10 consumption days within past 12 months | ≥ 10 consumption days within one year (at any time except past 12 months) | currently ATS dependent | formerly ATS dependent |
|---|---|---|---|---|---|---|
| Group 1 | Currently dependent users | yes | yes | n.a. | yes | n.a. |
| Group 2 | Formerly dependent users | n.a. | n.a. | yes | no | yes |
| Group 3 | Currently frequent, non-dependent users | yes | yes | n.a. | no | no |
| Group 4 | Formerly frequent, non-dependent users | no | n.a. | yes | no | no |
| Group 5 | Non-frequent users (currently or formerly) | no | n.a. | no | no | no |
| Group 6 | Exposed non-users | n.a. | n.a. | n.a. | n.a. | n.a. |

*Module 2: Survey questionnaire*



For peer review only - http://bmjopen.bmj.com/site/about/guidelines.xhtml

Eligible participants meeting the above criteria will be allocated to one of five study groups depending on their ATS consumption patterns (time, frequency, dependency). Table 2 shows an overview of the operationalisation of the five groups. In order to allow us to distinguish clearly between current and previous use, we defined current use as ATS consumption in the past three months, and former use as no consumption in the past 12 months. This means that participants reporting ATS use over three but less than 12 months previously are excluded.

**Table 2: Operationalization of study groups in Module 2**

| Study groups in Module 2 | Name | past 12 months prevalence | past 3 months prevalence | ≥ 10 consumption days within past 12 months | ≥ 10 consumption days within one year (at any time except past 12 months) |
|---|---|---|---|---|---|
| Group A_1 | Currently frequent users | yes | yes | yes | n.a. |
| Group A_2 | Currently non-frequent users | yes | yes | no | n.a. |
| Group B_1 | Formerly frequent users | no | n.a. | n.a. | yes |
| Group B_2 | Formerly non-frequent users | no | n.a. | n.a. | no |
| Group C | Exposed non-users | n.a. | n.a. | n.a. | n.a. |

## Sample size

*Module 1: Qualitative semi-structured interviews*

45 persons per study group (n=270 for the total sample, see table 3) were considered sufficient for Module 1[43]. As we plan to recruit participants via purposeful sampling[44], we expect to generate data that are rich enough to answer our research questions and inform the questionnaire used in Module 2 of the study.

**Table 3: Sample sizes Module 1 by countries and study groups**

| Country | Partner institution | Data collection regions | Sample sizes | | | | | | |
|---|---|---|---|---|---|---|---|---|---|
| | | | Group 1 | Group 2 | Group 3 | Group 4 | Group 5 | Group 6 | **Total N countries** |
| | | | Currently dependent | Formerly depen-dent | Currently frequent, non-dependent | Formerly frequent, non-dependent | Non-frequent (currently or formerly) | Exposed | |
| Germany | ZIS | Border Region to Czech Republic /metropolitan region of Hamburg | 10 | 10 | 10 | 10 | 10 | 10 | **60** |
| United Kingdom | UNEW | Northern England | 10 | 10 | 10 | 10 | 10 | 10 | **60** |
| Poland | APS | Metropolitan region of Warsaw | 10 | 10 | 10 | 10 | 10 | 10 | **60** |
| Netherlands | RG | Amsterdam/the region of Eindhoven | 10 | 10 | 10 | 10 | 10 | 10 | **60** |
| Czech Republic | OGCR | Border region to Germany | 5 | 5 | 5 | 5 | 5 | 5 | **30** |
| **Total N groups** | | | **45** | **45** | **45** | **45** | **45** | **45** | **270** |

*Module 2 Survey questionnaire*



For peer review only - http://bmjopen.bmj.com/site/about/guidelines.xhtml

Statistical analyses within the group of current users (A_1 and A_2) as well as within the group of former users (B_1 and B_2) are planned (see table 2). To facilitate this at country level, a group size of n=100 is sufficient to detect statistically significant small to medium effects for continuous distributed variables (d≥.40, α=0.05, power=80%)[45]. The sample sizes of the groups are planned as follows. For both, groups A and B, up to 200 participants in each country will be recruited. This allows us to analyse subgroups, e.g. dependent and non-dependent users in groups A as well as B. As group C consists of non-users only, the analyses will focus on comparison with one of the user groups (A or B), reducing the required size for this group to n=100. The total sample size for Module 2 for all countries will amount to 2000 persons (see table 4). Due to funding restraints, the sample size in the Netherlands and the Czech Republic is smaller, which might impede the production of statistically significant country specific intra-group comparisons.

**Table 4: Sample sizes Module 2 by countries and study groups**

| Country | Partner institution | Data collection regions | Sample sizes | | | | | |
|---|---|---|---|---|---|---|---|---|
| | | | Current ATS user | | Former ATS user | | Non ATS user | Total N countries |
| | | | A_1: Frequent | A_2: Non-frequent | B_1: Frequent | B_2: Non-frequent | C: exposed | |
| Germany | ZIS | Border Region to Czech Republic /metropolitan region of Hamburg | 100 | 100 | 100 | 100 | 100 | **500** |
| United Kingdom | UNEW | Northern England | 100 | 100 | 100 | 100 | 100 | **500** |
| Poland | APS | Metropolitan region of Warsaw | 100 | 100 | 100 | 100 | 100 | **500** |
| Netherlands | RG | Amsterdam/the region of Eindhoven | 50 | 50 | 50 | 50 | 50 | **250** |
| Czech Republic | OGCR | Border region to Germany | 50 | 50 | 50 | 50 | 50 | **250** |
| **Total N groups** | | | **400** | **400** | **400** | **400** | **400** | **2000** |

## Recruitment

### Sampling method and recruitment procedure

Participants in both Modules will be recruited by non-probability (convenience) sampling, which is an accepted means of accessing participants from 'hard-to-reach' as well as minority populations[46 47]. Pre-identified sampling areas in each participating country (see table) are designed to include participants living in urban as well as rural areas.

Multiple methods and sites will be employed to identify and recruit participants for both Modules. Leaflets and posters containing information about the study, a link to the screening website, and contact details of the research teams, will be printed and distributed in substance use help and treatment facilities, on university black boards, in head shops, bars and nightclubs. Social media and substance use web forums will be used to share and circulate the request for participants. Participants will also be recruited actively at university campuses, drug service facilities and music festivals by directly approaching potential



For peer review only - http://bmjopen.bmj.com/site/about/guidelines.xhtml

participants. All interested interviewees will be screened for eligibility prior to the interview. If eligible to participate, each respondent will receive a screening-code, and the interview can either be conducted straight away, or an appointment can be made for a later date. At the end of each interview participants will be asked to recruit further participants from their social network. This additional snowball sampling approach will be realized by handing over study leaflets, as well as up to three numbered cards containing contact details of the research team. The numbers on the cards will help to track the snowball sampling approach.

## Procedure

### Fieldwork

*Module 1*

Face-to-face semi-structured interviews will be conducted by members of the research team of each participating country. Prior to the interview, all participants will receive an information leaflet containing details about the study, explaining what participation involves: anonymity, confidentiality, the use of data, and data protection rules. The participants will then be invited to complete a verbal consent form, should they wish to participate. The interview will be audio recorded and will last approximately 45 to 60 minutes. On completion of the interview, each participant will receive an incentive (money or vouchers, depending on country). All interviews will be transcribed in full, transferred to appropriate software for analysis, and the audio file deleted.

*Module 2*

The quantitative survey will be conducted with the CAPI method (Computer-assisted personal interviewing). The questionnaire content will first be developed in Microsoft Word. Once finalised, all questions will be programmed using survey software GessQ® to enable administration via password-protected tablet computers. The survey instrument will be translated and piloted in all partner countries and revised as necessary. The latest version of the questionnaire will be hosted on a central server operating in the IT environment of Hamburg University and can be downloaded to the tablets directly as necessary. This server is also the recipient for the data uploads from the tablets.

In each country, trained research assistants will recruit participants and conduct the interviews face-to-face or via video-telephony (Skype®). Show cards containing relevant prompts and additional information (e.g. lists of ATS, answering scales) will support the conduct of the interviews.

### Monitoring and data management

The screening process and fieldwork progress will be monitored using a study-specific coordination database. This database will be populated with screening data from each participant as well as key information regarding the interviews or questionnaires, such as



For peer review only - http://bmjopen.bmj.com/site/about/guidelines.xhtml

study group, duration of interview, gender distribution, sampling region, and contact with drug help services.

*Module 1*

The raw qualitative data from Module 1 will be managed, stored and analysed by the respective research teams in each participating country. Initial analyses will be conducted at country level using a common coding framework. Analysed data, including emerging interview themes, will be pooled, and provided to ATTUNE PI (Hamburg University).

*Module 2*

The raw quantitative data will be uploaded continuously to the central data management at the Hamburg University. The raw data will be cleaned, edited and transformed into SPSS datasets, one for each country, as well as one comprehensive dataset covering all five countries.

## Survey instruments

*Module 1: Interview guideline and time sheet*

Two semi-structured interview guides will be used to conduct the in-depth interviews in Module 1: one for the ATS user groups plus an adapted topic guide for the non-user group. The topic guide will be based on key emergent themes from the systematic literature review as well as relevant theoretical considerations (biopsychosocial model of substance use). Participants will be asked about their experiences and their consumption patterns regarding ATS and other licit and illicit drugs. To obtain a detailed understanding of which influences have shaped these ATS use patterns, participants will be asked about drug use motives, effects/consequences, settings and occasions, and how these have changed during the period(s) in life where ATS use took place. The interview will end with questions about the (social) setting of use and its impact and the integration of ATS use into the respondent´s lifestyle. The interview guidelines are provided in the supplementary material. Whilst the use of the interview guide will ensure that all central topics are covered, participants will have the opportunity to discuss additional issues or concerns where relevant. During the interviews, researchers will chart participants substance use over time, including age of onset, frequency and life stage, as well as positive and negative life events (e.g. family/partnership, education/work, illness, treatment, imprisonment).

*Module 2: Quantitative questionnaire*

The quantitative survey questionnaire will include questions based on the key themes emerging from the qualitative interviews, as well as a selection of standardised instruments to assess various substance use, health and psychological factors (see table 5).

**Table 5: Overview of standardised measurement instruments**

| Name | Acronym | Content | Reliability: Cronbach´s α | Validity: sensitivity/specificity (cut-off) |
|---|---|---|---|---|
| International Standard Classification of Education[48] | ISCED | Identification of highest educational level | - | - |



For peer review only - http://bmjopen.bmj.com/site/about/guidelines.xhtml

| | | | | |
|---|---|---|---|---|
| Subjective social integration[49] | SSI | Subjective assessment of social integration | - | - |
| Subjective social position[49] | SSP | Subjective assessment of social position | - | - |
| CAGE questionnaire[50] | CAGE | Alcohol problems lifetime | 0.8-0.98 | 0.71/0.90 (2) |
| Alcohol Use Disorders Identification Test[51] | AUDIT-C | Alcohol problems past year | 0.91 | 0.93/0.66 (4) |
| The Severity of Dependence Scale[41] | SDS | ATS dependency lifetime | 0.81–0.89 | 71.3/77.1 (4) |
| Brief Symptom Inventory-18[52] | BSI-18 | Measurement of somatization, anxiety, depression | 0.87-0.94 | 91.2/92.6 (63) |
| Satisfaction with life scale[53] | SWLS | General life satisfaction | - | - |
| Big Five Inventory[54] | BFI-10 | Assessment of five personality traits | 0.58-0.84 | - |
| Brief Sensation Seeking Scale[55] | BSSS-4 | Measurement of sensation seeking | 0.66 | - |
| Generalized Self-Efficacy scale[56] | GSE | Measurement of self-efficacy | 0.92 | - |
| Connor-Davidson Resilience Scale[57] | CD-RISC-10 | Measurement of resilience | 0.89 | - |

The questionnaire will cover:

a. Socio-demographics: These include sex, age, citizenship, migration background, relationship and children, living situation, educational (ISCED) and occupational situation, and social integration (SSI).
b. Drug use: Detailed assessment of all illicit drugs ever used in life (lifetime prevalence, past 12-month prevalence, past 30 days prevalence, age at first and last use), test for alcohol dependence (CAGE, AUDIT-C) and tobacco smoking status.
c. ATS use: Test for ATS dependence (SDS), injecting drug use and treatment experiences; usual setting of ATS use; patterns, motives and consequences of ATS use including (reasons for) changes.
d. Judicial problems: times and reasons for imprisonment
e. Physical and mental health assessment (BSI-18)
f. Personality assessment (BFI-10, BSSS-4, GSE, CD-RISC-10) and critical life events

For group C (non-users), some questions will be omitted (e.g. questions about ATS use), and replaced with alternative questions focussed on their motives for non-use and exposure situation.

## Planned analyses

*Module 1*

Qualitative data from the semi-structured interviews will be analysed using content analysis, and conducted with appropriate software, such as MAXQDA[58] or NVivo[59]. Partners will develop a common, unified coding system to facilitate comparable findings from the interviews across all partner countries. In addition, the data collected with the timeline and chart on substance use and life events will be merged and analysed systematically. Each partner will conduct initial country level analysis separately, in the respective national language. Once this initial analysis has been completed, each partner will produce a report detailing the country-level findings, in English and following a common template. These



For peer review only - http://bmjopen.bmj.com/site/about/guidelines.xhtml

For peer review only

reports will then be compiled and synthesised to produce a comprehensive, cross-national analysis of the qualitative interviews.

*Module 2*

The quantitative data analysis will be conducted using the statistical software package SPSS 22[60]. Descriptive, univariate analyses will be used to describe the socio-demographics, health and personality assessment characteristics of the sample, alongside "consumption careers" (substance use, motives of use, and changes of use patterns). Independent sample t-tests and $\chi^2$-analyses, corrected for multiple testing, will be conducted to compare gender and age characteristics among the ATS user groups. Multivariate approaches will be used to assess a wide range of factors derived from the biopsychosocial model regarding their possible influence on ATS use patterns. So, for example, by calculating a MANOVA, we can simultaneously test if an individual factor like resilience, a social factor like social integration, and an environmental factor like ATS availability is associated with the number of ATS consumption days, the number of cannabis consumption days and the mental health condition index. Furthermore, the MANOVA allows us to detect interactions between the dependent variables. When it comes to an exploration of different ATS user groups such as frequent users, non-frequent users and non-users, we will apply a multinomial logistic regression and determine the association between e.g. traits and being a member of one of those groups. If data permit, we will calculate latent class analyses to identify ATS user groups which characteristics are unknown yet. Analyses will be conducted at country level, as well as across the full European sample.

**Patient and Public Involvement**

Patients and members of the public will be involved in ATTUNE at various stages of the study. Policymakers, European non-governmental organisations and service users helped shape the design and focus of the study prior to obtaining funding. We will hold information/discussion sessions about the study with statutory and non-statutory service providers to acquire their insights into how the findings could potentially impact and shape the everyday lives of their service users. Recruited participants enrolled into the study will be invited to act as 'seeds' for the snowball sampling of additional survey interview subjects. This inclusion of patients/public in this way not only helps with enhanced recruitment, but also enables these participants to share their experiences of taking part with others and to underline the importance of the study to people like themselves. ATS user representatives and public representatives will be actively involved in disseminating the results of the research.

## Ethics and dissemination

Based on the regulations in each participating country, ethical approval was obtained. Participant anonymity will be maintained in both the semi-structured interviews and the



For peer review only - http://bmjopen.bmj.com/site/about/guidelines.xhtml

survey questionnaire. During the survey, no information will be collected that could link the data to the participant concerns. All participants will be explicitly asked to provide informed consent to taking part in the study and made aware of the data protection rules. A written informed consent form, signed by the project leader, will be made available to each participant before the interview. Verbal consent in Module 1 will then be asked for, recorded, and documented in the transcription of the interview. The quantitative interview (Module 2) starts with the question, if the participant has read the informed consent form and if she/he is willing to give consent. The information about each participant´s consent will be saved in a dedicated variable of the dataset.

Research findings of ATTUNE will be disseminated in peer reviewed, open access journals as well as at national and international conferences and workshops. Each partner will also deliver reports to their funding institutions under the specific terms of the respective country. Additionally, an accessible report will be drafted and distributed to organisations who express an interest in the study. A specific report that is accessible to substance users will be developed for individuals. We intend to disseminate these findings through social media in order to maximise impact and expand networks of interest.

## Strengths and limitations

By using multiple methods (systematic literature reviews, qualitative interviews and survey questionnaires), this study will generate in-depth, contextualised evidence in an under-explored field of research. The use of stratified sampling will ensure a sufficient variety of types of ATS users and non-users are included, reflecting different use patterns, current and ex-users, dependent and non-dependent users as well as exposed non-users. In particular, the views and experiences of non-users are rarely reflected in the evidence base, and could help generate novel insights into which factors shape decisions not to consume ATS. The application of standardised interview guides and questionnaires will result in a large comprehensive sample that will allow us to compare ATS use in multiple and varied socio-cultural, political and legal environments across Europe.

A key limitation of the study is the cross-sectional design, which makes it difficult to trace pathways and trajectories of ATS use over time. We address this by only including persons, whose first contact with ATS consumption occurred at least five years ago, meaning they have had the chance to develop different ATS use patterns. However, it is important to stress that a cross-sectional design allows correlative but no causal conclusions. One further issue concerns the convenience sampling approach employed in this study in predefined sampling regions. At the same time, compared to representative general population surveys, our method should ensure increased levels of inclusion of different ATS user types, allowing for more detailed and in-depth insights into different use trajectories. Finally, by defining multiple inclusion criteria as well as precise group strata, we aim to mitigate potential bias that could emerge from this sampling approach.



For peer review only - http://bmjopen.bmj.com/site/about/guidelines.xhtml

## Implications for interventions and future policy

The findings from this research will enable policy makers and practitioners to improve existing ATS prevention and intervention programmes, and support the development of new approaches in the future. By examining different types of stimulant users (including ex-users and non-users), information will be generated which will be important for universal prevention (targeting general populations), selective prevention (focussing on vulnerable groups), and indicated prevention (aiming at groups that show early signs of problematic substance use). Furthermore, given the limited long-term efficacy of ATS treatment[61], the findings of this study could support the development of ATS treatment programmes that are more effectively tailored to the needs of specific ATS populations and individual users.

*Authors' contribution:* MR, MSM, HZ, UV, AOD and MA planned and designed the study. All other authors contributed to the design of the study. UV is the principal investigator; MSM and MR are the overall project managers and coordinators of the study. Country specific study coordination in the United Kingdom is performed by AOD, MA and EK, in the Netherlands by NL, in Czech Republic by MB and BP, in Poland by MRow, and in Germany by MR, MSM, HZ and UV. MR wrote the first draft of the study protocol and led the revisions of the manuscript with substantial critical input from all co-authors. In addition to crucial input as regards content, AOD provided significant help to improve the article´s language. All authors read and approved the final version of the manuscript.

*Acknowledgements:* We thank all policymakers, European non-governmental organisations and service users that helped shape the design and focus of the study.

*Funding:* The superordinate research framework ERANID is funded by the European Union under the 7th Framework Programme. Each project partner receives funding from its national funding bodies: United Kingdom: National Institute for Health Research (NIHR) Policy Research Programme (project ref. PR-ST-0416-10001); Germany: Bundesministerium für Gesundheit (project ref. ZMVI1-2516DSM222); Czech Republic: Office of the Government of the Czech Republic (Decision No. 10701635 / 18- OPK) and Charles University (No. PROGRES Q06/LF1); Poland: the National Bureau for Drug Prevention; the Netherlands: ZonMw.

*Competing interests:* Uwe Verthein received a speaker's honoraria and travelling expenses from Mundipharma GmbH. Amy O'Donnell was funded by an NIHR School for Primary Care Research Fellowship between October 2015 and September 2017. All other authors declared not to have competing interests.

*Ethics approval:* In GER, UK, PL, CZ, the study has been reviewed and approved by the respective responsible ethics committee, in NL no ethical approval was required. The respective names of all ethics committees as well as reference numbers are as follows: GER: Ethics Committee of the Hamburg Medical Chamber WF-03/17; UK: National Health Service Health Research Authority North East - Newcastle and North Tyneside 2 Research Ethics Committee 17/NE/0283; PL: Academy of Special Education Ethical Committee 168-



For peer review only - http://bmjopen.bmj.com/site/about/guidelines.xhtml

2018/2019; CZ: Ethical Committee of the National Monitoring Centre for Drugs and Addiction 180326_EK-NMS.

For peer review only



For peer review only - http://bmjopen.bmj.com/site/about/guidelines.xhtml

## REFERENCES

1. UNODC. United Nations Office On Drugs And Crime. World Drug Report 2018. Vienna: United Nations, 2018.
2. EMCDDA. European Monitoring Centre for Drugs and Drug Addiction. European Drug Report 2018: Trends and Developments. Publications Office of the European Union. Luxembourg: European Monitoring Centre for Drugs and Drug Addiction, 2018.
3. EMCDDA. EMCDDA –Europol joint publications: Amphetamine. Lisbon: European Monitoring Centre for Drugs and Drug Addiction 2011.
4. EMCDDA. European Monitoring Centre for Drugs and Drug Addiction and Eurojust. New psychoactive substances in Europe: Legislation and prosecution. Lisbon: EMCDDA & Eurojust, 2017.
5. UNODC. United Nations Office On Drugs And Crime. World Drug Report 2017. Vienna: United Nations, 2017.
6. EMCDDA. European Monitoring Centre for Drugs and Drug Addiction: Wastewater analysis and drugs: a European multi-city study. Perspectives on Drugs. Lisbon, 2018.
7. Best D, Gross S, Vingoe L, et al. Dangerousness of Drugs: A Guide to the Risks and Harms Associated with Substance Use2003.
8. Degenhardt L, Charlson F, Ferrari A, et al. The global burden of disease attributable to alcohol and drug use in 195 countries and territories, 1990–2016: a systematic analysis for the Global Burden of Disease Study 2016. *The Lancet Psychiatry* 2018;5(12):987-1012. doi: 10.1016/S2215-0366(18)30337-7
9. Gonzales R, Mooney L, Rawson R. The Methamphetamine Problem in the United States. *Annual Review of Public Health* 2010;31(1):385-98. doi: 10.1146/annurev.publhealth.012809.103600
10. Degenhardt L, Degenhardt L, Roxburgh A, et al. The epidemiology of methamphetamine use and harm in Australia. *Drug and Alcohol Review* 2008;27(3):243-52. doi: 10.1080/09595230801950572
11. Kelly BC. Mediating MDMA-Related Harm: Preloading and Post-loading Among Ecstasy-Using Youth. *Journal of Psychoactive Drugs* 2009;41(1):19-26. doi: 10.1080/02791072.2009.10400671
12. Home Office. Psychoactive substances ban 6 months on: almost 500 arrests and first convictions. . Retrieved from https://www.gov.uk/government/news/psychoactive-substances-ban-6-months-on-almost-500-arrests-and-first-convictions, 2016.
13. Addison M, Stockdale K, McGovern R, et al. Exploring the intersections between novel psychoactive substances (NPS) and other substance use in a police custody suite setting in the north east of England. *Drugs: Education, Prevention and Policy* 2017:1-7. doi: 10.1080/09687637.2017.1378620



For peer review only - http://bmjopen.bmj.com/site/about/guidelines.xhtml

14. O'Donnell A, Addison M, Spencer L, et al. Which individual, social and environmental influences shape key phases in the amphetamine type stimulant use trajectory? A systematic narrative review and thematic synthesis of the qualitative literature. *Addiction (Abingdon, England)* 2018 doi: 10.1111/add.14434 [published Online First: 2018/09/04]
15. Duff C, Moore D. Evading and embracing normality: estrangement and ambivalence in the accounts of methamphetamine consumers. *Critical Public Health* 2015;25(4):488-503. doi: 10.1080/09581596.2014.913785
16. Levy KB, O'Grady KE, Wish ED, et al. An in-depth qualitative examination of the ecstasy experience: results of a focus group with ecstasy-using college students. *Substance use & misuse* 2005;40(9-10):1427-41. doi: 10.1081/ja-200066810 [published Online First: 2005/07/29]
17. Van Hout MC, Brennan R. Plant food for thought: A qualitative study of mephedrone use in Ireland. *Drugs: Education, Prevention and Policy* 2011;18(5):371-81. doi: 10.3109/09687637.2010.537713
18. Ding Y, He N, Detels R. Circumstances of initiation into new-type drug use among adults in Shanghai: are there differences by types of first new-type drug used? *Drug and alcohol dependence* 2013;131(3):278-83. doi: 10.1016/j.drugalcdep.2012.12.019 [published Online First: 2013/01/15]
19. Boeri MW, Harbry L, Gibson D. A Qualitative Exploration of Trajectories Among Suburban Users of Methamphetamine. *Journal of ethnographic and qualitative research* 2009;3(3):139-51. [published Online First: 2009/01/01]
20. Fast D, Small W, Wood E, et al. Coming 'down here': young people's reflections on becoming entrenched in a local drug scene. *Social science & medicine (1982)* 2009;69(8):1204-10. doi: 10.1016/j.socscimed.2009.07.024 [published Online First: 2009/08/25]
21. Sherman SG, German D, Sirirojn B, et al. Initiation of methamphetamine use among young Thai drug users: a qualitative study. *The Journal of adolescent health : official publication of the Society for Adolescent Medicine* 2008;42(1):36-42. doi: 10.1016/j.jadohealth.2007.07.002 [published Online First: 2007/12/25]
22. Cheney AM, Newkirk CN, Nekhavhambe VM, et al. Effects of social and spatial contexts on young latinas' methamphetamine use initiation. *Journal of ethnicity in substance abuse* 2018;17(1):32-49. doi: 10.1080/15332640.2017.1362721 [published Online First: 2017/10/17]
23. Smirnov A, Najman JM, Hayatbakhsh R, et al. Young adults' recreational social environment as a predictor of ecstasy use initiation: findings of



For peer review only - http://bmjopen.bmj.com/site/about/guidelines.xhtml

a population-based prospective study. *Addiction (Abingdon, England)* 2013;108(10):1809-17. doi: 10.1111/add.12239 [published Online First: 2013/05/15]
24. Cheng WS, Garfein RS, Semple SJ, et al. Binge use and sex and drug use behaviors among HIV(-), heterosexual methamphetamine users in San Diego. *Substance use & misuse* 2010;45(1-2):116-33. doi: 10.3109/10826080902869620 [published Online First: 2009/12/23]
25. Boeri MW, Sterk CE, Elifson KW. Baby Boomer Drug Users: Career Phases, Social Control, and Social Learning Theory. *Sociological Inquiry* 2006;76(2):264-91. doi: doi:10.1111/j.1475-682X.2006.00154.x
26. Ho HT, Le GM, Dinh TT. Female sex workers who use amphetamine-type stimulants (ATS) in three cities of Vietnam: use and sexual risks related to HIV/AIDS. *Global public health* 2013;8(5):552-69. doi: 10.1080/17441692.2013.790459 [published Online First: 2013/05/18]
27. von Mayrhauser C, Brecht ML, Anglin MD. Use ecology and drug use motivations of methamphetamine users admitted to substance abuse treatment facilities in Los Angeles: an emerging profile. *Journal of addictive diseases* 2002;21(1):45-60. [published Online First: 2002/02/08]
28. Bourne A, Reid D, Hickson F, et al. "Chemsex" and harm reduction need among gay men in South London. *The International journal on drug policy* 2015;26(12):1171-6. doi: 10.1016/j.drugpo.2015.07.013 [published Online First: 2015/08/25]
29. Eiserman JM, Diamond S, Schensul JJ. "Rollin' on E": a qualitative analysis of ecstasy use among inner city adolescents and young adults. *Journal of ethnicity in substance abuse* 2005;4(2):9-38. doi: 10.1300/J233v04n02_02 [published Online First: 2005/11/09]
30. Parsons JT, Kelly BC, Weiser JD. Initiation into methamphetamine use for young gay and bisexual men. *Drug and alcohol dependence* 2007;90(2-3):135-44. doi: 10.1016/j.drugalcdep.2007.02.017 [published Online First: 2007/04/03]
31. Carbone-Lopez K, Owens JG, Miller J. Women's "Storylines" of Methamphetamine Initiation in the Midwest. *Journal of Drug Issues* 2012;42(3):226-46. doi: 10.1177/0022042612456013
32. Kerley KR, Copes H, Griffin OH. Middle-Class Motives for Non-Medical Prescription Stimulant Use among College Students. *Deviant Behavior* 2015;36(7):589-603. doi: 10.1080/01639625.2014.951573
33. Bavarian N, Flay BR, Ketcham PL, et al. Illicit use of prescription stimulants in a college student sample: a theory-guided analysis. *Drug and alcohol dependence* 2013;132(3):665-73. doi:

For peer review only



For peer review only - http://bmjopen.bmj.com/site/about/guidelines.xhtml

10.1016/j.drugalcdep.2013.04.024 [published Online First: 2013/05/21]

34. Sterk CE, Theall KP, Elifson KW. Getting into ecstasy: comparing moderate and heavy young adult users. *Journal of psychoactive drugs* 2007;39(2):103-13. doi: 10.1080/02791072.2007.10399869 [published Online First: 2007/08/21]
35. Bungay V, Malchy L, Buxton JA, et al. Life with jib: A snapshot of street youth's use of crystal methamphetamine. *Addiction Research & Theory* 2006;14(3):235-51. doi: 10.1080/16066350500270901
36. German D, Sherman SG, Sirirojn B, et al. Motivations for methamphetamine cessation among young people in northern Thailand. *Addiction (Abingdon, England)* 2006;101(8):1143-52. doi: 10.1111/j.1360-0443.2006.01490.x [published Online First: 2006/07/28]
37. O'Brien AM, Brecht M-L, Casey C. Narratives of Methamphetamine Abuse: A Qualitative Exploration of Social, Psychological, and Emotional Experiences. *Journal of Social Work Practice in the Addictions* 2008;8(3):343-66. doi: 10.1080/15332560802224469
38. Sexton RL, Carlson RG, Leukefeld CG, et al. Trajectories of Methamphetamine Use in the Rural South: A Longitudinal Qualitative Study. *Human Organization* 2008;67(2):181-93.
39. Engel GL. The need for a new medical model: a challenge for biomedicine. *Science (New York, NY)* 1977;196(4286):129-36. [published Online First: 1977/04/08]
40. Cooke A, Smith D, Booth A. Beyond PICO: the SPIDER tool for qualitative evidence synthesis. *Qualitative health research* 2012;22(10):1435-43. doi: 10.1177/1049732312452938 [published Online First: 2012/07/26]
41. Gossop M, Darke S, Griffiths P, et al. The Severity of Dependence Scale (SDS): psychometric properties of the SDS in English and Australian samples of heroin, cocaine and amphetamine users. *Addiction (Abingdon, England)* 1995;90(5):607-14. [published Online First: 1995/05/01]
42. Bruno R, Matthews AJ, Topp L, et al. Can the severity of dependence scale be usefully applied to 'ecstasy'? *Neuropsychobiology* 2009;60(3-4):137-47. doi: 10.1159/000253550 [published Online First: 2009/11/07]
43. Malterud K, Siersma VD, Guassora AD. Sample Size in Qualitative Interview Studies: Guided by Information Power. *Qualitative health research* 2015 doi: 10.1177/1049732315617444 [published Online First: 2015/11/29]
44. Palinkas LA, Horwitz SM, Green CA, et al. Purposeful sampling for qualitative data collection and analysis in mixed method



For peer review only - http://bmjopen.bmj.com/site/about/guidelines.xhtml

implementation research. *Administration and policy in mental health* 2015;42(5):533-44. doi: 10.1007/s10488-013-0528-y
45. Cohen J. Statistical power analysis for the behavioral sciences. 2 ed. Hillsdale, NJ, England: Lawrence Erlbaum Accociates 1988.
46. Etikan I, Musa Sa, Alkassim RS. Comparison of Convenience Sampling and Purposive Sampling. *American Journal of Theoretical and Applied Statistics* 2016;5(1):1-4. doi: 10.11648/j.ajtas.20160501.11
47. Shaghaghi A, Bhopal RS, Sheikh A. Approaches to Recruiting 'Hard-To-Reach' Populations into Research: A Review of the Literature. *Health promotion perspectives* 2011;1(2):86-94. doi: 10.5681/hpp.2011.009
48. UNESCO. International Standard Classification of Education (ISCED) 2011 [Available from: http://uis.unesco.org/en/topic/international-standard-classification-education-isced accessed 05.12.2018.
49. Beste J, Bethmann A, Gundert S. Social Structure and Living Conditions: Material and Social Situation of Unemployment Benefit Recipients. In: Research IfE, ed. IAB Summary Reports. Nuremberg, 2014:8.
50. Ewing JA. Detecting alcoholism. The CAGE questionnaire. *Jama* 1984;252(14):1905-7. [published Online First: 1984/10/12]
51. Bush K, Kivlahan DR, McDonell MB, et al. The AUDIT alcohol consumption questions (AUDIT-C): an effective brief screening test for problem drinking. Ambulatory Care Quality Improvement Project (ACQUIP). Alcohol Use Disorders Identification Test. *Archives of internal medicine* 1998;158(16):1789-95. [published Online First: 1998/09/17]
52. Derogatis LR. BSI 18, Brief Symptom Inventory 18 : administration, scoring and procedures manual. Minneapolis, MN: NCS Pearson, Inc. 2001.
53. Diener E, Emmons RA, Larsen RJ, et al. The Satisfaction With Life Scale. *Journal of personality assessment* 1985;49(1):71-5. doi: 10.1207/s15327752jpa4901_13 [published Online First: 1985/02/01]
54. Rammstedt B, John OP. Measuring personality in one minute or less: A 10-item short version of the Big Five Inventory in English and German. *Journal of Research in Personality* 2007;41(1):203-12. doi: https://doi.org/10.1016/j.jrp.2006.02.001
55. Stephenson MT, Hoyle RH, Palmgreen P, et al. Brief measures of sensation seeking for screening and large-scale surveys. *Drug and alcohol dependence* 2003;72(3):279-86. [published Online First: 2003/12/04]
56. Schwarzer R, Jerusalem M. Generalized Self-Efficacy scale. In: Weinman J, Wright S, Johnston M, eds. Measures in health psychology: A user's portfolio Causal and control beliefs. Windsor, England: NFER-NELSON. 1995:35-37.



For peer review only - http://bmjopen.bmj.com/site/about/guidelines.xhtml

57. Connor KM, Davidson JR. Development of a new resilience scale: the Connor-Davidson Resilience Scale (CD-RISC). *Depression and anxiety* 2003;18(2):76-82. doi: 10.1002/da.10113 [published Online First: 2003/09/10]
58. MAXQDA [program]. 18.1.1 version. Berlin: VERBI Software GmbH, 2018.
59. NVivo [program]. Victoria, Australia: QSR International 2017.
60. SPSS 22 [program]. New York: IBM, 2013.
61. Minozzi S, Saulle R, De Crescenzo F, et al. Psychosocial interventions for psychostimulant misuse. *The Cochrane database of systematic reviews* 2016;9:Cd011866. doi: 10.1002/14651858.CD011866.pub2 [published Online First: 2016/09/30]

For peer review only



For peer review only - http://bmjopen.bmj.com/site/about/guidelines.xhtml

**Use for groups 1-5:**
dependent; remitted; frequent; non-dependent; formerly frequent non-dependent, non-frequent

# Interview Guideline 'A' – Attune

**Interviewer:**

This study is about understanding pathways to stimulants use across Europe. You have agreed to participate in an interview as part of this study because you feel you would identify with one of the following statements, would you agree?

a) Are **currently** using amphetamines (Group 1, 2, 3 and 5)
b) **Stopped** using amphetamines (Group 2, 4 and 5)
c) Never used amphetamines but have had the **opportunity** to in the past (Group 6 – USE Guideline B)

## Introduction

**Tell me a little about yourself and your life now.**

(Prompts – Age, individual situation (job, housing, income, education, health situation, emotional well-being), social life (partnership, family, friends) positive and negative impacts)

## Drugs and alcohol

**What is your experience of drug and alcohol use? [Interviewer use CHART tool** for reporting on substances used in life until today]

(Prompts: *personal usage including: age of first use; type of drugs, frequency and quantity, motivations for use/non-use, levels and routes of use; negative impact (dependence, offending, imprisonment)*

**What effect are you hoping for from use of these substances?**

(Prompts: socialising, coping strategy, behaviour change, health)

**Tell me more about the circumstances of your drug and alcohol use since your first use [**plot significant moments in the **life events CHART**]

*(Prompts: use of drugs/alcohol by others around them; any drug/alcohol offers; individual situation (work, housing, income, health), relationships (family, friends, romantic partners); critical life events (violence, separation from significant other, death, illness, chronic disease)*

**What is your use of drug and alcohol like now? Has this changed over time? [CHART tool for substances]**

(Prompts: *type of substance and frequency of use;*
circumstances for changes (living, employment, education, income, health), lifestyle (leisure), relationships (friends, family, romantic partner, marriage, birth of children)

## Stimulants – Firsts, effects, perceptions

**What is your experience of Amphetamine Type Substances? E.g. amphetamine, ecstasy, MDMA, methamphetamine [**REFER to ATS card and CHART for substances]

(Prompts:, , *personal usage including: age of first use; type of drugs; frequency levels and routes of use; and surrounding circumstances of use/non-use, motivations for use/non-use)*

For peer review only - http://bmjopen.bmj.com/site/about/guidelines.xhtml

**Use for groups 1-5:**
dependent; remitted; frequent; non-dependent; formerly frequent non-dependent, non-frequent

**What was life like leading up to Amphetamine Type Stimulants being first used? [**plot significant moments in the **life events CHART]**

(Prompt around family, friendships, relationships, education, employment, and social, offending behaviour, health and emotional well-being, housing, significant events)

**What effect were you hoping for from these Amphetamine Type Stimulants? Why did you want this effect?**

(Prompt: feelings, positive and negative experiences, change inhibitions, focus, control, euphoria)

**What was happening in your life when you continue to use XXX (specify the different ATS the person has discussed)? [**plot significant moments in the **life events CHART]**

(Prompts: *use of ATS by others around them, any ATS offers or availability* people in their social network and their use/non-use of ATS, *individual situation (work, housing, income, health), relationships (family, friends, romantic partners); critical life events (violence, separation from significant other, death, illness, chronic disease)*

**What do you personally think about ATS use?**

(Prompts around positive and negative views- physical and mental health, lifestyle, personal plans, offending, social relations)

## Stimulants – Source, availability, funding

**How would/do you usually get Amphetamine Type Stimulants?**
(Prompt: buy from/share with friends, dealer-user interactions, ease of interaction/introduction to dealer)

**How easy or difficult are they to get? Give examples**

**How do you fund your use? Does funding imply any legal problems?**
(Prompt: employment, welfare, criminal activity, deal, trouble with the police, criminal justice system)

## Stimulants – lifestyle and relationships

**How does your ATS use impact on your relationships with others? Give examples**

**How has your lifestyle changed since you started/stopped using ATS? [**plot significant moments in the **life events CHART]**

(**Prompt:** significant life events (individual, social, lifestyle, health and well-being), increases/decreases in substance use since first usage of ATS; protective or harmful factors (dependence, offending)

**How has your use of ATS changed over time? [Plot CHART for substances]**

(Prompt: *type of substance and frequency of use,* increase in levels, decrease in levels, poly-substance use, changes in routes of administration, context of use

**What was happening at the time of the change in use**? [plot significant moments in the **life events CHART]**

(Prompt:, treatment, other service use, circumstances (living, employment, education, income, health), lifestyle (leisure), relationships (friends, family, romantic partner, marriage, birth of children), significant events

**What is important for your life now?**

For peer review only - http://bmjopen.bmj.com/site/about/guidelines.xhtml

**Use for groups 1-5:**
dependent; remitted; frequent; non-dependent; formerly frequent non-dependent, non-frequent

(Prompt around family, marriage, children, romantic partners, friends, education, employment, social, offending behaviour, health and emotional well-being, housing, positive and negative impacts)

**What do you hope for/expect for your life in the next 5-10 years?**

(Prompt: change in substance use (including ATS), change in relationships, health, employment career, lifestyle)

*Thank you for taking part in interview*

(Check have consent form)

For peer review only

**Use for group 6:**
Non-stimulant user

# Interview Guideline 'B' – Attune

**Interviewer:**

This study is about understanding pathways to stimulants use across Europe. You have agreed to participate in an interview as part of this study because you feel you would identify with one of the following statements, would you agree?

a) Are **currently** using amphetamines (Group 1, 2, 3, 5 – use Guideline A)
b) **Stopped** using amphetamines (Group 2, 4 and 5 – use Guideline A)
c) Never used amphetamines but have had the **opportunity** to in the past (Group 6 – USE Guideline B)

## Introduction

**Tell me a little about yourself and your life now.**

(Prompts – Age, individual situation (job, housing, income, education, health situation, emotional well-being), social life (partnership, family, friends) positive and negative impacts)

**What is your experience of drug and alcohol use? [Interviewer use CHART tool** for reporting on substances used in life until today]

(Prompts: *personal usage including: age of first use; type of drugs, frequency and quantity motivations for use/non-use, levels and routes of use; negative impact (dependence, offending, imprisonment)*

**What effect are you hoping for from use of these substances?**

(Prompts: socialising, coping strategy, behaviour change, health)

## Drugs and alcohol

**Tell me more about the circumstances of your drug and alcohol use since your first use [**plot significant moments in the **life events CHART**]

*(Prompts: use of drugs/alcohol by others around them; any drug/alcohol offers; individual situation (work, housing, income, health), relationships (family, friends, romantic partners); critical life events (violence, separation from significant other, death, illness, chronic disease)*

**What is your use of drug and alcohol like now? Has this changed over time? [CHART tool for substances**]

(Prompts: *type of substance and frequency of use;* circumstances (living, employment, education, income, health), lifestyle (leisure), relationships (friends, family, romantic partner, marriage, birth of children)

## Stimulants – Firsts, protective factors, perceptions

**What is your experience with the use Amphetamine Type Substances? E.g. amphetamine, ecstasy, MDMA, methamphetamine in your social network?**

(Prompts: *use of ATS by others around them, any ATS offers or availability)*

For peer review only

**Use for group 6:**
Non-stimulant user

**What was happening in your life when XXX (specify the different ATS the person has discussed by using the ATS card) was offered to you (ATS substances)? [**plot significant moments in the **life events CHART**]

(Prompt: individual and social circumstances surrounding the non-use health and wellbeing, people in their social network and their use/non-use of ATS, specific life events)

**Are others in your networks continuing to use ATS? How does this use impact on your relationship with them? Give examples**

**(**Prompt: changes, life events, availability)

**What was it like being around others who were using ATS?**

(Prompt: impact on relationships, socialising, networks, and employment)

**Has there ever been a situation where you thought about using ATS? [Plot [**plot significant moments in the **life events CHART**] **What was happening at this time for you?**

(prompt: lifestyle, relationships, networks, employment, significant events)

**How would you explain that you never used these ATS**

(Prompt: costs, availability, opportunity, age, circumstances, health, fear of legal problems, fear of dependence, protective factors such as partner, employment, lifestyle, negative impact on other people)

**What do you think about ATS use? Has that perception changed or stayed the same over time?**

(Prompt around positive and negative views- physical and mental health, lifestyle, personal plans, offending, social relations)

## Stimulants – Source, availability, funding

**Would you know how to buy Amphetamine Type Stimulants?**
(Prompt: buy from/share with friends, dealer-user interactions, ease of interaction/introduction to dealer)

**How easy or difficult do you think they are to get? Give reasons**

## Lifestyle

**What is important for your life now?**

(Prompt around family, marriage, children, romantic partners, friends, education, employment, social, offending behaviour, health and emotional well-being, housing, positive and negative impacts)

**What do you hope for/expect for your life in the next 5-10 years?**

(Prompt: change in substance use (including ATS), change in relationships, health, employment career, lifestyle)

*Thank you for taking part in interview*

(Check have consent form)

For peer review only

# Standards for Reporting Qualitative Research (SRQR)

O'Brien B.C., Harris, I.B., Beckman, T.J., Reed, D.A., & Cook, D.A. (2014). Standards for reporting qualitative research: a synthesis of recommendations. *Academic Medicine, 89(9)*, 1245-1251.

| No. Topic | Item |
|---|---|
| **Title and abstract** | |
| S1 Title | Concise description of the nature and topic of the study identifying the study as qualitative or indicating the approach (e.g., ethnography, grounded theory) or data collection methods (e.g., interview, focus group) is recommended |
| S2 Abstract | Summary of key elements of the study using the abstract format of the intended publication; typically includes objective, methods, results, and conclusions |
| **Introduction** | |
| S3 Problem formulation | Description and significance of the problem/phenomenon studied; review of relevant theory and empirical work; problem statement |
| S4 Purpose or research question | Purpose of the study and specific objectives or questions |
| **Methods** | |
| S5 Qualitative approach and research paradigm | Qualitative approach (e.g., ethnography, grounded theory, case study, phenomenology, narrative research) and guiding theory if appropriate; identifying the research paradigm (e.g., positivist, constructivist/interpretivist) is also recommended |
| S6 Researcher characteristics and reflexivity | Researchers' characteristics that may influence the research, including personal attributes, qualifications/experience, relationship with participants, assumptions, or presuppositions; potential or actual interaction between researchers' characteristics and the research questions, approach, methods, results, or transferability |
| S7 Context | Setting/site and salient contextual factors; rationale[a] |
| S8 Sampling strategy | How and why research participants, documents, or events were selected; criteria for deciding when no further sampling was necessary (e.g., sampling saturation); rationale[a] |
| S9 Ethical issues pertaining to human subjects | Documentation of approval by an appropriate ethics review board and participant consent, or explanation for lack thereof; other confidentiality and data security issues |
| S10 Data collection methods | Types of data collected; details of data collection procedures including (as appropriate) start and stop dates of data collection and analysis, iterative process, triangulation of sources/methods, and modification of procedures in response to evolving study findings; rationale[a] |
| S11 Data collection instruments and technologies | Description of instruments (e.g., interview guides, questionnaires) and devices (e.g., audio recorders) used for data collection; if/how the instrument(s) changed over the course of the study |

For peer review only

| | |
|---|---|
| S12 Units of study | Number and relevant characteristics of participants, documents, or events included in the study; level of participation (could be reported in results) |
| S13 Data processing | Methods for processing data prior to and during analysis, including transcription, data entry, data management and security, verification of data integrity, data coding, and anonymization/deidentification of excerpts |
| S14 Data analysis | Process by which inferences, themes, etc., were identified and developed, including researchers involved in data analysis; usually references a specific paradigm or approach; rationale[a] |
| S15 Techniques to enhance trustworthiness | Techniques to enhance trustworthiness and credibility of data analysis (e.g., member checking, audit trail, triangulation); rationale[a] |
| **Results/Findings** | |
| S16 Synthesis and interpretation | Main findings (e.g., interpretations, inferences, and themes); might include development of a theory or model, or integration with prior research or theory |
| S17 Links to empirical data | Evidence (e.g., quotes, field notes, text excerpts, photographs) to substantiate analytic findings |
| **Discussion** | |
| S18 Integration with prior work, implications, transferability, and contribution(s) to the field | Short summary of main findings; explanation of how findings and conclusions connect to, support, elaborate on, or challenge conclusions of earlier scholarship; discussion of scope of application/generalizability; identification of unique contribution(s) to scholarship in a discipline or field |
| S19 Limitations | Trustworthiness and limitations of findings |
| **Other** | |
| S20 Conflicts of interest | Potential sources of influence or perceived influence on study conduct and conclusions; how these were managed |
| S21 Funding | Sources of funding and other support; role of funders in data collection, interpretation, and reporting |

[a]The rationale should briefly discuss the justification for choosing that theory, approach, method, or technique rather than other options available, the assumptions and limitations implicit in those choices, and how those choices influence study conclusions and transferability. As appropriate, the rationale for several items might be discussed together.

For peer review only

STROBE Statement—Checklist of items that should be included in reports of ***cross-sectional studies***

| | Item No | Recommendation |
|---|---|---|
| **Title and abstract** | 1 | (*a*) Indicate the study's design with a commonly used term in the title or the abstract |
| | | (*b*) Provide in the abstract an informative and balanced summary of what was done and what was found |
| **Introduction** | | |
| Background/rationale | 2 | Explain the scientific background and rationale for the investigation being reported |
| Objectives | 3 | State specific objectives, including any prespecified hypotheses |
| **Methods** | | |
| Study design | 4 | Present key elements of study design early in the paper |
| Setting | 5 | Describe the setting, locations, and relevant dates, including periods of recruitment, exposure, follow-up, and data collection |
| Participants | 6 | (*a*) Give the eligibility criteria, and the sources and methods of selection of participants |
| Variables | 7 | Clearly define all outcomes, exposures, predictors, potential confounders, and effect modifiers. Give diagnostic criteria, if applicable |
| Data sources/ measurement | 8* | For each variable of interest, give sources of data and details of methods of assessment (measurement). Describe comparability of assessment methods if there is more than one group |
| Bias | 9 | Describe any efforts to address potential sources of bias |
| Study size | 10 | Explain how the study size was arrived at |
| Quantitative variables | 11 | Explain how quantitative variables were handled in the analyses. If applicable, describe which groupings were chosen and why |
| Statistical methods | 12 | (*a*) Describe all statistical methods, including those used to control for confounding |
| | | (*b*) Describe any methods used to examine subgroups and interactions |
| | | (*c*) Explain how missing data were addressed |
| | | (*d*) If applicable, describe analytical methods taking account of sampling strategy |
| | | (*e*) Describe any sensitivity analyses |
| **Results** | | |
| Participants | 13* | (a) Report numbers of individuals at each stage of study—eg numbers potentially eligible, examined for eligibility, confirmed eligible, included in the study, completing follow-up, and analysed |
| | | (b) Give reasons for non-participation at each stage |
| | | (c) Consider use of a flow diagram |
| Descriptive data | 14* | (a) Give characteristics of study participants (eg demographic, clinical, social) and information on exposures and potential confounders |
| | | (b) Indicate number of participants with missing data for each variable of interest |
| Outcome data | 15* | Report numbers of outcome events or summary measures |
| Main results | 16 | (*a*) Give unadjusted estimates and, if applicable, confounder-adjusted estimates and their precision (eg, 95% confidence interval). Make clear which confounders were adjusted for and why they were included |
| | | (*b*) Report category boundaries when continuous variables were categorized |
| | | (*c*) If relevant, consider translating estimates of relative risk into absolute risk for a meaningful time period |
| Other analyses | 17 | Report other analyses done—eg analyses of subgroups and interactions, and sensitivity analyses |

For peer review only



For peer review only - http://bmjopen.bmj.com/site/about/guidelines.xhtml

| | | |
|---|---|---|
| **Discussion** | | |
| Key results | 18 | Summarise key results with reference to study objectives |
| Limitations | 19 | Discuss limitations of the study, taking into account sources of potential bias or imprecision. Discuss both direction and magnitude of any potential bias |
| Interpretation | 20 | Give a cautious overall interpretation of results considering objectives, limitations, multiplicity of analyses, results from similar studies, and other relevant evidence |
| Generalisability | 21 | Discuss the generalisability (external validity) of the study results |
| **Other information** | | |
| Funding | 22 | Give the source of funding and the role of the funders for the present study and, if applicable, for the original study on which the present article is based |

*Give information separately for exposed and unexposed groups.

**Note:** An Explanation and Elaboration article discusses each checklist item and gives methodological background and published examples of transparent reporting. The STROBE checklist is best used in conjunction with this article (freely available on the Web sites of PLoS Medicine at http://www.plosmedicine.org/, Annals of Internal Medicine at http://www.annals.org/, and Epidemiology at http://www.epidem.com/). Information on the STROBE Initiative is available at www.strobe-statement.org.

For peer review only

BMJ Open

# Understanding Pathways to Stimulant Use: a mixed-methods examination of the individual, social and cultural factors shaping illicit stimulant use across Europe (ATTUNE): study protocol

| | |
|---|---|
| Journal: | *BMJ Open* |
| Manuscript ID | bmjopen-2019-029476.R3 |
| Article Type: | Protocol |
| Date Submitted by the Author: | 05-Jul-2019 |
| Complete List of Authors: | Rosenkranz, Moritz; University Medical Centre Hamburg-Eppendorf, Centre of Interdisciplinary Addiction Research of Hamburg University, Department of Psychiatry<br>O'Donnell, Amy; Newcastle University, Institute of Health and Society<br>Verthein, Uwe; University Medical Centre Hamburg-Eppendorf, Centre of Interdisciplinary Addiction Research of Hamburg University, Department of Psychiatry<br>Zurhold, Heike; University Medical Centre Hamburg-Eppendorf, Centre of Interdisciplinary Addiction Research of Hamburg University, Department of Psychiatry<br>Addison, Michelle; Northumbria University, Department of Social Sciences<br>Liebregts, Nienke; University of Amsterdam, Bonger Institute of Criminology<br>Rowicka, Magdalena; Institute of Applied Psychology, Maria Grzegorzewska Academy of Special Education<br>Barták, Miroslav; Charles University and General University Hospital in Prague, Department of Addictology, First Faculty of Medicine<br>Petruželka, Benjamin; Charles University and General University Hospital in Prague, Department of Addictology, First Faculty of Medicine<br>Kaner, Eileen; Newcastle University, Institute<br>Martens, Marcus-Sebastian; University Medical Centre Hamburg-Eppendorf, Centre of Interdisciplinary Addiction Research of Hamburg University, Department of Psychiatry |
| <b>Primary Subject Heading</b>: | Addiction |
| Secondary Subject Heading: | Epidemiology, Public health, Sociology, Qualitative research, Mental health |
| Keywords: | study protocol, amphetamine type stimulants, drug use trajectory, life course |
| | |

SCHOLARONE™
Manuscripts

# Understanding Pathways to Stimulant Use: a mixed-methods examination of the individual, social and cultural factors shaping illicit stimulant use across Europe (ATTUNE): study protocol

Moritz Rosenkranz[1], Amy O'Donnell[2], Uwe Verthein[1], Heike Zurhold[1], Michelle Addison[6], Nienke Liebregts[3], Magdalena Rowicka[4], Miroslav Barták[5], Benjamin Petruželka[5], Eileen Kaner[2] & Marcus-Sebastian Martens[1]

Centre of Interdisciplinary Addiction Research of Hamburg University, Department of Psychiatry, University Medical Centre Hamburg-Eppendorf, Hamburg, Germany[1]

Institute of Health and Society, Newcastle University, Newcastle upon Tyne, United Kingdom[2]

Bonger Institute of Criminology, University of Amsterdam, Amsterdam, Netherlands[3]

Institute of Applied Psychology, Maria Grzegorzewska Academy of Special Education, Warsaw, Poland[4]

Department of Addictology, First Faculty of Medicine, Charles University and General University Hospital in Prague, Prague , Czech Republic[5]

Department of Social Sciences, Faculty of Arts, Design and Social Sciences, Northumbria University, Newcastle upon Tyne, United Kingdom[6]

Corresponding author:
Moritz Rosenkranz
Centre of Interdisciplinary Addiction Research of Hamburg University, Department of Psychiatry, University Medical Centre Hamburg-Eppendorf, Martinistr. 52, 20246 Hamburg, Germany.
Tel.: +49 40 42838-6383
E-mail: moritz.rosenkranz@uni-hamburg.de

Word count: 4248

Key words: study protocol, amphetamine type stimulants, drug use trajectory, life course

For peer review only

## ABSTRACT

### Introduction

Amphetamine Type Stimulants (ATS) including amphetamine, MDMA/"ecstasy", methamphetamine, synthetic cathinones and "Ritalin" are the second most commonly used illicit drugs globally. Yet, there is little evidence on which factors are associated with the development of different patterns of ATS use over the life course. This study aims to examine which individual, social, and environmental factors shape different pathways and trajectories of ATS consumption. The study will be conducted in five European countries: Germany; the Netherlands; Poland; Czech Republic and the United Kingdom.

### Methods and analysis

We will use a sequential mixed methods study design to investigate the multiple factors (familial, social and occupational situation, critical life events, general risk behaviour, mental and physical health, satisfaction with life) that shape individual ATS use pathways. A systematic literature review will be performed to provide an overview of the current academic literature on the topic. In Module 1, qualitative semi-structured interviews (n=ATS users and non-users) will be conducted to explore individual experiences of, and perspectives on, dynamics of change in stimulant consumption patterns. In Module 2, structured questionnaires (n=2000 ATS users and non-users) will be administered via tablet computers, to validate and enhance the generalisability of the interview findings. Data integration will take place at two key points. First, during the study, where the findings from the first qualitative interviews will inform the design of the structured questionnaire. Second, at the end of the study, where mixed methods data will be brought together to generate an in-depth, contextualised understanding of the research topic.

### Ethics and dissemination

The study has been approved by the respective responsible ethics committee in each participating country. Data will be treated confidentially to ensure participants' anonymity. Findings will be disseminated in peer reviewed scientific journals, national and international conferences, and in briefings for policy and practice.


## SUMMARY

### Strengths and limitations of this study

- First study of its kind to examine different pathways of ATS use, and will thus expand an underdeveloped evidence base.
- Large qualitative and quantitative dataset collected in five European countries, which allows valuable inter-country comparisons.
- Applies a theory-based analytical framework to understand the individual, social and environmental influences shaping ATS use, which will provide important insights expedient for the development of tailored prevention and intervention measures.



For peer review only - http://bmjopen.bmj.com/site/about/guidelines.xhtml

- Cross-sectional design allows correlative but no causal conclusions.

For peer review only



For peer review only - http://bmjopen.bmj.com/site/about/guidelines.xhtml

## INTRODUCTION

Amphetamine Type Stimulants (ATS) are the second most commonly used illicit drugs globally[1] as well as in Europe[2]. ATS include amphetamines ("speed"), Methylenedioxymethamphetamine ("MDMA" or "ecstasy"), methamphetamine ("crystal meth")[3] and illicit use of amphetamine-type prescription drugs (e.g. "Ritalin®"). In recent years, there has also been a rise in new psychoactive substances that mimic the effects of stimulants in global drug markets[4], including synthetic cathinones such as mephedrone ("bath salts"). Across Europe as a whole, 0.5% of those aged 15-64 reported using amphetamines in the past 12 months, with higher rates for MDMA use (0.8%). However, ATS use rates vary by country, with the highest consumption found in the Netherlands (amphetamine, 1.7%; MDMA, 3.6%). Data also suggest higher consumption rates for adolescents and younger adults compared to the general population, with one percent of those aged 15-34 reporting amphetamine consumption, and 1.8% MDMA consumption, over the previous year[1 2]. Worldwide, quantities of ATS seized have doubled over the past decade[5], with increased usage levels reflected in wastewater analyses conducted in several European countries[6], and in a corresponding rise in numbers of first-time entrants for stimulant treatment across Europe, from about 7000 in 2006 to more than 12000 in 2016[2]. Long term use of ATS can lead to a substance use disorder, including (psychological) dependency[7]. The global prevalence of substance use disorders related to amphetamines was estimated at nearly five million people in 2016. In Europe around 260.000 people are affected by amphetamine use disorder, with prevalence twice as high in Eastern Europe as in the central region.[8]

Methamphetamine use at dependent levels is associated with multiple co-morbidities, including HIV infection, hepatitis, cardiac effects, cognitive dysfunction, and prominent psychiatric consequences such as psychosis[9 10]. Although MDMA is often viewed as a recreational drug, prolonged use is associated with neurological dysfunction and depression[11]. Additional societal costs identified with ATS abuse include premature death, crime, lost productivity, environmental damage, disruption of family life, and infectious disease[9 12 13]. However, despite the substantial harms associated with ATS use, increased prevalence in consumption rates, and rising number of treatment entries, there is little evidence regarding which factors shape different patterns of ATS use.

Some qualitative and very few quantitative studies exploring influences on ATS use have been published, primarily focussed on factors affecting initiation[14]. Important motives for the initiation of ATS consumption identified in previous research include: curiosity or propensity for experimentation[15-18]; self-management of stress, trauma, or other mental health issues[16 18-24]; and to boost of performance at work/studies[19 21 25-27] or in private settings (sexual relationships, endurance at dance events)[18 26 28-30]. There is some evidence to suggest that continued and/or increased ATS consumption is often to support specific functional needs (improvement of stress management or reduced insecurity in social situations) and to help manage withdrawal effects[15 20 22 27 29 31-34]. Experiencing critical life events (separation, death of a close friend or family member, domestic violence) also



For peer review only - http://bmjopen.bmj.com/site/about/guidelines.xhtml

appears to be associated with sustained use[19 26 32]. Reported factors connected with a decrease (and in some cases, until abstinence) included an increased perception of negative health impacts[16 20 29 35 36], changing social networks, and reduced availability of ATS[28 37 38]. However, there remains limited understanding of what influences different trajectories of consumption over time, and whether this varies by type of ATS substance or user characteristics, such as gender, age or socio-economic status.

In the framework of the European Research Area Network on Illicit Drugs (ERANID), a consortium of five research institutions from Germany, the United Kingdom, Poland, the Netherlands and the Czech Republic, was formed to conduct a study to respond to this evidence gap. The European ATTUNE study (Understanding P**at**hways to S**t**im**u**la**n**t Us**e**: a mixed-methods examination of the individual, social and cultural factors shaping illicit stimulant use across Europe) is led by Germany, as the principal investigator.

**Research objectives**

The overall aim of the project is to improve our understanding of which factors shape different pathways of ATS use in Europe. By examining interactions between individual, social and environmental influencing factors, and the overall trajectory of drug use, this study will explore individual motivations to use ATS and describe different patterns of consumption over time. In doing so, the study seeks to identify potential protective factors (e.g. personality traits, social integration) associated with the ability to control, decrease or quit ATS use, as well as risk factors (e.g. critical life events) associated with the escalation of ATS consumption patterns towards problematic use and/or dependence. Further, we aim to explore why some individuals exposed to ATS select not to use these substances, as well as examining the relationship that illicit stimulant users have with other illicit and licit substances. Targeted recruitment of different ATS user groups with regard to frequency, dependency, former or current use will ensure a sample that varies by type and level of ATS use. Details about the different user groups targeted by this study are provided below.

## METHODS AND ANALYSIS

**Study design**

ATTUNE is a sequential, exploratory, mixed-methods study. The design, implementation and interpretation of the study is informed by the biopsychosocial model of substance use[39]. This model suggests that the change process of drug use pathways is influenced by the interaction of three core domains: individual differences, social dynamics and the environmental/cultural setting. The study comprises of three main components.

First, a systematic review of the qualitative and quantitative published literature on which individual, social and environmental influences shape different pathways of ATS use over the life course. The qualitative literature review is completed and published[14]. We searched four databases for peer-reviewed qualitative studies which explored the views of ATS users on which factors have shaped their drug use careers. The search strategy was conducted in



For peer review only - http://bmjopen.bmj.com/site/about/guidelines.xhtml

accordance with the SPIDER tool [40]. Further details can be found in the corresponding publication. The ongoing systematic review of the quantitative literature (studies on ATS use in adults and adolescents) aims to appraise the evidence on risk, protection, resilience, and desistance. Search strategy as well as data extraction are analogous to the qualitative review. The findings from these reviews will provide an insight into the existing international literature that examines ATS users' perspectives on why they start, stop, increase, and/or reduce their ATS consumption, whilst also exploring under which circumstances and conditions stimulant users change their consumption patterns.

Next, in Module 1 of the fieldwork, qualitative methods (semi-structured interviews) will be used to explore individual experiences of and perspectives on dynamics of change in stimulant consumption patterns. The topic guide will build in particular on the findings from the review of qualitative literature conducted at the start of the project, as well as the theoretical underpinning for the research (biopsychosocial model of drug use).

Finally, in Module 2, the findings from the qualitative interviews will inform the development of a structured questionnaire in order to validate and enhance the generalisability of the results in a large sample of ATS users and non-users.

The total length of the study is 36 months (September 2016 to August 2019). The estimated duration of the recruitment as well as data collection for Module 1 is seven months, and for Module 2 it will amount to 13 months.

**Participants**

Eligibility criteria

Individuals who have either used or had the opportunity to use ATS are eligible for inclusion in both the qualitative interviews and survey questionnaire. This includes people who have either consumed ATS at least once in their life, or people who have never used ATS but have been exposed to ATS consumption (defined as having been present when family or friends took ATS but refused to consume themselves). To ensure the inclusion of only those who have had the opportunity to experience changes in the trajectory of their ATS use, the participant´s first ATS consumption (or exposure) needs to have taken place at least five years before the interview or survey questionnaire. We also excluded people previously diagnosed with opioid dependence (self-reported) to avoid overlap between pathways to opioid use and pathways to ATS use. Excluding these participants also ensures that our sample is not dominated by former or current opioid users who consume stimulants primarily to complement their opioid use (e.g. in order to get a "kick" while in opioid substitution therapy).

Further inclusion criteria:

- Aged 18 years or older
- Resident in one of the five national sampling regions



For peer review only - http://bmjopen.bmj.com/site/about/guidelines.xhtml

- Able to take part in the interview (not psychotic, no severe cognitive impairments or language barriers).

A screening website will be set up where interested persons can check their eligibility to participate in an interview or survey questionnaire. If a person is screened successfully, a message is displayed inviting the person to participate and offering different possibilities to contact the research team to arrange an interview appointment. A randomly chosen screening ID facilitates the connection of the screening data with the interview whilst maintaining anonymity.

Study Groups

Participants in both Module 1 and Module 2 will be recruited using convenience sampling (see below). To ensure a sufficient variety of ATS use patterns or ATS use trajectories, six study groups were predefined for Module 1 and five study groups for Module 2. This approach provides a stratified sample, in which each ATS use pattern (group) serves as a stratum.

*Module 1: Qualitative semi-structured interviews*

Eligible participants meeting the above criteria will be allocated to one of six study groups depending on their ATS consumption patterns (time, frequency, dependency). Table 1 shows an overview of the operationalisation of the six groups.

We defined current use as ATS consumption within the previous 12 month period. Frequent use was defined as those reporting ATS consumption on ten or more occasions (consumption days) during the previous 12 months (Groups 1 and 3) or any twelve month period prior to the past year (Groups 2 and 4). ATS dependency was assessed using the Severity of Dependence Scale (SDS)[41]. We chose a cut-off of 4 points or more to identify ATS dependency[42].

**Table 1: Operationalization of Study Groups in Module 1**

| **Study groups in Module 1** | **Name** | **past 12 months prevalence** | **≥ 10 consumption days within past 12 months** | **≥ 10 consumption days within one year (at any time except past 12 months)** | **currently ATS dependent** | **formerly ATS dependent** |
|---|---|---|---|---|---|---|
| Group 1 | Currently dependent users | yes | yes | n.a. | yes | n.a. |
| Group 2 | Formerly dependent users | n.a. | n.a. | yes | no | yes |
| Group 3 | Currently frequent, non-dependent users | yes | yes | n.a. | no | no |
| Group 4 | Formerly frequent, non-dependent users | no | n.a. | yes | no | no |
| Group 5 | Non-frequent users (currently or formerly) | no | n.a. | no | no | no |
| Group 6 | Exposed non-users | n.a. | n.a. | n.a. | n.a. | n.a. |

*Module 2: Survey questionnaire*



For peer review only - http://bmjopen.bmj.com/site/about/guidelines.xhtml

Eligible participants meeting the above criteria will be allocated to one of five study groups depending on their ATS consumption patterns (time, frequency, dependency). Table 2 shows an overview of the operationalisation of the five groups. In order to allow us to distinguish clearly between current and previous use, we defined current use as ATS consumption in the past three months, and former use as no consumption in the past 12 months. This means that participants reporting ATS use over three but less than 12 months previously are excluded.

**Table 2: Operationalization of study groups in Module 2**

| **Study groups in Module 2** | **Name** | **past 12 months prevalence** | **past 3 months prevalence** | **≥ 10 consumption days within past 12 months** | **≥ 10 consumption days within one year (at any time except past 12 months)** |
|---|---|---|---|---|---|
| Group A_1 | Currently frequent users | yes | yes | yes | n.a. |
| Group A_2 | Currently non-frequent users | yes | yes | no | n.a. |
| Group B_1 | Formerly frequent users | no | n.a. | n.a. | yes |
| Group B_2 | Formerly non-frequent users | no | n.a. | n.a. | no |
| Group C | Exposed non-users | n.a. | n.a. | n.a. | n.a. |

For peer review only

## Sample size

*Module 1: Qualitative semi-structured interviews*

45 persons per study group (n=270 for the total sample, see table 3) were considered sufficient for Module 1[43]. As we plan to recruit participants via purposeful sampling[44], we expect to generate data that are rich enough to answer our research questions and inform the questionnaire used in Module 2 of the study.

**Table 3: Sample sizes Module 1 by countries and study groups**

| **Country** | **Partner institution** | **Data collection regions** | **Sample sizes** | | | | | | |
|---|---|---|---|---|---|---|---|---|---|
| | | | Group 1 | Group 2 | Group 3 | Group 4 | Group 5 | Group 6 | **Total N countries** |
| | | | Currently dependent | Formerly depen-dent | Currently frequent, non-dependent | Formerly frequent, non-dependent | Non-frequent (currently or formerly) | Exposed | |
| Germany | ZIS | Border Region to Czech Republic /metropolitan region of Hamburg | 10 | 10 | 10 | 10 | 10 | 10 | **60** |
| United Kingdom | UNEW | Northern England | 10 | 10 | 10 | 10 | 10 | 10 | **60** |
| Poland | APS | Metropolitan region of Warsaw | 10 | 10 | 10 | 10 | 10 | 10 | **60** |
| Netherlands | RG | Amsterdam/the region of Eindhoven | 10 | 10 | 10 | 10 | 10 | 10 | **60** |
| Czech Republic | OGCR | Border region to Germany | 5 | 5 | 5 | 5 | 5 | 5 | **30** |
| **Total N groups** | | | **45** | **45** | **45** | **45** | **45** | **45** | **270** |

*Module 2 Survey questionnaire*



For peer review only - http://bmjopen.bmj.com/site/about/guidelines.xhtml

Statistical analyses within the group of current users (A_1 and A_2) as well as within the group of former users (B_1 and B_2) are planned (see table 2). To facilitate this at country level, a group size of n=100 is sufficient to detect statistically significant small to medium effects for continuous distributed variables (d≥.40, α=0.05, power=80%)[45]. The sample sizes of the groups are planned as follows. For both, groups A and B, up to 200 participants in each country will be recruited. This allows us to analyse subgroups, e.g. dependent and non-dependent users in groups A as well as B. As group C consists of non-users only, the analyses will focus on comparison with one of the user groups (A or B), reducing the required size for this group to n=100. The total sample size for Module 2 for all countries will amount to 2000 persons (see table 4). Due to funding restraints, the sample size in the Netherlands and the Czech Republic is smaller, which might impede the production of statistically significant country specific intra-group comparisons.

For peer review only

**Table 4: Sample sizes Module 2 by countries and study groups**

| Country | Partner institution | Data collection regions | Sample sizes | | | | | |
|---|---|---|---|---|---|---|---|---|
| | | | Current ATS user | | Former ATS user | | Non ATS user | Total N countries |
| | | | A_1: Frequent | A_2: Non-frequent | B_1: Frequent | B_2: Non-frequent | C: exposed | |
| Germany | ZIS | Border Region to Czech Republic /metropolitan region of Hamburg | 100 | 100 | 100 | 100 | 100 | **500** |
| United Kingdom | UNEW | Northern England | 100 | 100 | 100 | 100 | 100 | **500** |
| Poland | APS | Metropolitan region of Warsaw | 100 | 100 | 100 | 100 | 100 | **500** |
| Netherlands | RG | Amsterdam/the region of Eindhoven | 50 | 50 | 50 | 50 | 50 | **250** |
| Czech Republic | OGCR | Border region to Germany | 50 | 50 | 50 | 50 | 50 | **250** |
| **Total N groups** | | | **400** | **400** | **400** | **400** | **400** | **2000** |

## Recruitment

### Sampling method and recruitment procedure

Participants in both Modules will be recruited by non-probability (convenience) sampling, which is an accepted means of accessing participants from 'hard-to-reach' as well as minority populations[46 47]. Pre-identified sampling areas in each participating country (see table) are designed to include participants living in urban as well as rural areas.

Multiple methods and sites will be employed to identify and recruit participants for both Modules. Leaflets and posters containing information about the study, a link to the screening website, and contact details of the research teams, will be printed and distributed in substance use help and treatment facilities, on university black boards, in head shops, bars and nightclubs. Social media and substance use web forums will be used to share and circulate the request for participants. Participants will also be recruited actively at university campuses, drug service facilities and music festivals by directly approaching potential



For peer review only - http://bmjopen.bmj.com/site/about/guidelines.xhtml

participants. All interested interviewees will be screened for eligibility prior to the interview. If eligible to participate, each respondent will receive a screening-code, and the interview can either be conducted straight away, or an appointment can be made for a later date. At the end of each interview participants will be asked to recruit further participants from their social network. This additional snowball sampling approach will be realized by handing over study leaflets, as well as up to three numbered cards containing contact details of the research team. The numbers on the cards will help to track the snowball sampling approach.

## Procedure

### Fieldwork

#### *Module 1*

Face-to-face semi-structured interviews will be conducted by members of the research team of each participating country. Prior to the interview, all participants will receive an information leaflet containing details about the study, explaining what participation involves: anonymity, confidentiality, the use of data, and data protection rules. The participants will then be invited to complete a verbal consent form, should they wish to participate. The interview will be audio recorded and will last approximately 45 to 60 minutes. On completion of the interview, each participant will receive an incentive (money or vouchers, depending on country). All interviews will be transcribed in full, transferred to appropriate software for analysis, and the audio file deleted.

#### *Module 2*

The quantitative survey will be conducted with the CAPI method (Computer-assisted personal interviewing). The questionnaire content will first be developed in Microsoft Word. Once finalised, all questions will be programmed using survey software GessQ® to enable administration via password-protected tablet computers. The survey instrument will be translated and piloted in all partner countries and revised as necessary. The latest version of the questionnaire will be hosted on a central server operating in the IT environment of Hamburg University and can be downloaded to the tablets directly as necessary. This server is also the recipient for the data uploads from the tablets.

In each country, trained research assistants will recruit participants and conduct the interviews face-to-face or via video-telephony (Skype®). Show cards containing relevant prompts and additional information (e.g. lists of ATS, answering scales) will support the conduct of the interviews.

### Monitoring and data management

The screening process and fieldwork progress will be monitored using a study-specific coordination database. This database will be populated with screening data from each participant as well as key information regarding the interviews or questionnaires, such as



For peer review only - http://bmjopen.bmj.com/site/about/guidelines.xhtml

study group, duration of interview, gender distribution, sampling region, and contact with drug help services.

*Module 1*

The raw qualitative data from Module 1 will be managed, stored and analysed by the respective research teams in each participating country. Initial analyses will be conducted at country level using a common coding framework. Analysed data, including emerging interview themes, will be pooled, and provided to ATTUNE PI (Hamburg University).

*Module 2*

The raw quantitative data will be uploaded continuously to the central data management at the Hamburg University. The raw data will be cleaned, edited and transformed into SPSS datasets, one for each country, as well as one comprehensive dataset covering all five countries.

## Survey instruments

*Module 1: Interview guideline and time sheet*

Two semi-structured interview guides will be used to conduct the in-depth interviews in Module 1: one for the ATS user groups plus an adapted topic guide for the non-user group. The topic guide will be based on key emergent themes from the systematic literature review as well as relevant theoretical considerations (biopsychosocial model of substance use). Participants will be asked about their experiences and their consumption patterns regarding ATS and other licit and illicit drugs. To obtain a detailed understanding of which influences have shaped these ATS use patterns, participants will be asked about drug use motives, effects/consequences, settings and occasions, and how these have changed during the period(s) in life where ATS use took place. The interview will end with questions about the (social) setting of use and its impact and the integration of ATS use into the respondent´s lifestyle. The interview guidelines are provided in the supplementary material. Whilst the use of the interview guide will ensure that all central topics are covered, participants will have the opportunity to discuss additional issues or concerns where relevant. During the interviews, researchers will chart participants substance use over time, including age of onset, frequency and life stage, as well as positive and negative life events (e.g. family/partnership, education/work, illness, treatment, imprisonment).

*Module 2: Quantitative questionnaire*

The quantitative survey questionnaire will include questions based on the key themes emerging from the qualitative interviews, as well as a selection of standardised instruments to assess various substance use, health and psychological factors (see table 5).

**Table 5: Overview of standardised measurement instruments**

| Name | Acronym | Content | Reliability: Cronbach´s α | Validity: sensitivity/specificity (cut-off) |
|---|---|---|---|---|
| International Standard Classification of Education[48] | ISCED | Identification of highest educational level | - | - |



For peer review only - http://bmjopen.bmj.com/site/about/guidelines.xhtml

| | | | | |
|---|---|---|---|---|
| Subjective social integration[49] | SSI | Subjective assessment of social integration | - | - |
| Subjective social position[49] | SSP | Subjective assessment of social position | - | - |
| CAGE questionnaire[50] | CAGE | Alcohol problems lifetime | 0.8-0.98 | 0.71/0.90 (2) |
| Alcohol Use Disorders Identification Test[51] | AUDIT-C | Alcohol problems past year | 0.91 | 0.93/0.66 (4) |
| The Severity of Dependence Scale[41] | SDS | ATS dependency lifetime | 0.81–0.89 | 71.3/77.1 (4) |
| Brief Symptom Inventory-18[52] | BSI-18 | Measurement of somatization, anxiety, depression | 0.87-0.94 | 91.2/92.6 (63) |
| Satisfaction with life scale[53] | SWLS | General life satisfaction | - | - |
| Big Five Inventory[54] | BFI-10 | Assessment of five personality traits | 0.58-0.84 | - |
| Brief Sensation Seeking Scale[55] | BSSS-4 | Measurement of sensation seeking | 0.66 | - |
| Generalized Self-Efficacy scale[56] | GSE | Measurement of self-efficacy | 0.92 | - |
| Connor-Davidson Resilience Scale[57] | CD-RISC-10 | Measurement of resilience | 0.89 | - |

The questionnaire will cover:

a. Socio-demographics: These include sex, age, citizenship, migration background, relationship and children, living situation, educational (ISCED) and occupational situation, and social integration (SSI).
b. Drug use: Detailed assessment of all illicit drugs ever used in life (lifetime prevalence, past 12-month prevalence, past 30 days prevalence, age at first and last use), test for alcohol dependence (CAGE, AUDIT-C) and tobacco smoking status.
c. ATS use: Test for ATS dependence (SDS), injecting drug use and treatment experiences; usual setting of ATS use; patterns, motives and consequences of ATS use including (reasons for) changes.
d. Judicial problems: times and reasons for imprisonment
e. Physical and mental health assessment (BSI-18)
f. Personality assessment (BFI-10, BSSS-4, GSE, CD-RISC-10) and critical life events

For group C (non-users), some questions will be omitted (e.g. questions about ATS use), and replaced with alternative questions focussed on their motives for non-use and exposure situation.

## Planned analyses

*Module 1*

Qualitative data from the semi-structured interviews will be analysed using content analysis, and conducted with appropriate software, such as MAXQDA[58] or NVivo[59]. Partners will develop a common, unified coding system to facilitate comparable findings from the interviews across all partner countries. In addition, the data collected with the timeline and chart on substance use and life events will be merged and analysed systematically. Each partner will conduct initial country level analysis separately, in the respective national language. Once this initial analysis has been completed, each partner will produce a report detailing the country-level findings, in English and following a common template. These

For peer review only



For peer review only - http://bmjopen.bmj.com/site/about/guidelines.xhtml

reports will then be compiled and synthesised to produce a comprehensive, cross-national analysis of the qualitative interviews.

*Module 2*

The quantitative data analysis will be conducted using the statistical software package SPSS 22[60]. Descriptive, univariate analyses will be used to describe the socio-demographics, health and personality assessment characteristics of the sample, alongside “consumption careers” (substance use, motives of use, and changes of use patterns). Independent sample t-tests and $\chi^2$-analyses, corrected for multiple testing, will be conducted to compare gender and age characteristics among the ATS user groups. Multivariate approaches will be used to assess a wide range of factors derived from the biopsychosocial model regarding their possible influence on ATS use patterns. So, for example, by calculating a MANOVA, we can simultaneously test if an individual factor like resilience, a social factor like social integration, and an environmental factor like ATS availability is associated with the number of ATS consumption days, the number of cannabis consumption days and the mental health condition index. Furthermore, the MANOVA allows us to detect interactions between the dependent variables. When it comes to an exploration of different ATS user groups such as frequent users, non-frequent users and non-users, we will apply a multinomial logistic regression and determine the association between e.g. traits and being a member of one of those groups. If data permit, we will calculate latent class analyses to identify ATS user groups which characteristics are unknown yet. Analyses will be conducted at country level, as well as across the full European sample.

**Patient and Public Involvement**

Patients and members of the public will be involved in ATTUNE at various stages of the study. Policymakers, European non-governmental organisations and service users helped shape the design and focus of the study prior to obtaining funding. We will hold information/discussion sessions about the study with statutory and non-statutory service providers to acquire their insights into how the findings could potentially impact and shape the everyday lives of their service users. Recruited participants enrolled into the study will be invited to act as ‘seeds’ for the snowball sampling of additional survey interview subjects. This inclusion of patients/public in this way not only helps with enhanced recruitment, but also enables these participants to share their experiences of taking part with others and to underline the importance of the study to people like themselves. ATS user representatives and public representatives will be actively involved in disseminating the results of the research.

## Ethics and dissemination

Based on the regulations in each participating country, ethical approval was obtained. Participant anonymity will be maintained in both the semi-structured interviews and the



For peer review only - http://bmjopen.bmj.com/site/about/guidelines.xhtml

survey questionnaire. During the survey, no information will be collected that could link the data to the participant concerns. All participants will be explicitly asked to provide informed consent to taking part in the study and made aware of the data protection rules. A written informed consent form, signed by the project leader, will be made available to each participant before the interview. Verbal consent in Module 1 will then be asked for, recorded, and documented in the transcription of the interview. The quantitative interview (Module 2) starts with the question, if the participant has read the informed consent form and if she/he is willing to give consent. The information about each participant´s consent will be saved in a dedicated variable of the dataset.

Research findings of ATTUNE will be disseminated in peer reviewed, open access journals as well as at national and international conferences and workshops. Each partner will also deliver reports to their funding institutions under the specific terms of the respective country. Additionally, an accessible report will be drafted and distributed to organisations who express an interest in the study. A specific report that is accessible to substance users will be developed for individuals. We intend to disseminate these findings through social media in order to maximise impact and expand networks of interest.

**Strengths and limitations**

By using multiple methods (systematic literature reviews, qualitative interviews and survey questionnaires), this study will generate in-depth, contextualised evidence in an under-explored field of research. The use of stratified sampling will ensure a sufficient variety of types of ATS users and non-users are included, reflecting different use patterns, current and ex-users, dependent and non-dependent users as well as exposed non-users. In particular, the views and experiences of non-users are rarely reflected in the evidence base, and could help generate novel insights into which factors shape decisions not to consume ATS. The application of standardised interview guides and questionnaires will result in a large comprehensive sample that will allow us to compare ATS use in multiple and varied socio-cultural, political and legal environments across Europe.

A key limitation of the study is the cross-sectional design, which makes it difficult to trace pathways and trajectories of ATS use over time. We address this by only including persons, whose first contact with ATS consumption occurred at least five years ago, meaning they have had the chance to develop different ATS use patterns. However, it is important to stress that a cross-sectional design allows correlative but no causal conclusions. One further issue concerns the convenience sampling approach employed in this study in predefined sampling regions. At the same time, compared to representative general population surveys, our method should ensure increased levels of inclusion of different ATS user types, allowing for more detailed and in-depth insights into different use trajectories. Finally, by defining multiple inclusion criteria as well as precise group strata, we aim to mitigate potential bias that could emerge from this sampling approach.



For peer review only - http://bmjopen.bmj.com/site/about/guidelines.xhtml

## Implications for interventions and future policy

The findings from this research will enable policy makers and practitioners to improve existing ATS prevention and intervention programmes, and support the development of new approaches in the future. By examining different types of stimulant users (including ex-users and non-users), information will be generated which will be important for universal prevention (targeting general populations), selective prevention (focussing on vulnerable groups), and indicated prevention (aiming at groups that show early signs of problematic substance use). Furthermore, given the limited long-term efficacy of ATS treatment[61], the findings of this study could support the development of ATS treatment programmes that are more effectively tailored to the needs of specific ATS populations and individual users.

*Authors' contribution:* MR, MSM, HZ, UV, AOD and MA planned and designed the study. All other authors contributed to the design of the study. UV is the principal investigator; MSM and MR are the overall project managers and coordinators of the study. Country specific study coordination in the United Kingdom is performed by AOD, MA and EK, in the Netherlands by NL, in Czech Republic by MB and BP, in Poland by MRow, and in Germany by MR, MSM, HZ and UV. MR wrote the first draft of the study protocol and led the revisions of the manuscript with substantial critical input from all co-authors. In addition to crucial input as regards content, AOD provided significant help to improve the article´s language. All authors read and approved the final version of the manuscript.

*Acknowledgements:* We thank all policymakers, European non-governmental organisations and service users that helped shape the design and focus of the study.

*Funding:* The superordinate research framework ERANID is funded by the European Union under the 7th Framework Programme. Each project partner receives funding from its national funding bodies: United Kingdom: National Institute for Health Research (NIHR) Policy Research Programme (project ref. PR-ST-0416-10001); Germany: Bundesministerium für Gesundheit (project ref. ZMVI1-2516DSM222); Czech Republic: Office of the Government of the Czech Republic (Decision No. 10701635 / 18- OPK) and Charles University (No. PROGRES Q06/LF1); Poland: the National Bureau for Drug Prevention; the Netherlands: ZonMw.

*Competing interests:* Uwe Verthein received a speaker's honoraria and travelling expenses from Mundipharma GmbH. Amy O'Donnell was funded by an NIHR School for Primary Care Research Fellowship between October 2015 and September 2017. All other authors declared not to have competing interests.

*Ethics approval:* In GER, UK, PL, CZ, the study has been reviewed and approved by the respective responsible ethics committee, in NL no ethical approval was required. The respective names of all ethics committees as well as reference numbers are as follows: GER: Ethics Committee of the Hamburg Medical Chamber WF-03/17; UK: National Health Service Health Research Authority North East - Newcastle and North Tyneside 2 Research Ethics Committee 17/NE/0283; PL: Academy of Special Education Ethical Committee 168-



For peer review only - http://bmjopen.bmj.com/site/about/guidelines.xhtml

2018/2019; CZ: Ethical Committee of the National Monitoring Centre for Drugs and Addiction 180326_EK-NMS.

For peer review only



For peer review only - http://bmjopen.bmj.com/site/about/guidelines.xhtml

## REFERENCES

1. UNODC. United Nations Office On Drugs And Crime. World Drug Report 2018. Vienna: United Nations, 2018.
2. EMCDDA. European Monitoring Centre for Drugs and Drug Addiction. European Drug Report 2018: Trends and Developments. Publications Office of the European Union. Luxembourg: European Monitoring Centre for Drugs and Drug Addiction, 2018.
3. EMCDDA. EMCDDA –Europol joint publications: Amphetamine. Lisbon: European Monitoring Centre for Drugs and Drug Addiction 2011.
4. EMCDDA. European Monitoring Centre for Drugs and Drug Addiction and Eurojust. New psychoactive substances in Europe: Legislation and prosecution. Lisbon: EMCDDA & Eurojust, 2017.
5. UNODC. United Nations Office On Drugs And Crime. World Drug Report 2017. Vienna: United Nations, 2017.
6. EMCDDA. European Monitoring Centre for Drugs and Drug Addiction: Wastewater analysis and drugs: a European multi-city study. Perspectives on Drugs. Lisbon, 2018.
7. Best D, Gross S, Vingoe L, et al. Dangerousness of Drugs: A Guide to the Risks and Harms Associated with Substance Use2003.
8. Degenhardt L, Charlson F, Ferrari A, et al. The global burden of disease attributable to alcohol and drug use in 195 countries and territories, 1990–2016: a systematic analysis for the Global Burden of Disease Study 2016. *The Lancet Psychiatry* 2018;5(12):987-1012. doi: 10.1016/S2215-0366(18)30337-7
9. Gonzales R, Mooney L, Rawson R. The Methamphetamine Problem in the United States. *Annual Review of Public Health* 2010;31(1):385-98. doi: 10.1146/annurev.publhealth.012809.103600
10. Degenhardt L, Degenhardt L, Roxburgh A, et al. The epidemiology of methamphetamine use and harm in Australia. *Drug and Alcohol Review* 2008;27(3):243-52. doi: 10.1080/09595230801950572
11. Kelly BC. Mediating MDMA-Related Harm: Preloading and Post-loading Among Ecstasy-Using Youth. *Journal of Psychoactive Drugs* 2009;41(1):19-26. doi: 10.1080/02791072.2009.10400671
12. Home Office. Psychoactive substances ban 6 months on: almost 500 arrests and first convictions. . Retrieved from https://www.gov.uk/government/news/psychoactive-substances-ban-6-months-on-almost-500-arrests-and-first-convictions, 2016.
13. Addison M, Stockdale K, McGovern R, et al. Exploring the intersections between novel psychoactive substances (NPS) and other substance use in a police custody suite setting in the north east of England. *Drugs: Education, Prevention and Policy* 2017:1-7. doi: 10.1080/09687637.2017.1378620



For peer review only - http://bmjopen.bmj.com/site/about/guidelines.xhtml

14. O'Donnell A, Addison M, Spencer L, et al. Which individual, social and environmental influences shape key phases in the amphetamine type stimulant use trajectory? A systematic narrative review and thematic synthesis of the qualitative literature. *Addiction (Abingdon, England)* 2018 doi: 10.1111/add.14434 [published Online First: 2018/09/04]
15. Duff C, Moore D. Evading and embracing normality: estrangement and ambivalence in the accounts of methamphetamine consumers. *Critical Public Health* 2015;25(4):488-503. doi: 10.1080/09581596.2014.913785
16. Levy KB, O'Grady KE, Wish ED, et al. An in-depth qualitative examination of the ecstasy experience: results of a focus group with ecstasy-using college students. *Substance use & misuse* 2005;40(9-10):1427-41. doi: 10.1081/ja-200066810 [published Online First: 2005/07/29]
17. Van Hout MC, Brennan R. Plant food for thought: A qualitative study of mephedrone use in Ireland. *Drugs: Education, Prevention and Policy* 2011;18(5):371-81. doi: 10.3109/09687637.2010.537713
18. Ding Y, He N, Detels R. Circumstances of initiation into new-type drug use among adults in Shanghai: are there differences by types of first new-type drug used? *Drug and alcohol dependence* 2013;131(3):278-83. doi: 10.1016/j.drugalcdep.2012.12.019 [published Online First: 2013/01/15]
19. Boeri MW, Harbry L, Gibson D. A Qualitative Exploration of Trajectories Among Suburban Users of Methamphetamine. *Journal of ethnographic and qualitative research* 2009;3(3):139-51. [published Online First: 2009/01/01]
20. Fast D, Small W, Wood E, et al. Coming 'down here': young people's reflections on becoming entrenched in a local drug scene. *Social science & medicine (1982)* 2009;69(8):1204-10. doi: 10.1016/j.socscimed.2009.07.024 [published Online First: 2009/08/25]
21. Sherman SG, German D, Sirirojn B, et al. Initiation of methamphetamine use among young Thai drug users: a qualitative study. *The Journal of adolescent health : official publication of the Society for Adolescent Medicine* 2008;42(1):36-42. doi: 10.1016/j.jadohealth.2007.07.002 [published Online First: 2007/12/25]
22. Cheney AM, Newkirk CN, Nekhavhambe VM, et al. Effects of social and spatial contexts on young latinas' methamphetamine use initiation. *Journal of ethnicity in substance abuse* 2018;17(1):32-49. doi: 10.1080/15332640.2017.1362721 [published Online First: 2017/10/17]
23. Smirnov A, Najman JM, Hayatbakhsh R, et al. Young adults' recreational social environment as a predictor of ecstasy use initiation: findings of



For peer review only - http://bmjopen.bmj.com/site/about/guidelines.xhtml

a population-based prospective study. *Addiction (Abingdon, England)* 2013;108(10):1809-17. doi: 10.1111/add.12239 [published Online First: 2013/05/15]

24. Cheng WS, Garfein RS, Semple SJ, et al. Binge use and sex and drug use behaviors among HIV(-), heterosexual methamphetamine users in San Diego. *Substance use & misuse* 2010;45(1-2):116-33. doi: 10.3109/10826080902869620 [published Online First: 2009/12/23]
25. Boeri MW, Sterk CE, Elifson KW. Baby Boomer Drug Users: Career Phases, Social Control, and Social Learning Theory. *Sociological Inquiry* 2006;76(2):264-91. doi: doi:10.1111/j.1475-682X.2006.00154.x
26. Ho HT, Le GM, Dinh TT. Female sex workers who use amphetamine-type stimulants (ATS) in three cities of Vietnam: use and sexual risks related to HIV/AIDS. *Global public health* 2013;8(5):552-69. doi: 10.1080/17441692.2013.790459 [published Online First: 2013/05/18]
27. von Mayrhauser C, Brecht ML, Anglin MD. Use ecology and drug use motivations of methamphetamine users admitted to substance abuse treatment facilities in Los Angeles: an emerging profile. *Journal of addictive diseases* 2002;21(1):45-60. [published Online First: 2002/02/08]
28. Bourne A, Reid D, Hickson F, et al. "Chemsex" and harm reduction need among gay men in South London. *The International journal on drug policy* 2015;26(12):1171-6. doi: 10.1016/j.drugpo.2015.07.013 [published Online First: 2015/08/25]
29. Eiserman JM, Diamond S, Schensul JJ. "Rollin' on E": a qualitative analysis of ecstasy use among inner city adolescents and young adults. *Journal of ethnicity in substance abuse* 2005;4(2):9-38. doi: 10.1300/J233v04n02_02 [published Online First: 2005/11/09]
30. Parsons JT, Kelly BC, Weiser JD. Initiation into methamphetamine use for young gay and bisexual men. *Drug and alcohol dependence* 2007;90(2-3):135-44. doi: 10.1016/j.drugalcdep.2007.02.017 [published Online First: 2007/04/03]
31. Carbone-Lopez K, Owens JG, Miller J. Women's "Storylines" of Methamphetamine Initiation in the Midwest. *Journal of Drug Issues* 2012;42(3):226-46. doi: 10.1177/0022042612456013
32. Kerley KR, Copes H, Griffin OH. Middle-Class Motives for Non-Medical Prescription Stimulant Use among College Students. *Deviant Behavior* 2015;36(7):589-603. doi: 10.1080/01639625.2014.951573
33. Bavarian N, Flay BR, Ketcham PL, et al. Illicit use of prescription stimulants in a college student sample: a theory-guided analysis. *Drug and alcohol dependence* 2013;132(3):665-73. doi:

For peer review only

10.1016/j.drugalcdep.2013.04.024 [published Online First: 2013/05/21]

34. Sterk CE, Theall KP, Elifson KW. Getting into ecstasy: comparing moderate and heavy young adult users. *Journal of psychoactive drugs* 2007;39(2):103-13. doi: 10.1080/02791072.2007.10399869 [published Online First: 2007/08/21]

35. Bungay V, Malchy L, Buxton JA, et al. Life with jib: A snapshot of street youth's use of crystal methamphetamine. *Addiction Research & Theory* 2006;14(3):235-51. doi: 10.1080/16066350500270901

36. German D, Sherman SG, Sirirojn B, et al. Motivations for methamphetamine cessation among young people in northern Thailand. *Addiction (Abingdon, England)* 2006;101(8):1143-52. doi: 10.1111/j.1360-0443.2006.01490.x [published Online First: 2006/07/28]

37. O'Brien AM, Brecht M-L, Casey C. Narratives of Methamphetamine Abuse: A Qualitative Exploration of Social, Psychological, and Emotional Experiences. *Journal of Social Work Practice in the Addictions* 2008;8(3):343-66. doi: 10.1080/15332560802224469

38. Sexton RL, Carlson RG, Leukefeld CG, et al. Trajectories of Methamphetamine Use in the Rural South: A Longitudinal Qualitative Study. *Human Organization* 2008;67(2):181-93.

39. Engel GL. The need for a new medical model: a challenge for biomedicine. *Science (New York, NY)* 1977;196(4286):129-36. [published Online First: 1977/04/08]

40. Cooke A, Smith D, Booth A. Beyond PICO: the SPIDER tool for qualitative evidence synthesis. *Qualitative health research* 2012;22(10):1435-43. doi: 10.1177/1049732312452938 [published Online First: 2012/07/26]

41. Gossop M, Darke S, Griffiths P, et al. The Severity of Dependence Scale (SDS): psychometric properties of the SDS in English and Australian samples of heroin, cocaine and amphetamine users. *Addiction (Abingdon, England)* 1995;90(5):607-14. [published Online First: 1995/05/01]

42. Bruno R, Matthews AJ, Topp L, et al. Can the severity of dependence scale be usefully applied to 'ecstasy'? *Neuropsychobiology* 2009;60(3-4):137-47. doi: 10.1159/000253550 [published Online First: 2009/11/07]

43. Malterud K, Siersma VD, Guassora AD. Sample Size in Qualitative Interview Studies: Guided by Information Power. *Qualitative health research* 2015 doi: 10.1177/1049732315617444 [published Online First: 2015/11/29]

44. Palinkas LA, Horwitz SM, Green CA, et al. Purposeful sampling for qualitative data collection and analysis in mixed method



For peer review only - http://bmjopen.bmj.com/site/about/guidelines.xhtml

implementation research. *Administration and policy in mental health* 2015;42(5):533-44. doi: 10.1007/s10488-013-0528-y
45. Cohen J. Statistical power analysis for the behavioral sciences. 2 ed. Hillsdale, NJ, England: Lawrence Erlbaum Accociates 1988.
46. Etikan I, Musa Sa, Alkassim RS. Comparison of Convenience Sampling and Purposive Sampling. *American Journal of Theoretical and Applied Statistics* 2016;5(1):1-4. doi: 10.11648/j.ajtas.20160501.11
47. Shaghaghi A, Bhopal RS, Sheikh A. Approaches to Recruiting 'Hard-To-Reach' Populations into Research: A Review of the Literature. *Health promotion perspectives* 2011;1(2):86-94. doi: 10.5681/hpp.2011.009
48. UNESCO. International Standard Classification of Education (ISCED) 2011 [Available from: http://uis.unesco.org/en/topic/international-standard-classification-education-isced accessed 05.12.2018.
49. Beste J, Bethmann A, Gundert S. Social Structure and Living Conditions: Material and Social Situation of Unemployment Benefit Recipients. In: Research IfE, ed. IAB Summary Reports. Nuremberg, 2014:8.
50. Ewing JA. Detecting alcoholism. The CAGE questionnaire. *Jama* 1984;252(14):1905-7. [published Online First: 1984/10/12]
51. Bush K, Kivlahan DR, McDonell MB, et al. The AUDIT alcohol consumption questions (AUDIT-C): an effective brief screening test for problem drinking. Ambulatory Care Quality Improvement Project (ACQUIP). Alcohol Use Disorders Identification Test. *Archives of internal medicine* 1998;158(16):1789-95. [published Online First: 1998/09/17]
52. Derogatis LR. BSI 18, Brief Symptom Inventory 18 : administration, scoring and procedures manual. Minneapolis, MN: NCS Pearson, Inc. 2001.
53. Diener E, Emmons RA, Larsen RJ, et al. The Satisfaction With Life Scale. *Journal of personality assessment* 1985;49(1):71-5. doi: 10.1207/s15327752jpa4901_13 [published Online First: 1985/02/01]
54. Rammstedt B, John OP. Measuring personality in one minute or less: A 10-item short version of the Big Five Inventory in English and German. *Journal of Research in Personality* 2007;41(1):203-12. doi: https://doi.org/10.1016/j.jrp.2006.02.001
55. Stephenson MT, Hoyle RH, Palmgreen P, et al. Brief measures of sensation seeking for screening and large-scale surveys. *Drug and alcohol dependence* 2003;72(3):279-86. [published Online First: 2003/12/04]
56. Schwarzer R, Jerusalem M. Generalized Self-Efficacy scale. In: Weinman J, Wright S, Johnston M, eds. Measures in health psychology: A user's portfolio Causal and control beliefs. Windsor, England: NFER-NELSON. 1995:35-37.



For peer review only - http://bmjopen.bmj.com/site/about/guidelines.xhtml

57. Connor KM, Davidson JR. Development of a new resilience scale: the Connor-Davidson Resilience Scale (CD-RISC). *Depression and anxiety* 2003;18(2):76-82. doi: 10.1002/da.10113 [published Online First: 2003/09/10]
58. MAXQDA [program]. 18.1.1 version. Berlin: VERBI Software GmbH, 2018.
59. NVivo [program]. Victoria, Australia: QSR International 2017.
60. SPSS 22 [program]. New York: IBM, 2013.
61. Minozzi S, Saulle R, De Crescenzo F, et al. Psychosocial interventions for psychostimulant misuse. *The Cochrane database of systematic reviews* 2016;9:Cd011866. doi: 10.1002/14651858.CD011866.pub2 [published Online First: 2016/09/30]

For peer review only



For peer review only - http://bmjopen.bmj.com/site/about/guidelines.xhtml

**Use for groups 1-5:**
dependent; remitted; frequent; non-dependent; formerly frequent non-dependent, non-frequent

# Interview Guideline 'A' – Attune

**Interviewer:**

This study is about understanding pathways to stimulants use across Europe. You have agreed to participate in an interview as part of this study because you feel you would identify with one of the following statements, would you agree?

a) Are **currently** using amphetamines (Group 1, 2, 3 and 5)
b) **Stopped** using amphetamines (Group 2, 4 and 5)
c) Never used amphetamines but have had the **opportunity** to in the past (Group 6 – USE Guideline B)

## Introduction

**Tell me a little about yourself and your life now.**

(Prompts – Age, individual situation (job, housing, income, education, health situation, emotional well-being), social life (partnership, family, friends) positive and negative impacts)

## Drugs and alcohol

**What is your experience of drug and alcohol use? [Interviewer use CHART tool** for reporting on substances used in life until today]

(Prompts: *personal usage including: age of first use; type of drugs, frequency and quantity, motivations for use/non-use, levels and routes of use; negative impact (dependence, offending, imprisonment)*

**What effect are you hoping for from use of these substances?**

(Prompts: socialising, coping strategy, behaviour change, health)

**Tell me more about the circumstances of your drug and alcohol use since your first use [**plot significant moments in the **life events CHART**]

*(Prompts: use of drugs/alcohol by others around them; any drug/alcohol offers; individual situation (work, housing, income, health), relationships (family, friends, romantic partners); critical life events (violence, separation from significant other, death, illness, chronic disease)*

**What is your use of drug and alcohol like now? Has this changed over time? [CHART tool for substances]**

(Prompts: *type of substance and frequency of use;*
circumstances for changes (living, employment, education, income, health), lifestyle (leisure), relationships (friends, family, romantic partner, marriage, birth of children)

## Stimulants – Firsts, effects, perceptions

**What is your experience of Amphetamine Type Substances? E.g. amphetamine, ecstasy, MDMA, methamphetamine [**REFER to ATS card and CHART for substances]

(Prompts:, , *personal usage including: age of first use; type of drugs; frequency levels and routes of use; and surrounding circumstances of use/non-use, motivations for use/non-use)*

For peer review only

**Use for groups 1-5:**
dependent; remitted; frequent; non-dependent; formerly frequent non-dependent, non-frequent

**What was life like leading up to Amphetamine Type Stimulants being first used? [**plot significant moments in the **life events CHART]**

(Prompt around family, friendships, relationships, education, employment, and social, offending behaviour, health and emotional well-being, housing, significant events)

**What effect were you hoping for from these Amphetamine Type Stimulants? Why did you want this effect?**

(Prompt: feelings, positive and negative experiences, change inhibitions, focus, control, euphoria)

**What was happening in your life when you continue to use XXX (specify the different ATS the person has discussed)? [**plot significant moments in the **life events CHART]**

(Prompts: *use of ATS by others around them, any ATS offers or availability* people in their social network and their use/non-use of ATS, *individual situation (work, housing, income, health), relationships (family, friends, romantic partners); critical life events (violence, separation from significant other, death, illness, chronic disease)*

**What do you personally think about ATS use?**

(Prompts around positive and negative views- physical and mental health, lifestyle, personal plans, offending, social relations)

## Stimulants – Source, availability, funding

**How would/do you usually get Amphetamine Type Stimulants?**
(Prompt: buy from/share with friends, dealer-user interactions, ease of interaction/introduction to dealer)

**How easy or difficult are they to get? Give examples**

**How do you fund your use? Does funding imply any legal problems?**
(Prompt: employment, welfare, criminal activity, deal, trouble with the police, criminal justice system)

## Stimulants – lifestyle and relationships

**How does your ATS use impact on your relationships with others? Give examples**

**How has your lifestyle changed since you started/stopped using ATS? [**plot significant moments in the **life events CHART]**

(**Prompt:** significant life events (individual, social, lifestyle, health and well-being), increases/decreases in substance use since first usage of ATS; protective or harmful factors (dependence, offending)

**How has your use of ATS changed over time? [Plot CHART for substances]**

(Prompt: *type of substance and frequency of use,* increase in levels, decrease in levels, poly-substance use, changes in routes of administration, context of use

**What was happening at the time of the change in use**? [plot significant moments in the **life events CHART]**

(Prompt:, treatment, other service use, circumstances (living, employment, education, income, health), lifestyle (leisure), relationships (friends, family, romantic partner, marriage, birth of children), significant events

**What is important for your life now?**

For peer review only

**Use for groups 1-5:**
dependent; remitted; frequent; non-dependent; formerly frequent non-dependent, non-frequent

(Prompt around family, marriage, children, romantic partners, friends, education, employment, social, offending behaviour, health and emotional well-being, housing, positive and negative impacts)

**What do you hope for/expect for your life in the next 5-10 years?**

(Prompt: change in substance use (including ATS), change in relationships, health, employment career, lifestyle)

*Thank you for taking part in interview*

(Check have consent form)

For peer review only

**Use for group 6:**
Non-stimulant user

# Interview Guideline 'B' – Attune

**Interviewer:**

This study is about understanding pathways to stimulants use across Europe. You have agreed to participate in an interview as part of this study because you feel you would identify with one of the following statements, would you agree?

a) Are **currently** using amphetamines (Group 1, 2, 3, 5 – use Guideline A)
b) **Stopped** using amphetamines (Group 2, 4 and 5 – use Guideline A)
c) Never used amphetamines but have had the **opportunity** to in the past (Group 6 – USE Guideline B)

## Introduction

**Tell me a little about yourself and your life now.**

(Prompts – Age, individual situation (job, housing, income, education, health situation, emotional well-being), social life (partnership, family, friends) positive and negative impacts)

**What is your experience of drug and alcohol use? [Interviewer use CHART tool** for reporting on substances used in life until today]

(Prompts: *personal usage including: age of first use; type of drugs, frequency and quantity motivations for use/non-use, levels and routes of use; negative impact (dependence, offending, imprisonment)*

**What effect are you hoping for from use of these substances?**

(Prompts: socialising, coping strategy, behaviour change, health)

## Drugs and alcohol

**Tell me more about the circumstances of your drug and alcohol use since your first use [**plot significant moments in the **life events CHART**]

*(Prompts: use of drugs/alcohol by others around them; any drug/alcohol offers; individual situation (work, housing, income, health), relationships (family, friends, romantic partners); critical life events (violence, separation from significant other, death, illness, chronic disease)*

**What is your use of drug and alcohol like now? Has this changed over time? [CHART tool for substances**]

(Prompts: *type of substance and frequency of use;* circumstances (living, employment, education, income, health), lifestyle (leisure), relationships (friends, family, romantic partner, marriage, birth of children)

## Stimulants – Firsts, protective factors, perceptions

**What is your experience with the use Amphetamine Type Substances? E.g. amphetamine, ecstasy, MDMA, methamphetamine in your social network?**

(Prompts: *use of ATS by others around them, any ATS offers or availability)*

For peer review only - http://bmjopen.bmj.com/site/about/guidelines.xhtml

**Use for group 6:**
Non-stimulant user

**What was happening in your life when XXX (specify the different ATS the person has discussed by using the ATS card) was offered to you (ATS substances)? [**plot significant moments in the **life events CHART**]

(Prompt: individual and social circumstances surrounding the non-use health and wellbeing, people in their social network and their use/non-use of ATS, specific life events)

**Are others in your networks continuing to use ATS? How does this use impact on your relationship with them? Give examples**

**(**Prompt: changes, life events, availability)

**What was it like being around others who were using ATS?**

(Prompt: impact on relationships, socialising, networks, and employment)

**Has there ever been a situation where you thought about using ATS? [Plot [**plot significant moments in the **life events CHART**] **What was happening at this time for you?**

(prompt: lifestyle, relationships, networks, employment, significant events)

**How would you explain that you never used these ATS**

(Prompt: costs, availability, opportunity, age, circumstances, health, fear of legal problems, fear of dependence, protective factors such as partner, employment, lifestyle, negative impact on other people)

**What do you think about ATS use? Has that perception changed or stayed the same over time?**

(Prompt around positive and negative views- physical and mental health, lifestyle, personal plans, offending, social relations)

## Stimulants – Source, availability, funding

**Would you know how to buy Amphetamine Type Stimulants?**
(Prompt: buy from/share with friends, dealer-user interactions, ease of interaction/introduction to dealer)

**How easy or difficult do you think they are to get? Give reasons**

## Lifestyle

**What is important for your life now?**

(Prompt around family, marriage, children, romantic partners, friends, education, employment, social, offending behaviour, health and emotional well-being, housing, positive and negative impacts)

**What do you hope for/expect for your life in the next 5-10 years?**

(Prompt: change in substance use (including ATS), change in relationships, health, employment career, lifestyle)

*Thank you for taking part in interview*

(Check have consent form)

For peer review only
